# Efficacy and Safety of High-Dose Ivermectin for Reducing Malaria Transmission (IVERMAL): Protocol for a Double-Blind, Randomized, Placebo-Controlled, Dose-Finding Trial in Western Kenya

**DOI:** 10.2196/resprot.6617

**Published:** 2016-11-17

**Authors:** Menno R Smit, Eric Ochomo, Ghaith Aljayyoussi, Titus Kwambai, Bernard Abong'o, Nabie Bayoh, John Gimnig, Aaron Samuels, Meghna Desai, Penelope A Phillips-Howard, Simon Kariuki, Duolao Wang, Steve Ward, Feiko O ter Kuile

**Affiliations:** ^1^ Liverpool School of Tropical Medicine (LSTM) Liverpool United Kingdom; ^2^ Centre for Global Health Research Kenya Medical Research Institute (KEMRI) Kisumu Kenya; ^3^ Kisumu County Kenya Ministry of Health (MoH) Kisumu Kenya; ^4^ Division of Parasitic Diseases and Malaria Center for Global Health U.S. Centers for Disease Control and Prevention (CDC) Atlanta, GA United States

**Keywords:** malaria, Plasmodium falciparum, ivermectin, dihydroartemisinin-piperaquine, *Anopheles gambiae s.s.*, insecticide, clinical trial, pharmacokinetics, Kenya, study protocol

## Abstract

**Background:**

Innovative approaches are needed to complement existing tools for malaria elimination. Ivermectin is a broad spectrum antiparasitic endectocide clinically used for onchocerciasis and lymphatic filariasis control at single doses of 150 to 200 mcg/kg. It also shortens the lifespan of mosquitoes that feed on individuals recently treated with ivermectin. However, the effect after a 150 to 200 mcg/kg oral dose is short-lived (6 to 11 days). Modeling suggests higher doses, which prolong the mosquitocidal effects, are needed to make a significant contribution to malaria elimination. Ivermectin has a wide therapeutic index and previous studies have shown doses up to 2000 mcg/kg (ie, 10 times the US Food and Drug Administration approved dose) are well tolerated and safe; the highest dose used for onchocerciasis is a single dose of 800 mcg/kg.

**Objective:**

The aim of this study is to determine the safety, tolerability, and efficacy of ivermectin doses of 0, 300, and 600 mcg/kg/day for 3 days, when provided with a standard 3-day course of the antimalarial dihydroartemisinin-piperaquine (DP), on mosquito survival.

**Methods:**

This is a double-blind, randomized, placebo-controlled, parallel-group, 3-arm, dose-finding trial in adults with uncomplicated malaria. Monte Carlo simulations based on pharmacokinetic modeling were performed to determine the optimum dosing regimens to be tested. Modeling showed that a 3-day regimen of 600 mcg/kg/day achieved similar median (5 to 95 percentiles) maximum drug concentrations (Cmax) of ivermectin to a single of dose of 800 mcg/kg, while increasing the median time above the lethal concentration 50% (LC50, 16 ng/mL) from 1.9 days (1.0 to 5.7) to 6.8 (3.8 to 13.4) days. The 300 mcg/kg/day dose was chosen at 50% of the higher dose to allow evaluation of the dose response. Mosquito survival will be assessed daily up to 28 days in laboratory-reared *Anopheles gambiae s.s.* populations fed on patients’ blood taken at days 0, 2 (Cmax), 7 (primary outcome), 10, 14, 21, and 28 after the start of treatment. Safety outcomes include QT-prolongation and mydriasis. The trial will be conducted in 6 health facilities in western Kenya and requires a sample size of 141 participants (47 per arm). Sub-studies include (1) rich pharmacokinetics and (2) direct skin versus membrane feeding assays.

**Results:**

Recruitment started July 20, 2015. Data collection was completed July 2, 2016. Unblinding and analysis will commence once the database has been completed, cleaned, and locked.

**Conclusions:**

High-dose ivermectin, if found to be safe and well tolerated, might offer a promising new tool for malaria elimination.

## Introduction

Ivermectin is a potential new tool that is being considered in malaria transmission reduction strategies [1]. Ivermectin is a broad spectrum antiparasitic endectocide active against a wide range of internal and external parasites. It was originally introduced as a veterinary drug, predominantly for use in domestic livestock, but since 1987 has been widely used in human medicine [2]. Ivermectin at a dose of 150 or 200 mcg/kg is the first-line treatment for *Onchocerca volvulus* (the cause of river blindness) [3], *Wuchereria bancrofti* (the cause of lymphatic filariasis) [4], and *Strongyloides stercoralis* (roundworm, an intestinal helminth) [5]. To date more than 2.7 billion treatments have been distributed as part of mass drug administration (MDA) [6].

Ivermectin has secondary effects on ectoparasites, such as head lice, mites, bedbugs, and scabies, that feed on recently treated individuals [2,7], and it is also active against *Anopheles spp*. at concentrations present in human blood after standard doses. It reduces the re-blood feeding capacity, female fecundity, hatch rate of their eggs, the survival of progeny larvae, and importantly, it reduces the vector’s lifespan [1,8-11]. It may also inhibit parasite sporogony [12]. Ivermectin has a different mode of action from other insecticides, and therefore may be effective against mosquito populations that are resistant to insecticides used on long-lasting insecticidal nets (LLINs) or indoor residual spraying (IRS). Furthermore, it is able to kill exophagic and exophillic vectors that can escape the indoor killing effects of LLINs and IRS [8].

However, several studies have shown that the effects after the standard 150 to 200 mcg/kg doses of ivermectin are generally short-lived. Three *in vivo* studies assessed the long-term effect of ivermectin on mosquito survival by conducting feedings at least 7 days after administration of ivermectin [10,13,14]. A single low dose of 200 mcg/kg showed a 1.33 fold increase in mosquito mortality when fed on blood taken from humans who had received ivermectin 1 day earlier, but there was no longer an effect when mosquitoes were fed on blood taken on day 14 post-treatment [10], while a repeated dose of 200 mcg/kg given on days 0 and 2 showed a modest effect on reduced survival 7 days post-treatment [14], and a dose of 250 mcg/kg in a single human volunteer showed a potent effect for at least 2 weeks post-treatment [13]. Population-based studies of the effect of MDA with ivermectin on malaria transmission or mosquito survival showed that MDA with a single dose of 150 mcg/kg for the control of onchocerciasis in Senegal affected survivorship of *An. gambiae s.s.* for up to 6 days, resulting in an estimated reduction of malaria transmission for at least 11 days as a result of a change in the age-structure of *An. gambiae s.s.* [15-17]. Similarly, in 3 different West African transmission settings, this same dose reduced *An. gambiae* survivorship by 33.9% for 1 week, their parity rates for more than two weeks, and sporozoite rates by more than 77% for 2 weeks [18].

Modeling has also shown that adding 3 days of ivermectin (150 mcg/kg/day) to MDA with dihydroartemisinin-piperaquine (DP) would potentially provide an important boost to the effect of MDAs with artemisinin-based combination therapy (ACT) by allowing transmission to be interrupted faster and in areas with a higher malaria prevalence than MDA with ACTs alone [19]. However, the effects are modest, and higher doses, providing a longer effect are required for ivermectin to boost malaria transmission reduction activities [19].

Ivermectin 400 mcg/kg has been suggested as an improved treatment for head lice [20], and has been found to be safe and well tolerated [21]. No studies in humans have compared the effect of ivermectin doses above 400 mcg/kg on the ability of anopheline vectors to transmit malaria (henceforth referred to as infectivity), or evaluated the effect of any dose of ivermectin higher than 400 mcg/kg on mosquito survivorship.

Ivermectin has an excellent safety profile [1], and experience with higher doses show that it is remarkably well tolerated in humans [22-27], even at doses up to 2000 mcg/kg, 10 times the 200 mcg/kg dose currently approved by the US Food and Drug Administration [24] (Table 1). In invertebrates, ivermectin causes the opening of glutamate-gated chloride channels resulting in flaccid paralysis and death [28]. Glutamate-gated chloride channels do not exist in humans. Other weakly sensitive channels are found in the human central nervous system, but the blood-brain barrier limits drug access to these channels [29].

**Table 1:** Studies of safety and tolerability of ivermectin incorporating dosages greater than or equal to 800 mcg/kg.

Reference	Highest single dose	Participants with single dose ≥800 mcg/kg, n	Total study population, n	Single doses in mcg/kg (n)	Adverse events: increased vs control
Awadzi 1995, 1999 [22,23]	800 mcg/kg	36	100 adult males with onchocerciasis in Ghana	150 (15), 400^a^ (25), 600^a^ (24), 800^a^ (24), 800^b^ (12)	No
Guzzo 2002 [24]	2000 mcg/kg	36	68 healthy adults, non-pregnant, in the United States	0 (17), 500^c^ (15), 1000^c^ (12), 1500 (12), 2000 (12)	No
Kamgno 2004 [25-27]	800 mcg/kg	330	657 adult males with onchocerciasis in Cameroon	150^d^ (327), 800^d,e^ (330)	Transitory mild visual side effects, without structural abnormalities upon ophthalmological exam

^a^Preceded 3 days earlier by 150 mcg/kg or placebo.

^b^Preceded 13 days earlier by 800 mcg/kg.

^c^Repeated 3 times a week (days 1, 4, and 7).

^d^Repeated 3 times monthly or once yearly.

^e^Preceded 3 or 12 months earlier by 400 mcg/kg.

The only known severe adverse events have been in individuals with *Loa loa,* possibly due to rapid lysis of parasite biomass [30]. Assessment of *Loa loa* is recommended before ivermectin administration in areas endemic for *Loa loa* filariasis [31].

DP and ivermectin have, to the best of our knowledge, never been studied under simultaneous administration. Piperaquine, the long-acting component of DP, is metabolized by, and is an inhibitor of, cytochrome-P450 3A4 (CYP3A4) [32]. There is a potential for an increase of piperaquine plasma concentrations when it is co-administered with other CYP3A4 substrates (due to competition) or CYP3A4 inhibitors [32]. Dihydroartemisinin (DHA), the short-acting component of DP, is not metabolized by cytochrome-P450, but is deactivated via glucuronidation catalyzed by UDP-glucuronosyltransferases, in particular UGT1A9 and UGT2B7 [33]. DHA has been shown to induce CYP3A activity and also up-regulate CYP2C19 and CYP2B6 [33]. DHA is a known inhibitor of CYP1A2 [32].

Ivermectin is primarily metabolized by CYP3A4 [34]. *In vitro* studies using human liver microsomes suggest that ivermectin does not significantly inhibit the metabolizing activities of CYP3A4, CYP2D6, CYP2C9, CYP1A2, and CYP2E1 [34]. When DP and ivermectin are administered together, however, there may be some competition for CYP3A4. The CYP3A4-inhibitory properties of piperaquine may lead to an increased availability of ivermectin. As ivermectin is not a CYP3A4-inhibitor, the potential increase in the availability of piperaquine due to competition is expected to be low.

We will conduct a placebo-controlled dose-finding study to determine the safety, tolerability, and mosquitocidal effect of 3-day courses of ivermectin when given in combination with a standard 3-day course of DP to identify safe and practical regimens to boost the arsenal of available tools to reduce or interrupt malaria transmission. Pharmacokinetic data will be collected to facilitate the construction of a pharmacokinetic/pharmacodynamic (PK/PD) model to guide future study design.

## Methods

### Design Overview

This is a double-blind, randomized, placebo-controlled, parallel-group, 3-arm, superiority trial to determine the safety, tolerability, and mosquitocidal effect of different doses of ivermectin (ClinicalTrials.gov: NCT02511353). The primary endpoint will be mosquito survival 14 days after a blood feed from a patient who started ivermectin 7 days earlier; 5 days after the last dose of ivermectin with a 3-day regimen administering ivermectin at 0, 24, and 48 hours (days 0, 1 and 2). Because mosquito feeding involves approximately 100 mosquitoes per feed, the study will use a clustered design with the patient as the unit of randomization and the mosquito as the unit of analysis. The study will have a nested rich pharmacokinetic component in the first 36 patients that give additional consent for rich/frequent sampling and a sparse sampling population pharmacokinetic component in the remaining patients. A second nested study will compare the effects of ivermectin when assessed by membrane feeding versus direct skin feeding in all patients who give additional consent for direct skin feeding.

### Primary Objective

The primary objective of the study is to determine the safety, tolerability, and efficacy of ivermectin doses of 0, 300 and 600 mcg/kg/day for 3 days, when provided with a standard 3-day course of the antimalarial DP, on mosquito survival.

### Secondary Objectives

The secondary objectives of the study are (1) to determine the effect of different doses of ivermectin on oocyst development; (2) to determine the pharmacokinetic profile of the different ivermectin regimens; (3) to determine if ivermectin interacts with the pharmacokinetics of piperaquine; (4) to determine whether the addition of ivermectin to DP affects the clinical and parasitological response to DP treatment; (5) to determine the role of genetic variants of CYP3A4 activity in metabolizing ivermectin; and (6) to determine the effect of direct feeding versus membrane feeding on mosquito survival.

### Design Considerations

#### Rationale for Ivermectin Doses of 300 and 600 mcg/kg/day

The goal was to design and evaluate a high-dose ivermectin regimen that could be given daily as adjunct therapy to a 3-day ACT regimen and that builds on the existing safety data available from previous studies. The highest dose of ivermectin used in studies for onchocerciasis is 800 mcg/kg given as a single dose (ie, about 48 mg in an adult male weighing 60 kg). The pharmacokinetic profile of this 800 mcg/kg dose was used to design a 3-day regimen that would achieve a similar maximum drug concentration (Cmax) after the third dose. Since the highest dose of ivermectin used in humans that was tested and found to be well tolerated and safe is 2000 mcg/kg given as a single dose, this provides a large margin of safety allowing for inter-individual variation of pharmacokinetics. The middle group was chosen at 50% of the highest dose to allow for a dose response in terms of tolerance and efficacy.

**Figure 1:**
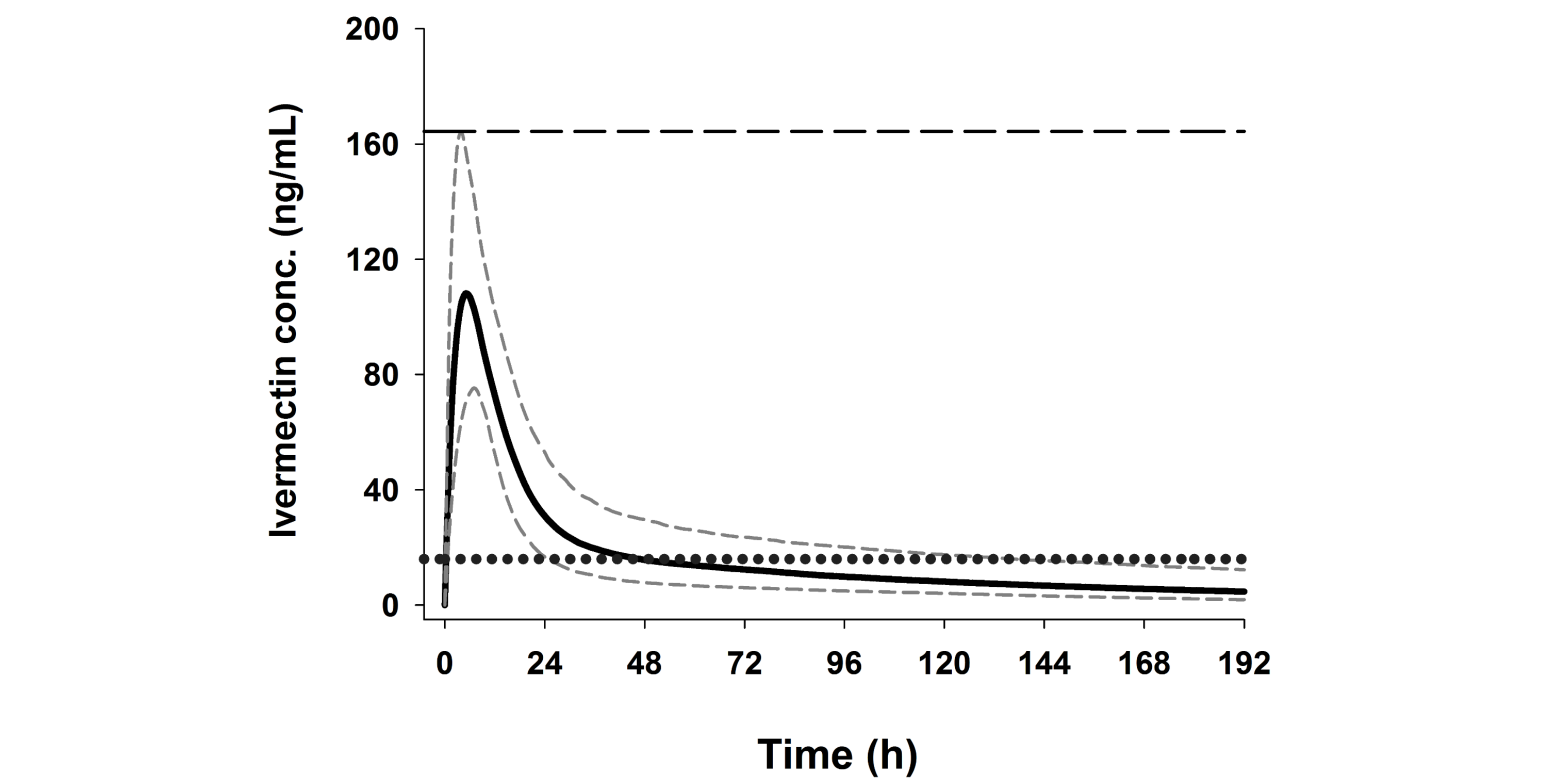
Simulated plasma concentrations of ivermectin 800 mcg/kg single dose. Monte Carlo simulation of 1000 theoretical subjects of ivermectin concentration with 800 mcg/kg single dose (median: solid line, 5th and 95th percentiles: dashed lines). Cmax is 108.1 ng/mL (CI 75.3-164.4). Time above LC50 (16 ng/mL; dotted line) is 1.9 days (CI 1.0-5.7).

**Figure 2:**
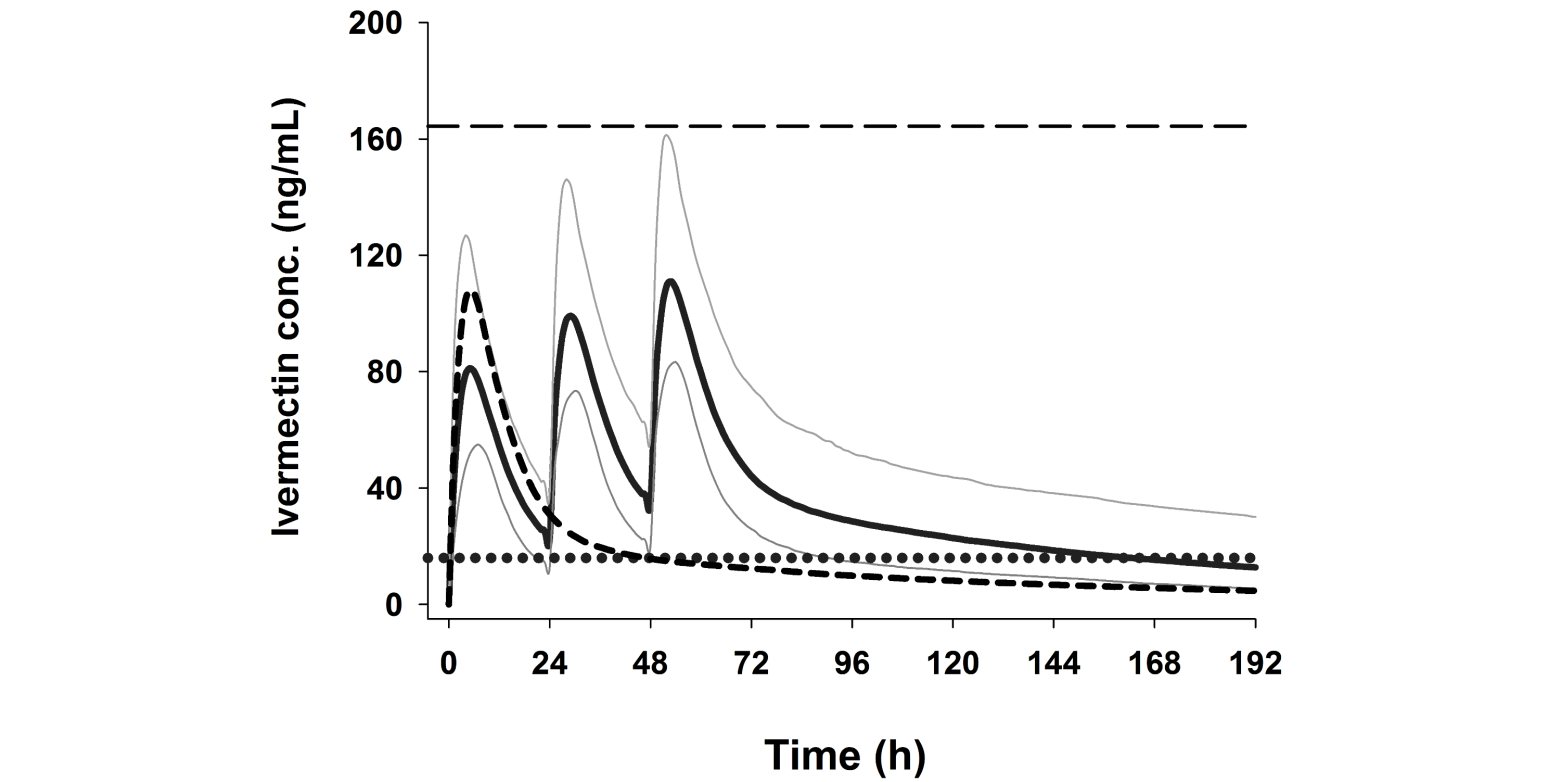
Simulated plasma concentrations of ivermectin 600 mcg/kg/day 3-day regimen and 800 mcg/kg/day single dose. Monte Carlo simulation of 1000 theoretical subjects of ivermectin concentrations following 600 mcg/kg/day for 3 days (median: solid line, 5th and 95th percentiles: grey lines), achieving similar Cmax concentrations compared to 800 mcg/kg single dose (median: dash curve, 95th percentile of Cmax: dashed horizontal line). The median time above LC50 (16 ng/mL; dotted horizontal line) increases from 1.9 days with 800 mcg/kg single dose to 6.8 days with 600 mcg/kg/day for 3 days.

Using existing literature data [24,35] we developed a pharmacokinetic model for ivermectin in humans. Using the parameter estimates from the model, Monte Carlo simulations were performed for 1000 theoretical participants assuming a 30% variability in parameter estimates (CL/F 11.8 L/h, Vc/F 195.0 L, Q 18.9 L/h, Vp 882 L, and Ka 0.24/h). The simulations showed that the Cmax associated with a single dose of 800 mcg/kg was estimated at 108 ng/ml and the 95% percentile as 164 ng/ml (Figure 1). A regimen of 600 mcg/kg/day for 3 days would give a similar Cmax (111 ng/mL) and corresponding 95% percentile (161 ng/mL) as the single dose 800 mcg/kg regimen (Figure 2 and Table 2). A regimen of 300 mcg/kg/day for 3 days would give approximately half those values. The 3-day regimens were predicted to increase the time that ivermectin concentrations remain above the lethal concentration 50% (LC50) of 16 ng/ml [12] from 46 hours with the 800 mcg/kg single dose to 86 and 162 hours, respectively, with the 300 and 600 mcg/kg/day regimens. The 16 ng/mL threshold was chosen as this was the median of 3 LC50 concentrations reported previously [12,14,15]. The simulated data were in excellent agreement with actual data observed in a dose-finding study by Guzzo et al 2002 [24], which indicated proportional pharmacokinetics at doses ranging from 30 to 120 mg, thus giving confidence in the parameters used in the simulations.

**Table 2:** Summary of simulated maximum drug concentration and time above lethal concentration 50%.

Ivermectin dosing regimen	Cmax^a^ (median 5th-95th percentiles)	Days above LC50^b^ (median 5th-95th percentiles)
800 mcg/kg single dose	108.1 (75.3-164.4)	1.9 (1.0-5.7)
600 mcg/kg/day for 3 days	111.0 (83.2-161.2)	6.8 (3.8-13.4)
300 mcg/kg/day for 3 days	55.4 (41.6-80.6)	3.6 (2.8-7.5)

^a^Cmax: maximum drug concentration (ng/mL).

^b^LC50: lethal concentration 50% (16ng/mL).

#### Parallel Versus Dose-Escalation Design

The proposed study uses a standard parallel design, comparing the 2 intervention arms with the placebo arm. This parallel design, instead of a dose-escalation design (when the lower dose group would be studied first prior to enrolling patients in the higher dose group), was considered appropriate because the Cmax levels and the 95th percentile concentrations in the proposed highest dose group of 600 mcg/kg/day will be equivalent to the Cmax found with single dose 800 mcg/kg, which has been administered to at least 402 patients before as treatment for onchocerciasis or as part of regulatory studies (see Table 1). Furthermore, with 30% variation assumed, the Cmax is estimated to remain well below the Cmax value obtained with 2000 mcg/kg, the highest dose tested and which was well-tolerated in a dose escalation study.

#### Why Patients with Malaria?

The study will enroll patients with symptomatic uncomplicated malaria, instead of asymptomatic patients with malaria parasites (carriers) or malaria negative individuals who are the predominant target population in MDA campaigns. However, it is unlikely that the mosquitocidal effect of ivermectin will differ much amongst these groups. Preference is given to symptomatic patients based on the rationale that this study is labor intensive, requiring very frequent patient follow-up and blood sampling, and thus requires a major commitment from study participants. Symptomatic patients, aside from similarly requiring antimalarial treatment, are more likely to favor hospital admission and frequent outpatient visits than asymptomatic patients or other volunteers. The frequent follow-up is potentially also more beneficial to the patients with symptomatic malaria than asymptomatic patients.

#### Justification for Host Genetic Studies

The cytochrome P450s (CYPs) are the major enzymes involved in drug metabolism. To be able to interpret variations in the pharmacokinetic drug profiles of piperaquine and ivermectin, and any drug interactions, we need to determine the genotypes of the genes encoding CYP enzymes (see above).

#### Direct Skin Feeding Versus Membrane Feeding

The primary endpoint is based on membrane feeding of mosquitoes using blood obtained by venepuncture from patients recently treated with ivermectin. However, a nested sub-study, in all those that give additional consent, will compare mosquito mortality rates between clusters fed using standard membrane feeding versus clusters fed directly (by allowing them to feed on the arm of the study participant). Ivermectin feeding studies with direct feeding on humans [13], and cattle [36], have shown a longer mosquitocidal effect (greater than 2 weeks) in comparison with studies using membrane feeding (less than 7 days) [14].

We hypothesize that direct feeding could result in higher mosquito mortality due to potential differences between venous blood (used in membrane feeding) and blood in subdermal venuoles and arterioles (the main source of blood for mosquitoes during direct skin feeding) due to drug accumulation in subcutaneous fat, dermal, and facial tissue (2- to 3-fold higher concentrations than in venous blood [37]), or increased exposure of the mosquito to ivermectin through other means like perspiration.

There have been no studies conducted directly comparing direct feeding versus membrane feeding on mosquito mortality following ivermectin administration. However, previous studies looking at infectivity (ie, the ability of the vector to develop oocysts and sporozoites after ingesting gametocytes) showed significant differences in terms of infectivity in favor of direct feeding (odds ratio 2.39) [38]. Although the mechanisms involved in infectivity studies may differ from studies addressing the killing effect of ivermectin, this recent infectivity study [38] indicates the importance of addressing the potential that the feeding method to expose mosquitoes to ivermectin may be an important effect modifier and that studies using membrane feeding may potentially underestimate the true effect of ivermectin.

Membrane feeding will be used as the primary outcome because direct skin feeding is labor intensive, may be unpleasant to the study participants, and result in higher refusal rates.

### Study Setting

The study will be conducted in the Jaramogi Oginga Odinga Teaching and Referral Hospital (JOOTRH) in Kisumu, western Kenya, a major tertiary care hospital. Almost 25,000 outpatients are treated for clinical malaria at JOOTRH annually, of which one-third are laboratory-confirmed. Approximately 20% of these patients are 18 to 50 years old. Malaria positive individuals will also be pre-screened at 5 nearby health facilities; those that pass pre-screening and give consent will be brought to JOOTRH for screening and all further study procedures.

### Eligibility Criteria

Inclusion and exclusion criteria for the study are shown in Textbox 1.

**Eligibility Criteria**

Inclusion Criteria:

- Symptomatic, uncomplicated *P. falciparum* infection
- Positive malaria microscopy or malaria rapid diagnostic test (RDT, pLDH)
- Age 18 to 50 years
- Provide written informed consent
- Agree to be able to travel to clinic on days 1, 2, 7, 10, 14, 21, and 28

Exclusion Criteria:

- Signs or symptoms of severe malaria
- Unable to provide written informed consent
- Women who are pregnant or breast feeding
- Hypersensitivity to ivermectin or DP
- Rate corrected QT interval (QTc) of greater than 460 ms on electrocardiogram (ECG)
- Body mass index (BMI) below 16 or above 32 kg/m2
- Hemoglobin (Hb) concentration below 9 g/dL
- Taken ivermectin in the last month
- Taken DP in the last 12 weeks
- *Loa loa* as assessed by travel history to Angola, Cameroon, Chad, Central African Republic, Congo, DR Congo, Equatorial Guinea, Ethiopia, Gabon, Nigeria and Sudan
- History and/or symptoms indicating chronic illness
- Current use of tuberculosis or anti-retroviral medication
- Previously enrolled in the same study

### Trial Medications and Interventions

Participants will be randomized to one of the following 3 arms: (1) the “0 mcg/kg” (placebo) arm consisting of DP plus ivermectin-placebo 600 mcg/kg/day for 3 days, (2) the “300 mcg/kg” arm consisting of DP plus ivermectin 300 mcg/kg/day and ivermectin-placebo 300 mcg/kg/day for 3 days, or (3) the “600 mcg/kg” arm consisting of DP plus ivermectin 600 mcg/kg/day for 3 days. Patients will receive their weight-based doses of DP and ivermectin/placebo. Each dose will be given as directly observed therapy by study staff, after which participants will be monitored for 30 minutes for any vomiting and adverse reactions. If vomiting occurs within 30 minutes, then the participant will be withdrawn from the study, DP will be re-administered, and no further ivermectin will be given.

#### Dihydroartemisinin-Piperaquine

DP was selected as the drug of choice as it is the most likely candidate to be used in future MDA campaigns because of the longer prophylactic effect against malaria (4 to 6 weeks) compared with 2 to 3 weeks with artemether-lumefantrine (AL). Each participant will receive a weight-based dose of DP 320/40 mg (Eurartesim, Sigma Tau, Italy) as per the product insert (36 to 75 kg: 3 tablets, greater than 75 kg: 4 tablets) once a day for 3 days.

#### Ivermectin and Placebo

Ivermectin and/or placebo 6 mg tablets (Iver P, Laboratorio Elea, Argentina) will be administered per bodyweight. The 600 mcg/kg/day arm will receive only ivermectin tablets, the 300 mcg/kg/day arm will receive half the number of ivermectin tablets and an equal number of placebo tablets, and the 0 mcg/kg/day arm will receive only placebo tablets. All participants will receive the same total number of tablets once a day for 3 days based on their bodyweight (45 to 55 kg: 5 tablets, 55 to 65 kg: 6 tablets, 65 to 75 kg: 7 tablets, 75 to 85 kg: 8 tablets, 85 to 95 kg: 9 tablets, 95 to 105 kg: 10 tablets).

### Endpoints and Outcome Measures

#### Primary Efficacy Outcome

The primary efficacy outcome is mosquito survival at 14 days after feeding on blood taken from study participants who started the 3-day ivermectin and DP regimen 7 days earlier (Multimedia Appendix 1).

#### Secondary Outcomes

Secondary outcomes include (1) mosquito survival at each day, up to day 21 or 28, after each feeding experiments performed at 0, 2 days plus 4h, 7, 10, 14, 21, and 28 days after start of treatment; (2) occurrence of oocysts from day 10 onwards after each feeding as determined by polymerase chain reaction (PCR); (3) malaria clinical and parasitological treatment response by day 28; and (4) plasma concentration profiles of piperaquine and ivermectin as described by standard pharmacokinetic metrics, for example area under the curve measurements from time zero to infinity (AUC0-∞), time zero to the time of the last measurable concentration (AUC0-tlast), Cmax, and plasma half-life, time to maximum plasma concentration, etc (Multimedia Appendix 1).

#### Tolerability and Safety Endpoints

Tolerability and safety endpoints are shown in Textbox 2.

**Tolerability and safety endpoints.**

Tolerability

- Any adverse events assessed in general toxicity questionnaires asked at each study visit

Safety

- Primary
  Mydriasis quantitated by pupillometry [24]
- Secondary
  - Central nervous system (CNS) effects
  - General toxicity
  - Serious adverse events
  - Hemoglobin concentrations
  - QTc interval (see below “Electrocardiogram Monitoring”)

### Participants’ Timeline

#### Overview Study Phases

The study plan and schedule of assessment is provided in Multimedia Appendix 1. The participant’s timeline will consist of a pre-screening visit (visit 1), consent, screening, and enrolment visit (visit 2), 2 subsequent treatment visits (3 and 4) on days 1 and 2, and 6 follow-up visits for assessment of efficacy parameters (visits 5 to 10). For those enrolled in the pharmacokinetic study additional visits for drug level sample are required as outlined in Multimedia Appendix 2.

#### Visits 1 and 2: Pre-Screening, Consent, Screening and Enrolment

Patients presenting to the outpatient departments of the study clinics will be pre-screened to determine if they meet readily apparent study eligibility criteria including (1) age 18 to 50 years; (2) uncomplicated malaria; (3) in Kisumu next 4 weeks; (4) hemoglobin (Hb) less than or equal to 9g/dL (if already performed); (5) not pregnant or breast feeding; (6) no known chronic illness; and (7) not previously enrolled in IVERMAL. Patients passing pre-screening will be approached to obtain consent. For those consenting, study-specific screening procedures will take place, including demographics, full history, past medication use, travel history (*Loa* endemic countries), physical examination, electrocardiogram (ECG), pupillometry, and laboratory tests (to confirm malaria, Hb, and pregnancy). Those fulfilling all enrolment inclusion criteria and not meeting any exclusion criteria will be enrolled into the study, randomized, and treated with the appropriate tablets according to study arm (Textbox 1). Estimated duration is 1.5 to 2.0 hours.

#### Visits 3 and 4: Treatment Visits

Participants will return to the outpatient clinic on day 1 and 2 for the 2nd and 3rd dose of study drugs. In exceptional cases a participant will be permitted to take the study medication at home or the participant will be visited at home by study staff to administer the medication. A follow-up ECG will be taken just prior to and 4 to 6 hours after the last dose of DP plus ivermectin on day 2.

#### Visits 5 to 10: Scheduled Follow-Up Visits

Participants will return to the outpatient clinic for follow-up as specified (see Multimedia Appendix 1). A questionnaire will assess the presence of signs and symptoms, including any adverse effects. A brief clinical examination will be performed and a venous blood sample will be taken for malaria diagnosis, Hb, and drug levels. On visits 5 (day 2 plus 4h) and 6 (day 7), drug levels will also be determined in a finger prick sample. A final follow-up ECG will be taken on the day 28 visit. Participants will be asked to provide telephone numbers so that study staff may make every effort to follow-up participants who have missed scheduled visits (Multimedia Appendix 2, section 8.5.5, page 31).

#### Unscheduled Visits

At any time, participants displaying signs or symptoms of severe malaria will be admitted to the inpatient ward for further evaluation and treatment free of charge. Blood samples for malaria smears, parasite genetics (filter paper dried blood spots) and Hb will be taken if clinically indicated (e.g. documented fever greater than or equal to 37.5°C axillary, or a history of fever in the last 24 hours).

### Sample Size

The study requires a total of 141 participants (47 participants in the 0, 300, and 600 mcg/kg/day groups each). This is powered at 80% to detect a relative increase of 30% (RR 1.300) in the 14-day post-feeding mortality rate (primary outcome) from 24% in the control group (0 mcg/kg ivermectin) to 31.2% in the 300 mcg/kg/day group, and a 25% (RR 1.246) increase from 31.2% with 300 mcg/kg/day to 38.9% in 600 mcg/kg/day recipients, measured from blood taken 7 days after the start of intake of ivermectin and using clusters of 100 anopheline mosquitoes allowing for 10% non-feeders (alpha=.05). The same sample size would give 90% power to detect a 35% (RR 1.348) increase from 24% (0 mcg/kg/day) to 32.4% (300 mcg/kg/day), and 27.7% increase (RR 1.285) from 32.4% (300 mcg/kg/day) to 41.3% (600 mcg/kg/day). The calculations assume an intracluster correlation coefficient (ICC) of .0622 and allow for 6.5% loss-to follow-up of participants by day 7 (ie, 44 of the 47 patients per arm contribute to the primary analysis) [14]. The 10% non-feeding rate is based on current data from the same laboratories at Kenya Medical Research Institute (KEMRI), Kisian, Kenya. The 24% mortality rate estimate by day 14 post-feeding in the control arm is average of observation at KEMRI (18.3%) and in a recent study in Burkina Faso, which showed a 21.2% mortality by day 10 [14], which when extrapolated with 4 additional days predicted a mortality of 29.7% by day 14. The ICC value of .0622 was calculated using data from the recent study in Burkina Faso (Bousema, personal communications) [14].

### Assignment of Interventions

#### Allocation

The study will use stratified randomization (4 strata) by body mass index (BMI: high/low) and sex (male/female) as these are important determinants of the pharmacokinetics of ivermectin [14]. The high/low BMI thresholds are 23 kg/m^2^ and 21 kg/m^2^, for females and males respectively. Participants will be randomly assigned to 1 of the 3 study arms. The study statistician will computer-generate a randomization sequence using permuted block randomization with fixed block sizes.

#### Blinding

The study will be double-blinded to participants and study staff. Allocation concealment will be achieved by use of sealed opaque envelopes. All study participants in all 3 arms will receive standard dose DP, and also active (600 mcg/kg/day arm), placebo (0 mcg/kg/day arm), or a combination of active and placebo ivermectin tablets (300 mcg/kg/day arm), such that each arm receives the same number of tablets in each weight strata.

### Pharmacokinetic Studies

#### Overview

The first 36 patients to give additional consent for rich pharmacokinetics (approximately 12 per arm), will be enrolled in a rich pharmacokinetic study using frequent sampling per individual (26 samples per patient, see Multimedia Appendix 2 [Table 2, page 15]) to determine the detailed pharmacokinetic profile of the 2 regimens and assess whether any drug interaction occurs with piperaquine that is of clinical relevance. The remaining patients (approximately 35 per arm) will contribute to a population pharmacokinetic study consisting of sparse pharmacokinetic sampling with a maximum of 13 samples per patient including baseline (1 venous sample), 6 scheduled visits as part of the main trial (6 venous and 2 finger prick samples), and 2 extra visits for population pharmacokinetic sampling (2 venous and 2 finger prick samples).

The rich and population pharmacokinetic studies combined will allow us to determine the main sources and correlates of variability in drug concentrations (for both ivermectin and piperaquine), including demographic, pathophysiological, such as BMI and gender, and other factors that might alter dose-concentration relationships. As this is a placebo controlled trial, the sampling methodology for the 47 patients in the ivermectin-placebo arm will be identical to that used for the 300 and 600 mcg/kg arms. The patients in the placebo-ivermectin arm will allow us to determine the piperaquine kinetic profile in the absence of ivermectin.

Finger prick blood draws will be performed at a maximum of 4 time points in addition to the venous blood draws. The aim is to compare the capillary and venous drug concentration levels as it has been hypothesized that these might differ for ivermectin, similar to other drugs including piperaquine. A difference between capillary and venous drug concentrations could help further explain any observed difference in mosquito mortality between membrane and direct skin feeding (see also above “Direct Skin Feeding versus Membrane Feeding”).

#### Standard Pharmacokinetic Study (Rich Sampling)

All of the rich pharmacokinetic participants (approximately 12 per arm) will have venous blood sampled (4 ml whole blood to obtain 2 ml plasma, or 5.2 mL of whole blood if coinciding with a scheduled follow-up visit for the main trial) at baseline and each of 21 follow-up time points listed in Multimedia Appendix 2 (Table 2, page 15). In addition, 4 finger pricks (0.5 mL whole blood) will be taken at days 2 plus 4 h, 3, 4 and 7. The total blood volume to be drawn from these patients is 98.4 mL whole blood over 28 days, 82.8 mL of which is taken during the first 10 days. If more than 2 patients withdraw from the study without giving more than 12 samples, the withdrawing patients will be replaced. Outpatients who consent to the standard pharmacokinetic study will be admitted in the hospital for the first 3 days.

#### Population Pharmacokinetics (Sparse Sampling)

Each of remaining patients (approximately 35 per arm), not enrolled in the rich pharmacokinetic sub-study, contribute to the population pharmacokinetic study, which consists of 13 sampling points (see Multimedia Appendix 2, page 15). Seven of the 13 time points coincide with the timing of the sample for membrane feeding (including the baseline sample), and thus do not require an extra venepuncture (ie, days 0, 2 [52 hrs; 4 hrs after last dose of ivermectin], days 7, 10, 14, 21 and 28). Six of the 13 time points are specific for the population pharmacokinetic study and will require an extra venepuncture (50, 54, 60, 72, 96 and 120 hours, ie, 2, 6, 12, 24, 48, and 120 hrs after the third and last dose of ivermectin). To ensure an equal distribution of samples across the different sampling time points for the extra 2 visits, participants will be divided into 4 extra sampling groups; each of which will contribute 2 extra time points, with the exception of group B which will contribute 1 extra time point (Table 3). In addition, a maximum of 4 finger pricks (0.5 mL whole blood) will be taken at days 2 plus 4 h, 7, and at each of the 2 population pharmacokinetic visits. Thus the total number of samples per participant will be 13 and involve a total of 46.4 mL of whole blood (including the 7 samples for the main trial). The sampling times will be noted in the case record form (CRF), and the patient given a reminder card to return to clinic at their allocated time.

**Table 3:** Schedule of extra sampling points for population pharmacokinetic study by 4 sampling groups.

Subject Group	Sample day^a^ (plus hours after 3rd ivermectin dose)	Sample absolute time^a^, hours	Number per sampling strata
A	2.08 (+2 hours)	50	9
	2.25 (+6 hours)	54	
B	2.25 (+6 hours)	54	8
C	2.50 (+12 hours)	60	9
	3 (+24 hours)	72	
D	4 (+48 hours)	96	9
	5 (+72 hours)	120	
Total			35

^a^Extra visits that need to be made specifically for the population pharmacokinetic samples. The other 7 visits contributing to the population pharmacokinetic analysis (days 0, 2, 7, 10, 14, 21, 28) coincide with the scheduled visits in the main trial. The first day is day=0; day 1 starts 24 hours after the first dose. The allocation to the sampling strata will be at random. However, if a participant indicates he/she is not able to attend a certain follow-up day, the strata can be replaced by another sampling schedule (within the same allocation strata, eg, for BMI, gender, etc) until all 15 or 16 allocations per sampling group have been used.

In anticipation of a 40% refusal rate or loss to follow-up, we estimate that the combined rich and population pharmacokinetic sub-studies will contribute 361 samples including 47 baseline samples (100%) and 314 (60%) follow-up samples out of a potential 524 follow-up samples across 22 sampling time points after baseline, 20 of which overlap, with a total of 12 to 47 observations per time point (see Multimedia Appendix 2,Table 2, page 15).

### Laboratory Procedure

#### Mosquito Colonies

See the “Procedures for Assessing Efficacy and Safety Parameters” section above for use of mosquito colonies and procedures to assess the primary (mosquito survival) and secondary entomological endpoints (sporogony). The section below describes the maintenance of the mosquito colonies.

The mosquito colony used in this study will be *An. gambiae s.s.* Kisumu strain, originally from Kisumu, Kenya. The strain is maintained at the KEMRI/Centre for Global Health Research (CGHR) insectaries and is susceptible to all insecticides approved by the World Health Organization (WHO). When performing membrane feeds on infected human blood, mosquitoes will be kept and fed in cages or paper cups. The cages or paper cups will be kept in a temperature- and humidity- controlled insectary. The feeding and the storage of live infected mosquitoes will occur in sealed rooms with at least 2 doors and/or barriers separating the inner rooms from the outside. Mosquitoes will not be removed from their enclosures, with exception of the cage for oocyst determination. During transportation, live infected mosquitoes will be transported within paper cups that are covered with a moist towel and enclosed within locked cool-boxes to remove any chances of escape. The cool-boxes will only be opened within the confines of a double door insectary.

#### Ivermectin Plasma Concentration

The LC50 has been previously estimated using spiked blood (blood to which known concentrations of ivermectin are added) in membrane feeding assays [12,15]. We will test the concentration of ivermectin in human plasma in order to provide data for a pharmacokinetic/dynamic analysis to obtain estimates of the 10-day LC50 and time post-treatment that the transmission blocking effects (on mosquito survival and oocyst rates) lasts.

#### Hemoglobin Testing

Hb will be tested using HemoCue (Angelholm, Sweden) photometers.

#### Thick and Thin Blood Smears for Malaria

Thick and thin blood films for parasite counts will be obtained and examined. Malaria parasites will be counted against 200 high power fields before a slide is declared negative [39].

#### Processing of Pharmacokinetic Samples

Plasma will be stored locally on site at -20°C or in liquid nitrogen and shipped to a central laboratory for storage at -70°C prior to batch analysis at the Liverpool School of Tropical Medicine. Samples will be shipped in dry ice to the laboratories in Liverpool, United Kingdom where the plasma concentrations of ivermectin and piperaquine will be determined using assays validated to international Food and Drug Administration (FDA) standards. Plasma concentration-time data will be used to evaluate pharmacokinetic parameters including CL/F (oral clearance), V/F (oral volume of distribution), and Ka (absorption rate constant) using population pharmacokinetic methods. Area under the curve (AUC) and half-life will also be calculated.

### Statistical Methods

A study statistical analytical plan for the final analysis, that supersedes the study protocol, has been drawn up during the course of the study before the unblinding of data at database lock (see Multimedia Appendix 3).

### Procedures for Assessing Efficacy and Safety Parameters

#### Membrane Feeding Procedure

The following procedures will be conducted in accordance with a standard membrane feeding protocol [40]. A 1 mL sample of the participant’s blood will be drawn into a sodium heparin (coated) tube pre-heated to 37.5°C. Within 2 minutes the blood will be placed in a glass bell membrane feeding system and cups of mosquitoes will commence feeding. For each feeding 3 new cups (2 cups for mosquito survival, and 1 cup for oocysts) of 50, 3 to 5 day old female, insectary-reared *An. gambiae s.s.* mosquitoes will be presented to the membrane feeder for 20 minutes. The number of mosquitoes with an engorged abdomen will be counted and those with lean abdomens discarded. Each day up to day 28 (mosquito survival cups) or day 10 (oocyst cup), the number of dead mosquitoes will be counted and removed. After the initial feeding on human blood, the mosquitoes will be kept in an incubator and maintained on sugar feeds. Insectary staff assessing mosquito survival and oocyst development will be blinded to all characteristics of the cups (ie, participant identification, study arm, duration between treatment and feeding, and feeding method).

#### Primary Efficacy Outcome

The primary outcome will be the survival of mosquitoes (from the 2 mosquito survival cups) at 14 days after feeding on blood taken from study participants who started the 3-day ivermectin and DP regimen 7 days earlier.

#### Secondary Efficacy Outcomes

Although the primary endpoint is assessed at day 14, the study will collect survival data of mosquitoes at each day up to day 21 or 28 for the mosquito survival cups and day 10 in the case of oocyst cups, after each feeding experiments performed at 0, 2 days plus 4h, 7, 10, 14, 21, 28 days after start of treatment. The methods will be identical to that described for the primary outcome where each day beyond day 14 the number of dead mosquitoes will be counted and removed until day 28 inclusive. The exact number of follow-up days (21 or 28 days) will be subject to logistical constraints of the laboratory, and mortality rates in the mosquito populations which will be further determined prior to the start of the study. The aim is to determine the median time to mortality, which requires that at least half of the mosquito population has died in each arm. It is anticipated that 21 days will be sufficient.

#### Direct Skin Feeding and Mosquito Survival

**Figure 3:**
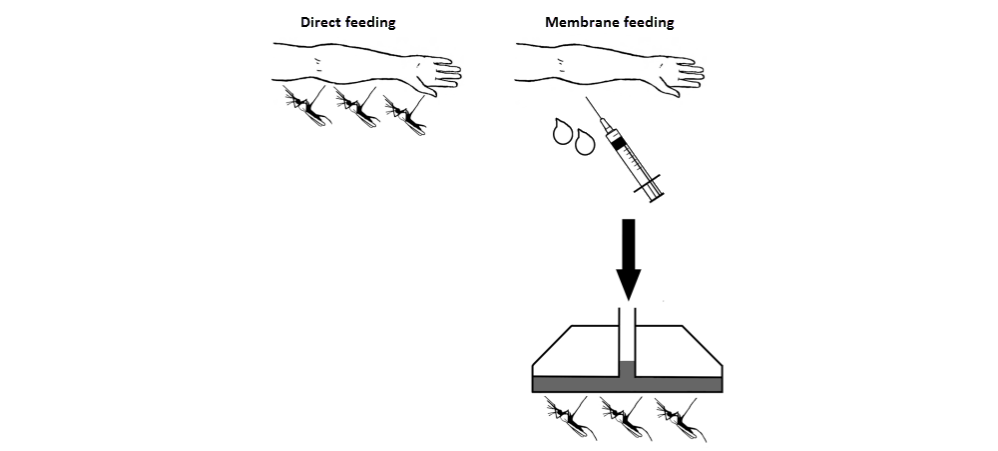
Difference between membrane feeding and direct feeding (adapted from Bousema et al 2012 [38]).

A sub-study will determine the effect of direct feeding versus membrane feeding on mosquito survival, after feeding experiments performed at 7 days after the start of treatment. In direct skin feeding assays, 1 cup of 50 mosquitoes is placed directly on the skin of the human host and allowed to feed for 15 minutes (Figure 3). Further procedures after direct feeding are identical to those after membrane feeding.

#### Infectivity to Mosquitoes (Oocyst Polymerase Chain Reaction)

On day 10 post membrane feeding, when residual DNA from the blood meal is highly unlikely [14,41,42], all surviving mosquitoes in the oocyst cup will be preserved to determine oocyst prevalence by PCR. Mosquitoes will be homogenized and processed, in 2 pooled batches per cup.

#### Asexual Treatment Response and Parasite Clearance

Standard methods will be used to assess the in vivo treatment response to DP using the microscopy and rapid diagnostic test (RDT) data collected at each scheduled follow-up visit and criteria described by World Wide Antimalarial Resistance Network (WWARN) [43].

#### Pupillometry

In animal studies, mydriasis has been shown to be a first sign of ivermectin toxicity. To monitor for possible toxicity, pupil diameter size will be measured at baseline and each scheduled visit using a portable, single-button activation, battery operated hand-held pupillometry device that very accurately measures pupil size requiring no calibration (NeurOptics VIP-200 Variable Pupillometer). This device measures the pupil 30 times per second over a 5-second period and provides the average pupil diameter and standard deviation (+/- 0.1 mm). The measurements will be taken in a dark room with standardized lighting conditions.

#### Electrocardiogram Monitoring

Piperaquine can potentially lead to prolongation of the rate corrected QT interval (QTc) on an electrocardiogram (ECG). To exclude a possible interaction between ivermectin and piperaquine leading to an increased QTc interval, 12-lead ECGs will be performed to measure the QTc interval at baseline, day 2 pre last dose, day 2 at 4 to 6h post last dose and again at day 28. The day 28 sample is included as it can be difficult to assess a true baseline in patients with acute malaria, as malaria and fever are known to increase the heart rate and decrease the QTc interval. On day 28 most, if not all, patients will be malaria free and residual piperaquine levels low enough not to affect QTc intervals. A portable ECG machine (MAC 600, General Electric, US) will be used with automated ECG interpretation. Patients with a QTc value of 480 ms or greater prior to the last dose of DP will not receive the last dose of DP, but receive a full course of artemether lumefantrine instead. Fridericia’s correction will be used to calculate the QTc values for final data analysis using the following equation: QTc = QT/RR^0.33^.

#### Adverse Events

Adverse events and serious adverse events will be monitored, managed, and recorded during the course of the study. They will be recorded and tabulated for each treatment arm, overall, and per body system (see Multimedia Appendix 2, Section 9.6, Safety Monitoring and Reporting).

### Ethics Approval and Consent to Participate

This protocol, the informed consent documents, and patient information sheets have been reviewed and approved by the Research Ethics Committees at KEMRI (protocol #2775), LSTM (protocol #14.002), and JOOTRH. The Centers for Disease Control and Prevention (CDC, protocol #6720) gave approval for reliance on the KEMRI institutional review board (see Multimedia Appendix 4 Ethics Approvals KEMRI, CDC, LSTM, and JOOTRH).

## Results

Recruitment started July 20, 2015. Enrolment was completed May 2016, and clinical follow-up was completed 4 weeks later in June 2016. Mosquito follow-up was completed in July 2016, 4 weeks after completion of the clinical follow-up. Unblinding and analysis will commence once the database has been completed, cleaned, and locked.

## Discussion

### Principal Findings

New strategies for malaria control, and eventually for elimination are critically needed. This study will seek to answer the question as to whether higher doses of ivermectin (300 and 600 mcg/kg/day for 3 days) are well tolerated, safe, and result in longer durations of mosquitocidal effects than standard 150 to 200 mcg/kg single dose treatments. This study requires major infrastructure and collaboration, as it brings together the disciplines of clinical medicine, entomology, parasitology, pharmacokinetics, and pharmacogenetics in a clinical trial. For this study, 141 patients and 150,000 mosquitoes will each be followed for 28 days. For this reason, this trial has been placed at the KEMRI, CDC, and LSTM collaboration in western Kenya, a research site, which in collaboration with its partners, has been conducting research for over 35 years and has the capacity to undertake such a trial. An important possible limitation of this study is that it will enroll participants with symptomatic malaria, whereas possible future applications of high-dose ivermectin may involve MDA with ACT’s targeting asymptomatic carriers and uninfected individuals in addition to symptomatic patients. Should this study show promising results, then the next step will be to evaluate safety, tolerability, and efficacy in younger age groups with the ultimate goal of testing its effect on malaria transmission when applied at the population level through MDA.

### Conclusion

High-dose ivermectin, if found to be safe and well tolerated, could potentially complement existing tools for malaria elimination.

## Appendix

Multimedia Appendix 1 Summary of study design and schedule of assessment (SPIRIT flow diagram).

Multimedia Appendix 2 Full study protocol (including SPIRIT checklist): v4.1, dated 14-Jan-2016.

Multimedia Appendix 3 Statistical Analytical Plan (SAP): v1.0, dated 19-Feb-2016.

Multimedia Appendix 4 Ethics approvals v4.1: KEMRI, CDC, LSTM, and JOOTRH.

## Abbreviations

ACT: artemisinin-based combination therapy
AUC: area under the curve
CDC: Centers for Disease Control and Prevention
Cmax: maximum drug concentration
CYP: cytochrome P450
CYP3A4: cytochrome-P450 3A4
DHA: dihydroartemisinin
DP: dihydroartemisinin-piperaquine
ECG: electrocardiogram
Hb: hemoglobin
ICC: intracluster correlation coefficient
JOOTRH: Jaramogi Oginga Odinga Teaching and Referral Hospital
KEMRI: Kenya Medical Research Institute
LC50: lethal concentration 50%
LLINS: long-lasting insecticide treated nets
LSTM: Liverpool School of Tropical Medicine
MDA: mass drug administration
PCR: polymerase chain reaction
RDT: rapid diagnostic test
QTc: rate corrected QT interval on an electrocardiogram
