# Supplementary material for: Efficacy and Safety of High-Dose Ivermectin for Reducing Malaria Transmission (IVERMAL): Protocol for a Double-Blind, Randomized, Placebo-Controlled, Dose-Finding Trial in Western Kenya
Source: JMIR Res Protoc. 2016 Nov 17;5(4):e213. doi: 10.2196/resprot.6617 (PMC5133431; doi:10.2196/resprot.6617)
Supplement: Multimedia Appendix 1 [file resprot_v5i4e213_app1.pdf]

*Multimedia Appendix 1: Summary of Study Design and Schedule of Assessment (SPIRIT Flow Diagram)*

| Phase | Recruitment Phase | Enrolment | Treatment Phase | | | Post-treatment Follow-up phase | | | | | |
|---|---|---|---|---|---|---|---|---|---|---|---|
| Location | OPD | OPD | OPD | OPD[b] | OPD[b] | OPD visits | | | | | |
| Visit number | #1 | #2 | #2 | #3 | #4 | #5 | #6 | #7 | #8 | #9 | #10 |
| Study Time<br>Hour | -1h [a] | -0.5h | 0h | 24h | 48h | 52h | 168h | 240h | 336h | 504h | 672h |
| Day | D00 | D00 | D00 | D01 | D02 | D02+4h ±2h[c] | D07 ±3d[c] | D10 ±3d[c] | D14 ±3d[c] | D21 ±3d[c] | D28 ±3d[c] |
| Recruitment | | | | | | | | | | | |
| Pre-screening | X | | | | | | | | | | |
| Enrolment | | | | | | | | | | | |
| Eligibility screen | | X | | | | | | | | | |
| Informed Consent | | X | | | | | | | | | |
| Study code issued | | X | | | | | | | | | |
| Allocation | | X | | | | | | | | | |
| Interventions | | | | | | | | | | | |
| IVM-0 arm | | | X[i] | X[i] | X[i] | | | | | | |
| IVM-300 arm | | | X[i] | X[i] | X[i] | | | | | | |
| IVM-600 arm | | | X[i] | X[i] | X[i] | | | | | | |
| Clinical assessments | | D00 | | | D02 | D02+4h | D07 | D10 | D14 | D21 | D28 |
| Copy Clinic/Lab data from hospital records | | | | | | | | | | | |
| Physical Exam. | | X | | | | X | X | X | X | X | X |
| Pupillometry | | X | | | | X | X | X | X | X | X |
| ECG | | X | | | X[j] | X | | | | | X |
| Questionnaire AE | | X | | | | X | X | X | X | X | X |
| Blood sample[d,e,h] | | V 5.4ml | | | | V+C 5.9ml | V+C 5.9ml | V 5.4ml | V 5.4ml | V 5.4ml | V 5.4ml |
| Unscheduled sick-patient clinic visits | | | | | | Passive surveillance for 28 days (clinical malaria and other acute illnesses)[k] | | | | | |
| Entomological assessments | | D00 | | | | D02+4h | D07 | D10 | D14 | D21 | D28 |
| Membrane feeding[f] | | X | | | | X | X | X | X | X | X |
| Direct feeding | | | | | | | X[g] | | | | |

Visit 1: Pre-Screening interview
Visit 2: Consent, Screening, & Enrolment. First treatment dose given under direct observation.
Visits 3 and 4: Treatment visits. 2nd and 3rd treatment doses given under direct observation. In exceptional cases doses of day 1 and 2 can be taken at home.
Visits 5 to 10: Scheduled follow-up visits for assessment of efficacy parameters

a. Patients can be pre-study screened any time from visiting the OPD. The figure of -1 hour is provided for illustration purposes only.
b. The day of enrolment is always considered as Day-0. Doses given under direct observation. In exceptional cases doses of day 1 and 2 can be taken at home.
c. Visit window= number of days an actual subject visit may fall outside of the planned protocol schedule visit to still meet protocol requirements. It is preferential to stick to the scheduled days of visit. However, if this is not feasible (e.g. due to other commitments of the patient) then it preferable to allow flexibility in the schedule. The date of actual visit will always need to be recorded in the CRF.
d. Enrolment & baseline blood sample (~5.4 mL) by venepuncture: haemoglobin (0.01 mL), malaria smear / RDT (0.01 mL), dried blood spots (DBS) for PCR (0.3 mL) will be stored for parasite genetics to differentiate reinfection from recrudescence in case of treatment failure, membrane feeding (~1 mL), pharmacology (~4 mL: ~2mL plasma for drug levels, 2mL pellet for host metabolism genetics).
e. Follow-up blood sample (~5.4 mL) by venepuncture: haemoglobin (0.01 mL), malaria smear (0.01 mL), malaria RDT (0.005 mL), dried blood spots for PCR (0.3 mL), membrane feeding (~1 mL), pharmacology (~4mL: ~2mL plasma for drug levels).
f. Membrane feeding will be used to assess: Mosquito survival (daily up to 21 to 28 days after feed; Oocyst prevalence at day 10 after feeding).
g. Direct skin feeding in a sub-sample only.
h. Finger prick (capillary) blood sample on Day 2+4h and Day 7: drug levels (~0.5ml to obtain ~0.25ml plasma).
i. Each treatment visit: IVM-0 (DP, placebo 600 mcg/kg), IVM-300 (DP, ivermectin 300 mcg/kg/day, placebo 300 mcg/kg/day), IVM-600 (DP, ivermectin 600 mcg/kg/day)
j. Before 3rd dose.
k. RDT/smear, Hb, dried blood spots for parasite genetics.

V=venepuncture. C=capillary, DP=dihydroartemisinin-piperaquine, IVM=ivermectin, Hb=haemoglobin, MS=malaria smear, Pf=Plasmodium falciparum
