# Supplementary material for: Efficacy and Safety of High-Dose Ivermectin for Reducing Malaria Transmission (IVERMAL): Protocol for a Double-Blind, Randomized, Placebo-Controlled, Dose-Finding Trial in Western Kenya
Source: JMIR Res Protoc. 2016 Nov 17;5(4):e213. doi: 10.2196/resprot.6617 (PMC5133431; doi:10.2196/resprot.6617)
Supplement: Multimedia Appendix 2 [file resprot_v5i4e213_app2.pdf]

Efficacy and safety of high-dose ivermectin for reducing malaria transmission: A dose finding study

Short Title: IVERMAL study

Trial Identifiers:				
KEMRI: #2775	LSTM REC: #14.002	CDC IRB: #6720	KPPB Kenya: ECCT/15/02/03	Clinicaltrials.gov: NCT02511353

Sponsor:

Liverpool School of Tropical Medicine (LSTM) Pembroke Place, Liverpool L3 5QA, UK
Phone: +44 0151 7053212; Email: S.Roberts@liverpool.ac.uk

Principal Investigators:

- Menno Smit, MD, KEMRI & LSTM, Address: KEMRI, P.O. Box 1578, Kisumu 40100, Mobile: +254 703 991 513; E-mail: menno.smit@liverpool.ac.uk
- Eric Ochomo, PhD, KEMRI and CDC Collaboration, KEMRI, P.O. Box 1578, Kisumu 40100, Mobile: + 254 723 845 457; E-mail: EOchomo@kemricdc.org

Co-investigators:

See page 7

Funder: Malaria Eradication Scientific Alliance (MESA)

Revision chronology:

Date	Protocol Version	Details of Changes	Authors (see page 7)	Signature Chief Investigator: Prof Feiko ter Kuile
27 Jan 2015	1.4	Original	MS, EO, SW, GA, DW, FtK	
27 Jul 2015	2.0	Addition of 2 additional sites	MS, EO, SW, GA, DW, FtK	
11 Sep 2015	3.0	Addition of 4 capillary blood draws	MS, EO, SW, GA, DW, FtK	
13 Nov 2015	4.0	Addition of 4 additional sites	MS, EO, SW, GA, DW, FtK	
14 Jan 2016	4.1	Clarification of the sampling strategy of additional sites	MS, EO, SW, GA, DW, FtK	

Confidentiality Statement: This document contains confidential information that must not be disclosed to anyone other than the sponsor, the investigator team, host institution, relevant ethics committee and regulatory authorities

1. Title of research protocol 7
2. Investigators and institutions 7
2.1. Principal Investigators 7
2.2. Co-investigators 7
2.3. Non-engaged collaborators 7
2.4. Institutions 7
3. Protocol summaries 8
3.1. Trial Registration data 8
3.2. Narrative Protocol summary 11
4. Introduction 16
4.1. Malaria transmission in western Kenya 16
4.2. Mass Drug Administration for malaria 16
4.3. Ivermectin as adjunct therapy for malaria control 17
4.4. Efficacy against malaria vectors 17
4.5. Modelling: Potential impact of ivermectin 18
4.6. Safety of ivermectin in humans 19
4.7. Interaction: DP and Ivermectin 20
5. Justification for the study 21
5.1. Why is this study needed now? 21
5.2. Other relevant research ongoing elsewhere 21
6. Hypothesis 22
7. Aim & Objectives 22
7.1. Primary objective 22
7.2. Secondary objectives 22
8. Design & Methodology 22
8.1. Overview of design 22
8.2. Design Considerations 23
8.2.1. Why patients with malaria? 23
8.2.2. Ivermectin dose considerations 23
8.2.3. Parallel versus dose-escalation design 25
8.2.4. Justification for host genetic studies 25
8.2.5. Direct skin feeding vs membrane feeding 25
8.3. Study setting 26
8.3.1. Study area 26
8.3.2. Clinical setting 26
8.4. Eligibility criteria 28
8.4.1. Inclusion criteria 28
8.4.2. Exclusion criteria 28
8.5. Interventions 28
8.5.1. Trial Medications and interventions 28
8.5.2. Procedures for Drug handling & Accountability 29
8.5.3. Removal of Patients from Therapy or Assessment 30
8.5.4. Discontinuation from storage of blood for future studies 31
8.5.5. Adherence to study intervention protocol and strategies for retention 31
8.5.6. Prior and concomitant therapy 32
8.6. Endpoints / Outcome measures 32
8.6.1. Primary efficacy outcome (see table Table 1): 32
8.6.2. Secondary outcomes (see table 3): 33

8.6.3. Tolerability and Safety endpoints 33
8.7. Participants timeline 33
8.7.1. Overview Study Phases 33
8.7.2. Visit 1: Pre-Screening interview 33
8.7.3. Visit 2: Screening, Consent & Enrolment 34
8.7.4. Visits 3 and 4: Treatment visits 35
8.7.5. Visits 5 to 10: Scheduled follow-up visits 35
8.7.6. Unscheduled visits 35
8.8. Sample Size 35
8.8.1. Main trial 35
8.8.2. Nested rich pharmacokinetic study 36
8.8.3. Nested direct feeding pilot study 36
8.8.4. ECG monitoring 36
8.9. Recruitment strategies for achieving target sample size 36
8.10. Assignment of interventions 37
8.10.1. Allocation 37
8.10.2. Blinding 37
8.11. Pharmacokinetic (PK) studies 37
8.11.1. Overview 37
8.11.2. Standard pharmacokinetic study (rich sampling) 38
8.11.3. Population pharmacokinetics (sparse sampling) 39

9. Data collection, management and analysis 40
9.1. Laboratory Procedure 40
9.1.1. Mosquito colonies 40
9.1.2. Ivermectin plasma concentration 41
9.1.3. Hemoglobin testing 41
9.1.4. Thick and thin blood smears for malaria 41
9.1.5. Sero-diagnosis for Strongyloides stercoralis 41
9.1.6. Processing of pharmacokinetic samples 41
9.2. Data collection methods & storage 42
9.3. Statistical methods 42
9.3.1. Trial profile and flowchart 42
9.3.2. Baseline characteristics 42
9.3.3. Analysis Populations 42
9.3.4. Missing Data 42
9.3.5. Assessment of efficacy 43
9.3.6. Analysis of adverse events 43
9.4. Procedures for Assessing Efficacy and Safety Parameters 43
9.4.1. Primary efficacy outcome 43
9.4.2. Secondary efficacy outcomes 44
9.4.3. Safety outcomes 45
9.5. Monitoring 45
9.5.1. Data Monitoring 45
9.6. Safety Monitoring and reporting 46
9.6.1. Definitions 46
9.6.2. Identifying, managing adverse events 47
9.6.3. Assessment of Causality 47
9.6.4. Reporting adverse event procedures 48
9.7. Quality assurance 50
9.7.1. Clinical monitoring and auditing 50
9.7.2. Clinical Monitoring 50

9.7.3. Training 50
9.7.4. Quality assurance/control of laboratory tests 51

10. Timeframe and duration of the study 51

11. Ethical Considerations & Regulatory Approvals 51
11.1. Declaration of Helsinki 51
11.2. Regulatory Approval and Trial Authorisation 51
11.3. Research Ethics approval 51
11.3.1. Review Process 51
11.3.2. Protocol amendments 52
11.4. Informed Consent procedures 52
11.4.1. Consent procedures 52
11.4.2. Consent forms 52
11.5. Protection of Privacy and confidentiality 53
11.5.1. Privacy 53
11.5.2. Privacy of individual 53
11.5.3. Confidentiality of data 53
11.6. Declaration of interest 53
11.7. Access to Source Data/Documents 53
11.8. Risks and benefits 53
11.8.1. Risks to Study Participants 53
11.8.2. Benefits to study participants 55
11.9. Ancillary and post-trial care 55
11.9.1. Health care during the trial 55
11.9.2. Trial insurance 55
11.9.3. Post-trial care 55
11.10. Expenses reimbursement and incentives 55

12. Dissemination and application of the results 56
12.1. Result dissemination and publication policy 56
12.2. Impact 57
12.3. Training, Fellowships and Capacity Building 57
12.4. Authorship and publications 57
12.5. Data Sharing Statement 57

13. References 58

14. Financial aspects and conflict of interest 62
14.1. Funding of the trial 62
14.2. Provision of the study drugs 62

15. Budget & Budget Justification 62

16. Appendices 63
16.1. Appendix I. Role Investigators 63
16.1.1. Protocol development: authors' contributions 63
16.1.2. Role Investigators 63
16.1.3. Role Non-engaged Collaborators 64
16.2. Appendix II. Terms of Reference Oversight committees 65
16.2.1. Trial Management Group (TMG) 65
16.2.2. Trial Steering Committee (TSC) 65
16.2.3. Data Monitoring and Ethics Committee (DMEC) 67
16.3. Appendix III. Declaration of Helsinki 68
16.4. Appendix IV. Budget and budget justification 71

16.4.1. Budget 71
16.4.2. Budget Justification 71
16.5. Appendix V. SPIRIT 2013 Checklist clinical trial protocol 72
16.6. Appendix VI. Product characteristics 77
16.7. Appendix VII. Participant information sheets and informed consent forms 84
16.7.1. Informed Consent Participant Information Sheet: Main trial (English) 84
16.7.2. Consent Statement: Main trial (English) 87
16.7.3. Informed Consent Participant Information Sheet: Rich PK study (English) 89
16.7.4. Consent Statement: Rich PK study (English) 92
16.7.5. Informed Consent Participant Information Sheet: Direct feeding (English) 93
16.7.6. Consent Statement: Direct feeding (English) 95
16.7.7. Informed Consent Participant Information Sheet: Main trial (Kiswahili) 96
16.7.8. Consent Statement: Main trial (Kiswhili) 99
16.7.9. Informed Consent Participant Information Sheet: Rich PK study (Kiswahili) 101
16.7.10. Consent Statement: Rich PK study (Kiswahili) 104
16.7.11. Informed Consent Participant Information Sheet: Direct feeding (Kiswahili) 105
16.7.12. Consent Statement: Direct feeding (Kiswhili) 108
16.7.13. Informed Consent Participant Information Sheet: Main trial (Dholuo) 109
16.7.14. Consent Statement: Main trial (DhoLuo) 112
16.7.15. Informed Consent Participant Information Sheet: Rich PK study (Dholuo) 114
16.7.16. Consent Statement: Rich PK study (Dholuo) 117
16.7.17. Informed Consent Participant Information Sheet: Direct feeding (Dholuo) 118
16.7.18. Consent Statement: Direct feeding (DhoLuo) 120
16.8. Appendix VIII. Questionnaires 121

ABBREVIATIONS

95% CI	95 percent Confidence Interval
ACT	Artemisinin-based combination therapy
AE	Adverse event
AIDS	Acquired Immunodeficiency Syndrome
AL	Artemether-Lumefantrine
AUC	Area Under the Curve
CDC	Centers for Disease Control and Prevention
CHW	Community Health Worker
CHEW	Community Health Extension Worker
Cmax	Maximum drug concentration
CRF	Case Record Form
CRO	Contract Research Organization
DHA	Dihydroartemisinin
DP	Dihydroartemisinin-piperaquine
DSMB	Data safety monitoring Board
DMEC	Data Monitoring and Ethics Committee
ERC	Ethics Research Committee
FDA	Food and Drug Administration
GCP	Good Clinical Practice
GMP	Good Manufacturer Practice
Hb	Haemoglobin
HIV	Human Immunodeficiency Virus
IRB	Institutional Review Board
ITNs	Insecticide Treated Nets
ITT	Intention to Treat
IVM	Ivermectin
KEMRI	Kenya Medical Research Institute
LLINS	Long-lasting Insecticide Treated Nets
LSTM	Liverpool School of Tropical Medicine
MDA	Mass drug administration
MIC	Minimum Inhibitory/Insecticidal Concentration
MoH	Ministry of Health
MPAC	Malaria Policy Advisory Committee
MRC	Medical Research Council, UK
NMCP	National Malaria Control Program
PCR	Polymerase Chain Reaction
PQ	Primaquine
RCT	Randomized Controlled Trial
RDT	Rapid diagnostic test
REC	Research Ethics Committee
SAE	Serious adverse event
SOP	Standard Operating Procedure
T1/2	plasma half-life
QTc	QT corrected time interval between Q and T on electrocardiogram
QTcF	QT corrected time interval using Fridericia's correction on ECG
Tmax	time to maximum plasma concentration
TSC	Trial Steering Committee
WHO	World Health Organization

1. TITLE OF RESEARCH PROTOCOL

Efficacy and safety of high-dose ivermectin for reducing malaria transmission: A dose finding study

Short title: IVERMAL study

2. INVESTIGATORS AND INSTITUTIONS

2.1. PRINCIPAL INVESTIGATORS

Dr Menno Smit, MD, MPH (KEMRI, LSTM)
Dr Eric Ochomo, PhD (KEMRI/CDC)

2.2. CO-INVESTIGATORS

Dr Titus Kwambai, MD, MSc (MOH, KEMRI/CDC, LSTM) [TK]
Dr Simon Kariuki, PhD (KEMRI/CDC) [SK]
Dr Nabie Bayoh, PhD (KEMRI/CDC) [NB]
Dr Meghna Desai, PhD, MPH (KEMRI/CDC, CDC-Kenya) [MD]
Dr John Gimnig, PhD (CDC) [JG]
Dr Penelope Phillips-Howard, PhD (KEMRI, LSTM) [PPH]
Dr Aaron Samuels, MD, MHS (CDC) [AS]
Mr Ben Abong'o, MS (KEMRI/CDC) [BA]
Prof Feiko Ter Kuile, MD, PhD (KEMRI, LSTM), [FtK]

2.3. NON-ENGAGED COLLABORATORS

Prof Steve Ward, PhD (LSTM) [SW]
Dr Ghaith Aljayyoussi, PhD (LSTM) [GA]
Prof Duolao Wang, PhD (LSTM) [DW]

2.4. INSTITUTIONS

1. KEMRI- Kenya Medical Research Institute and CDC, Kisumu, Kenya
2. CDC- Centers for Disease Control and Prevention, Atlanta, Georgia, USA
3. MOH- Ministry of Health, Kenya
4. LSTM- Liverpool School of Tropical Medicine

3. PROTOCOL SUMMARIES

3.1. TRIAL REGISTRATION DATA

Data Category	Information			
Primary registry and trial identifying number	ClinicalTrials.gov [NCT02511353]			
Date of registration in primary registry	15 Jul 2015			
Secondary identifying numbers	KEMRI: #2775	LSTM: #14.002	CDC: #6720	KPPB Kenya: ECCT/15/02/03
Source(s) of monetary or material support	Malaria Eradication Scientific Alliance (MESA)			
Primary sponsor	Liverpool School of Tropical Medicine (LSTM)			
Secondary sponsor(s)	NA			
Contact for public queries	Prof Feiko ter Kuile, E-mail: terkuile@lstmed.ac.uk			
Contact for scientific queries	Prof Feiko ter Kuile, LSTM, Pembroke Place, Liverpool, L3 5QA, UK, E-mail: terkuile@lstmed.ac.uk			
Public title	Efficacy and safety of high-dose ivermectin for reducing malaria transmission: A dose finding study			
Scientific title	Efficacy and safety of high-dose ivermectin for reducing malaria transmission: A dose finding study			
Countries of recruitment	Kenya			
Health condition(s) or problem(s) studied	Malaria			
Intervention(s)	Dihydroartemisinin-piperaquine (3-day treatment course) + Ivermectin			
	Placebo comparator (matching tablets containing no active ingredients)			
Study type	Interventional			
	Allocation: randomised; intervention model: parallel assignment; arms:3; allocation ratio: 1:1:1; stratified by body mass index, gender and site. Masking: double blind placebo controlled			
	Primary purpose: prevention			
	Phase-III			
Date of first enrolment	20 Jul 2015			
Target sample size	141 participants (47 per arm)			
Recruitment status	Currently recruiting			

Category	Information
Primary Objective	To determine the safety and efficacy of ivermectin 0, 300, 600 mcg/kg/day for 3 days, when provided with a standard 3-day course of dihydroartemisinin-piperaquine (DP) for uncomplicated malaria, on 14-day mosquito survival after feeding experiment performed at 7 days after treatment
Key inclusion criteria	• Symptomatic, uncomplicated *Plasmodium falciparum* infection<br>• Positive malaria microscopy or malaria RDT (pLDH)<br>• Age: 18-50 years<br>• Provide written informed consent<br>• Agree to be able to travel to clinic on days: 1, 2, 7, 10, 14, 21, and 28
Exclusion criteria	• Signs or symptoms of severe malaria<br>• Unable to provide written informed consent<br>• For women: pregnancy or lactation<br>• Hypersensitivity to ivermectin or DP<br>• Qtc > 460 ms on ECG<br>• Body Mass Index (BMI) below 16 or above 32 kg/m2<br>• Haemoglobin concentration below 9 g/dL<br>• Taken ivermectin in the last month<br>• Taken dihydroartemisinin-piperaquine in the last 12 weeks<br>• *Loa loa* as assessed by travel history to Angola, Cameroon, Chad, Central African Republic, Congo, DR Congo, Equatorial Guinea, Ethiopia, Gabon, Nigeria and Sudan<br>• History and/or symptoms indicating chronic illness<br>• Current use of tuberculosis or anti-retroviral medication<br>• Previously enrolled in the same study

Category	Information
Primary outcome(s)	Mosquito survival: Survival of mosquitoes at 14 days after feeding on blood taking from study participants who started the 3-day ivermectin and DP regimen 7 days earlier
Key secondary outcomes<br><br>Secondary efficacy outcomes	• Mosquito survival: Survival of mosquitoes at each day up to day 21 or 28 after each feeding experiments performed at 0, 2 day+4h, 10, 14, 21, 28 days after start of treatment<br>• Oocyst and sporozoite prevalence: Occurrence of oocysts and/or sporozoites from day 10 onwards after each feeding as determined by PCR or Elisa (using pool testing)<br>• Malaria clinical and parasitological treatment response by day 28<br>• Plasma concentration profiles of piperaquine and ivermectin as described by standard pharmacokinetic metrics (e.g. $AUC0\text{-}_{\infty}$, AUC0tlast, Cmax, $t_{½}$, tmax, etc).<br>• Ectoparasitic infection: bedbugs, head lice, scabies, etc as assessed by pre- and post-treatment questionnaire and physical examination
Safety outcomes	• Primary: Mydriasis quantitated by pupillometry [39]<br>• Secondary:<br>○ CNS effects<br>○ General toxicity<br>○ Serious adverse events<br>○ Haemoglobin concentrations<br>○ QTc interval

3.2. NARRATIVE PROTOCOL SUMMARY

Title: Efficacy and safety of high-dose ivermectin for reducing malaria transmission: A dose finding study

Short Title: IVERMAL study

Background and rationale: In western Kenya the prevalence of malaria in <5 year olds has fallen from 70% in 1997 to 40% in 2008, where it has now stagnated. Innovative approaches are needed to continue towards elimination. Ivermectin is a broad spectrum antiparasitic endectocide widely used for the control of onchocerciasis and lymphatic filariasis at a dose of 150-200 mcg/kg. Ivermectin at this dose has a potent, but short-lived effect for 6-11 days on mosquito survival, egg-laying, and parasite sporogony. Higher doses are needed to prolong its mosquitocidal effects. Regulatory studies have shown ivermectin is very well tolerated and safe even up to 2,000 mcg/kg. We will conduct dose finding studies to evaluate the transmission blocking effect of high-dose ivermectin to define the optimal dose for future use of ivermectin in combination with artemisinin-based combination therapy (ACT) for mass drug administration (MDA) for malaria in Kenya. This study explores a research question of global relevance. A prolonged transmission blocking effect of ivermectin could have substantial consequences for malaria control in the next decades. We expect the results to inform the national regulator in Kenya, national malaria control programs in malaria endemic countries, to inform WHO guidelines, and to contribute to the regulatory process.

Primary objective: To determine the safety and efficacy of ivermectin 0, 300, 600 mcg/kg/day for 3 days, when provided with a standard 3-day course of dihydroartemisinin-piperaquine (DP) for uncomplicated malaria, on 14-day mosquito survival after feeding experiment performed at 7 days after treatment.

Hypothesis: High dose ivermectin at 600 mcg/kg daily for 3 days is well tolerated and safe and will have a longer lasting mosquitocidal effect and a greater potential impact on malaria transmission.

Overview Study Design: Double-blind placebo-controlled, parallel-group, 3-arm, superiority trial to determine the effect of different doses of ivermectin on mosquito survival. The unit of randomization will be the patient, and the unit of analysis will be the mosquito. The primary endpoint will be mosquito survival 14 days after a feed on blood from a patient who has started ivermectin 7 days earlier (i.e. about 5 days after the last dose of ivermectin with a 3-day regimen administering ivermectin at 0, 24, and 48 hours [days 0, 1 and 2]). Because mosquito feeding involves approximately 100 mosquitoes per feed, the study will use a cluster randomized design with the patient as the cluster unit. The study will have a nested rich pharmacokinetic component in the first 12 patients per study arm, and a sparse sampling population pharmacokinetic component in the remaining patients. The study will also compare the potential modifying effect of the method of feeding by comparing the mosquitocidal effect of ivermectin with membrane versus direct skin feeding.

Sites: The study will be conducted in the Provincial Hospital in Kisumu, western Kenya recently renamed to the Jaramogi Oginga Odinga Teaching and Referral Hospital (JOOTRH). Dependent on the recruitment rate, enrolment will be expanded to other health facilities, based on a list of government or private (e.g. mission) health facilities in Kisumu and Siaya counties.

Study Population: Adults with uncomplicated falciparum malaria attending out-patient clinics.

Study Interventions: 1. Dihydroartemisinin-piperaquine (DP) alone, DP plus ivermectin 0 mcg/kg/day for 3 days ("placebo" arm); 2. DP plus ivermectin 300 mcg/kg/day plus 300 mcg/kg/day ivermectin-placebo for 3 days ("300 mcg/kg" arm); 3. DP plus ivermectin 600 mcg/kg/day ("600 mcg/kg" arm).

Outcome Measures: Primary efficacy outcome: Mosquito survival: Survival of mosquitoes at 14 days after feeding on blood taking from study participants who started the 3-day ivermectin and DP regimen 7 days earlier. Primary safety outcome measure: Mydriasis quantitated by pupillometry. Key secondary safety outcome: QTc(F) prolongation by ECG.

Follow-up procedures: Treatment visits on day 1 and 2. Post-treatment follow-up at day 2 (+4 hours after last dose of ivermectin), days 7, 10, 14, 21, and 28.

Sample size: Main trial: 141 participants (47 per arm); Rich pharmacokinetic study: 36 participants (12 per arm); direct feeding vs membrane feeding 27 participants (9 per arm, day 7 post ivermectin only).

Data Analysis: Primary outcomes will be summarised by number (%) of events and analysed using a Generalized Estimating Equations (GEE) model with binomial distribution and log link function that includes treatment as a single predictor taking the cluster design into account, which will generate risk ratios together with their 95% confidence intervals of having a primary endpoint between two active dose groups and the placebo group. Exchangeable covariance structure will be used. The primary endpoint analysis will be based on the ITT population.

Partner institutions: KEMRI and CDC Collaboration, Kisumu, western Kenya; Kenya Ministry of Health, Liverpool School of Tropical Medicine; US Centers for Disease Control and Prevention

Funding: Malaria Eradication Scientific Alliance (MESA) which is funded through a grant from the Bill and Melinda Gates Foundation.

Table 1: Summary Table of Study Design and Schedule of Assessment

Phase	Recruitment Phase OPD	Enrolment OPD	Treatment Phase OPD and home			Post treatment Follow-up phase OPD visits					
Visit number	#1	#2	#2	#3	#4	#5	#6	#7	#8	#9	#10
Visit description	Screening	Enrolment & baseline	DP + IVM treatment visits			Days and hours since enrolment					
Study Time Hour	-1 hour[a]	- 30 min	0 hrs	24hrs	48 hrs	52 hrs	168 hrs	240 hrs	336 hrs	504 hrs	672 hrs
Day	Day 0 (OPD)	Day 0 (OPD)	Day 0 (OPD)	Day 1 OPD/Home	Day 2 OPD/Home	Day 2+4h +/- 2 hrs[c]	Day 7 +/- 3 days[c]	Day 10 +/- 3 days[c]	Day 14 +/- 3 days[c]	Day 21 +/- 3 days[c]	Day 28 +/- 3 days[c]
**Recruitment**											
Pre-screening eligibility	X										
Prior consent discussion	X										
**Enrolment**											
Eligibility screen		X									
Informed Consent		X									
Study code issued		X									
Allocation		X									
**Interventions**											
IVM-Placebo arm			DP1+ Plac1 600 [b]	DP2+ Plac2 600 [b]	DP3+ Plac3 600 [b]						
IVM-300 mcg/kg/day			DP+ Plac1 300 + IVM1 300[b]	DP+ Plac2 300 + IVM2 300[b]	DP+ Plac3 300 + IVM3 300[b]						
IVM-600 mcg/kg/day			DP1+ IVM1 600 [b]	DP2+ IVM2 600 [b]	DP3+ IVM3 600 [b]						
**Assessments** Clinical measures						Day 2+4h	Day 7	Day 10	Day 14	Day 21	Day 28
Copy Clinic/Lab data from hospital records		X									
Physical Exam.		X				X	X	X	X	X	X
Pupillometry		X				X	X	X	X	X	X
ECG		X			X (before DP3)	X					
Questionnaire AE		X				X	X	X	X	X	X
History endo/ecto parasites		X				X	X	X	X	X	X
Blood sample		VP [e] 5.2 ml				VP+FP [e, h] 5.7 ml	VP+FP [e, h] 5.7 ml	VP e 5.2 ml	VP [e] 5.2 ml	VP [e] 5.2 ml	VP [e] 5.2 ml

Unscheduled sick-patient clinic visits	`	Passive surveillance for 28 days (clinical malaria and other acute illnesses) (RDT/smear, Hb, dried blood spots for parasite genetics)					
Entomological events	0h	Day 2+4h	Day 7	Day 10	Day 14	Day 21	Day 28
Membrane feeding [e]	X	X	X	X	X	X	X
Direct feeding			X[g]				

Visit 1: Pre-Screening interview
Visit 2: Screening, Consent & Enrolment. First treatment dose given under direct observation.
Visits 3 and 4: Treatment visits. 2nd and 3rd treatment doses given under direct observation. In exceptional cases doses of day 1 and 2 can be taken at home.
Visits 5 to 10: Scheduled follow-up visits for assessment of efficacy parameters

a. Patients can be pre-study screened any time from visiting the OPD. The figure of -1 hour is provided for illustration purposes only.
b. The day of enrolment is always considered as Day-0. Doses given under direct observation. In exceptional cases doses of day 1 and 2 can be taken at home.
c. Visit window= number of days an actual subject visit may fall outside of the planned protocol schedule visit to still meet protocol requirements. It is preferential to stick to the scheduled days of visit. However, if this is not feasible (e.g. due to other commitments of the patient) then it preferable to allow flexibility in the schedule. The date of actual visit will always need to be recorded in the CRF.
d. Enrolment blood sample (0.6 mL) by venepuncture or fingerprick: Haemoglobin level (0.025 mL), malaria smear / RDT (0.025 mL), Pellet or Dried blood spots (DBS) for PCR (0.5) will be used for host genetic markers of drug metabolism (0.25 ml). Blood spots will also be stored for parasite genetic to differentiate reinfections from recrudescences in treatment failures (if any) (0.25 mL).
e. Baseline and follow-up blood sample (5.2 mL) by venepuncture: malaria smear / RDT (0.025 mL), haemoglobin level (0.025 mL), sero-diagnosis for Strongyloides stercoralis, filter paper dried blood spots for PCR (0.15 mL), membrane feeding (~1 mL), drug levels (~4mL to obtain ~2mL plasma) (see also Table 2, page 15. The malaria smear will be collected for research purposes only, and read days to weeks later. Malaria smears will not be used for point of care. If participants are symptomatic (e.g. fever) the pLDH band of the malaria RDT will be taken for point of care.
f. Membrane feeding will be used to assess: Mosquito survival (daily up to 21 to 28 days after feed; Oocyst and sporozoite prevalence from day 10 after feed
g. Direct skin feeding in a sub-sample only
h. Finger prick blood sample on Day 2+4h and Day 7: drug levels (~0.5ml to obtain ~0.25ml plasma).

VP=vene puncture. FP=finger prick, Plac=Placebo DP, DP=dihydroartemisinin-piperaquine, IVM=ivermectin, Hb=haemoglobin, MS=malaria smear, Pf=Plasmodium falciparum

Table 2: Pharmacokinetic sampling schedule for rich (standard) and population pharmacokinetic study per study army [a]

			Main trial			Rich PK sub-study					Population PK[c]					Total
Sampling point			N=47 (all patients)			N=12					N=47 (only 2 extra points/patient)					N=47
Day	Hrs from enrolment	Hrs after IVM dose	"PK + membr. Feed. + Smear/RDT + DBS (5.2 mL)			PK sample only (4 mL)				Total mL	PK sample only (4 mL)				Total mL	Total per arm
			Sampling #	mL	N**	Sampling #	Extra visit?	mL	N**	mL	Sampling #	Extra visit?	mL	N**	mL	# of samples
IVM 0	0	0	B	5.2	47					5.2					5.2	47
0.04	1	1				1st	Yes	4	12	4						12
0.13	3	3				2nd	Yes	4	12	4						12
0.25	6	6				3rd	Yes	4	12	4						12
0.5	12	12				4th	Yes	4	12	4						12
0.96	23	-1				5th	Yes	4	12	4						12
IVM 1	24	0														
1.08	26	2				6th	Yes	4	12	4						12
1.17	28	4				7th	Yes	4	12	4						12
1.5	36	12				8th	Yes	4	12	4						12
1.96	47	-1				9th	Yes	4	12	4						12
IVM 2	48	0														
2.08	50	2				10th	Yes	4	12	4	1st	Yes	4[c]	9	4.5[b,d]	21
2.17	52	4	1st	5.7[b]	47	11th				5.7					5.7b	47
2.25	54	6									2nd	Yes	4[c]	17	4.5[b,d]	17
2.5	60	12				12th	Yes	4	12	4	3rd	Yes	4[c]	9	4.5[b,d]	21
3	72	24				13th	Yes	4.5[b]	12	4.5	4th	Yes	4.	9	4.5[b,d]	21
4	96	48				14th	Yes	4.5[b]	12	4.5	5th	Yes	4.	9	4.5[b,d]	21
5	120	72				15th	Yes	4	12	4	6th	Yes	4[c]	9	4.5[b,d]	21
7	168	120	2nd	5.7[b]	47	16th				5.7					5.7b	47
8	192	144				17th	Yes	4	12	4						12
10	240	192	3rd	5.2	47	18th				5.2					5.2	47
14	336	288	4th	5.2	47	19th				5.2					5.2	47
21	504	456	5th	5.2	47	20th				5.2					5.2	47
28	672	624	6th	5.2	47	21st				5.2					5.2	47
Total volume / patient (mL)				37.4				61.5		78			8[d]		46.4[d]	

a. The exact timing of the sampling points may change slightly when new pharmacokinetic information becomes available, but the maximum number of pricks and total number of samples per individual participant will not change.

b. In addition to the venous draws specified, each participant will have a maximum of 4 finger pricks (0.5ml each): one on Day 2+4h and Day 7 [all participants], plus one at Day 3 and one at Day 4 [rich PK] or one at each of the two popPK visits [popPK]. The finger prick volumes are included in the total volume per time point.

c. Six extra population PK sampling points are shown, but only 2 of these extra samples per patient (plus 2 finger pricks) will be taken from these 6 time points. These will only be taken at time points that are not part of the membrane feeding. Thus the total number of sampling points available are 9, including the baseline plus 6 from the main trial (5.2 or 5.7 mL each) plus 2 extra Population PK samples (4.5 ml each).

d. Total blood volume for the population PK study population includes the 37.4 mL taken as part of the main trial samples (7x5.2 mL) plus 8 mL for the 2 extra population PK specific samples (4 mL each), and 2 mL for the 4 fingerpricks (0.5 mL each), per patient = 46.4 mL. Total blood volume for rich PK will be 98.4 mL, including 82.8 within the first 10 days.

4. INTRODUCTION

4.1. MALARIA TRANSMISSION IN WESTERN KENYA

Western Kenya has the highest malaria transmission, one of the highest population densities in Kenya, and remains the most important source of malaria transmission nationally. The Kenyan Medical Research Institute (KEMRI) and the US Centers for Disease Control and Prevention (CDC) have been working in Siaya County, western Kenya for over 3 decades. Following the widespread introduction of insecticide-treated nets (ITNs) in the KEMRI/CDC Health and Demographic Surveillance Site (HDSS) study areas, the community prevalence of *P. falciparum* parasitaemia among children <5 years of age declined from over 70% in 1997 to around 40% in 2008[1] and entomologic inoculation rates (EIRs) dropped from >150 in the early 1990s[2] to <20 infectious bites per person per year as of 2010 (Bayoh et al, unpublished observations). Coincident with this change was a dramatic decline in malaria-specific and all-cause under-five mortality.[1]

However since 2008, transmission intensity and population parasite prevalence have stagnated, and the 2012 prevalence of malaria parasitaemia is still approximately 40% in children aged <5 years, 55% in those aged 5-15 years, and 25% in >15 years old. Explanations for this lack of progress include incomplete ITN coverage (67%), stock-outs of artemether-lumefantrine (AL)[3,4] and a change in vector behaviour and species distribution from the predominantly endophilic and endophagic *An. gambiae sensu stricto (s.s)* and *An. funestus*, towards *An. arabiensis* as the primary vector. *An. arabiensis* has a lower human biting index and is more likely to feed and rest outdoors and is thus less affected by ITNs or indoor residual spraying (IRS). More recently, a decrease in sensitivity to the pyrethroids in many *An. gambiae s.l.* and *funestus* populations resulting in a resurgence of the *An. funestus* population.[5,6,7]

4.2. MASS DRUG ADMINISTRATION FOR MALARIA

Individuals with asymptomatic parasitaemia play an important role in sustaining malaria transmission. Mass screen and treat (MSAT) and mass drug administration (MDA) strategies specifically address the asymptomatic population; in the former, all persons are tested by RDT, symptomatic or not, and those found to be positive are treated, while in the latter, all persons are treated without first testing for malaria. Western Kenya has a high burden of asymptomatic cases. Approximately 50% of parasitaemic individuals in community-based cross-sectional surveys did not report being febrile anytime during the previous two weeks. In an effort to decrease the parasite reservoir, in June 2013 KEMRI/CDC commenced a cluster randomized controlled trial of MSAT which will run until the end of 2015. Currently a separate proposal is being developed to assess the efficacy and cost-effectiveness of MDA in this region, as modelling by the Medical Research Council Centre for Outbreak Analysis & Modelling has shown that MDA is likely more efficacious and cost-effective in areas starting with high transmission (Walker & Ghani, unpublished data).

4.3. IVERMECTIN AS ADJUNCT THERAPY FOR MALARIA CONTROL

Aside from MDA with ACT's alone, adjunct treatment with ivermectin might offer a promising new tool to reduce transmission.[8] Ivermectin is a broad spectrum antiparasitic endectocide active against a wide range of internal and external parasites. It was originally introduced as a veterinary drug, predominantly in domestic livestock, but since 1987 has been widely used in human medicine.[9] Ivermectin at a dose of 150 or 200 mcg/kg is the first-line treatment for *Onchocerca volvulus* (the cause of river blindness), *Wuchereria bancrofti* (the cause of lymphatic filariasis), and *Strongyloides stercoralis* (roundworm, an intestinal helminth). Ivermectin at these doses is used as MDA in annual campaigns for the control of river blindness and lymphatic filariasis in endemic areas. To date more than 1.8 billion treatments have been distributed.[10] The ivermectin MDA campaigns have been reported to have a secondary effect of reducing intestinal helminths in humans,[11] and on ectoparasites such as nuisance insects including head lice, mites, bedbugs and scabies.[9,12]

Anopheles species are also highly sensitive to these drugs.[13] Laboratory and field studies have shown that ivermectin is also potently active against *Anopheles spp*. at concentrations present in human blood after standard drug administrations and reduces the vector's lifespan, the re-blood feeding capacity, and the female fecundity, hatch rate of their eggs and the survival of progeny larvae.[13,14,15] Recent field studies have shown an added killing effect of ivermectin MDA against malaria vectors. In Senegal, ivermectin MDA given to humans to control onchocerciasis and lymphatic filariasis, was shown to disrupt malaria parasite transmission by *An. gambiae s.s*.[16,17,18] Aside from affecting the vector, ivermectin also inhibits parasite sporogony.[19] An ongoing study to assess the transmission blocking effect of ivermectin when given in combination with artemether-lumefantrine, showed a significant, but short-lived effect of ivermectin 200 mcg/kg on mosquito survival.[20]

Ivermectin is also used as a cattle parasiticide, and laboratory studies at KEMRI, our study site in western Kenya, have shown that cattle treated with ivermectin have blood that is toxic to zoophilic malaria vectors, including *An. arabiensis*, which like many other vectors feeds on hosts other than humans, particularly cattle.[21] Thus, well-coordinated, seasonal treatment of cattle could suppress *An. arabiensis* populations, thereby reducing malaria transmission.[21] Current research efforts at this KEMRI site involve comparative tests of various avermectin compounds for their effects, mediated through the cattle blood meal, on survival and fecundity of *An. gambiae* and *An. arabiensis.*

Thus, the high sensitivity of the vectors to very low doses of ivermectin makes this a potent novel tool for malaria transmission reduction strategies.[13] What makes ivermectin particularly attractive is (1) that it has a completely different mode of action, with the potential to kill vectors that are resistant to pyrethroids or other insecticide classes such as bendiocarb, currently used for IRS in some malaria endemic areas, (2) it is able to kill exophagic and exophilic vectors that escape the killings effects of ITNs and IRS by biting and resting outdoors, and (3) it is likely to have a significant effect on transmission by targeting four out of five of the factors influencing vectorial capacity.[8,22]

4.4. EFFICACY AGAINST MALARIA VECTORS

During an MDA campaign for onchocerciasis in Senegal, a single ivermectin dose of 150 mcg/kg affected survivorship of *An. gambia s.s*. for up to 6 days, resulting in a likely reduction of malaria transmission for at least 11 days as a result of a change in mosquito age-structure.[17] Three *in vivo* studies have attempted to assess the long-term effect of ivermectin on mosquito survival, by

conducting feeding at least 7 days after administration of ivermectin (see table 2).[15,20,21,23] A single low dose of 200 mcg/kg showed no effect on mosquito survival at 14-days post-treatment,[15] while a repeated dose of 200 mcg/kg given on days 0 and 2 has shown a modest effect on survival 7-days post-treatment,[20] and a dose of 250 mcg/kg in a human volunteer a potent effect for more than 2 weeks post-treatment.[21,23] A recent study that determined the population level efficacy of MDA with single dose of 150 mcg/kg across three different West African transmission settings and showed that *A.gambiae* survivorship was reduced by 33.9% for one week following MDA, parity rates were reduced for more than two weeks, while sporozoite rates were reduced by >77% for two weeks.[24] No studies in humans have: (1) compared the effect of doses above 400 mcg/kg of ivermectin on the ability of anopheline vectors to transmit malaria (henceforth referred to as infectivity), or (2) evaluated the effect of any dose of ivermectin higher than 400 mcg/kg on mosquito survivorship.

Table 3: The in-vivo effect of ivermectin on malaria transmission

Reference	Dose	Study population	Species	Results
Foley 2000[23]	250 mcg/kg, once orally	Direct feeding on 1 human volunteer	An. farauti	Cumulative 12-day mortality rate for mosquitoes fed 0, 7, 10 and 14 days post-treatment was 100%, 95%, 93%, and 40% vs 10% in controls.
Fritz 2009[21]	600 mcg/kg, once subcutaneously	Direct feeding on 9 cattle	An. gambiae	Cumulative 10-day mortality rate for mosquitoes fed up to 2 weeks post-treatment was >90%. No eggs were deposited by mosquitoes that fed within 10 days of treatment.
Chaccour 2010[15]	200 mcg/kg, once orally	Direct feeding on 25 malaria-free volunteers	An. gambiae	Cumulative 12-day mortality rate for mosquitoes fed 14 days post-treatment showed no difference with controls.
Ouédraogo 2014[20]	200 mcg/kg, once (day 0) or twice (day 0+2), orally	Membrane feeding on 120 adult males with asymptomatic malaria	An. gambiae,<br>An. funestus	Cumulative 10-day mortality rate for mosquitoes fed 7 days post single dose showed no difference versus placebo, while repeated dose showed a modest reduction in mosquito survival.
Alout, 2014[24]	150 mcg/kg, once	As part of MDA in 3 west African countries	An. gambiae	33.9% reduction in survivorship of mosquitoes for 1 week, and >77% reduction in sporozoite rates for 2 weeks.

4.5. MODELLING: POTENTIAL IMPACT OF IVERMECTIN

Modelling by Imperial College London has shown that adding 3 days of ivermectin 150 mcg/kg/day to MDA with dihydroartemisinin-piperaquine (DP) would potentially interrupt transmission faster and in higher prevalence areas than MDA alone. However, it also showed that higher doses and repeat doses

spread over 3 days given together with the ACT, have a greater effect than a single low dose ivermectin.[25]

Figure 1: Potential impact of 3-day low dose (150 mcg/kg) ivermectin when added to DHA-piperaquine as MDA (Slater et al, JID 2014)[25]

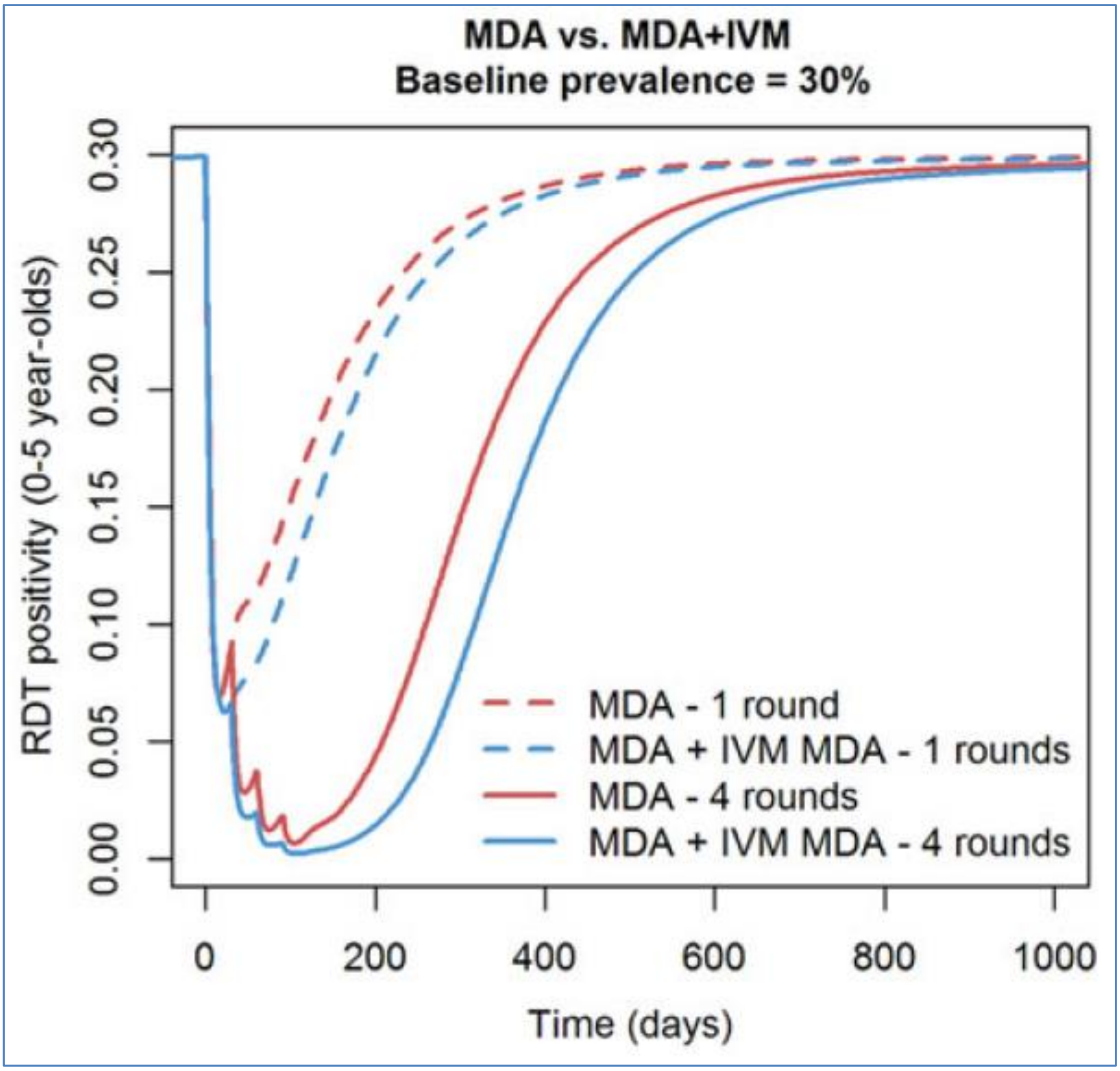

4.6. SAFETY OF IVERMECTIN IN HUMANS

Ivermectin's main mechanism of action in invertebrates is the opening of glutamate-gated chloride channels, resulting in flaccid paralysis and death.[26] Glutamate-gated chloride channels do not exist in humans. Other weakly sensitive channels are found in the human central nervous system, but the blood-brain barrier curbs drug access to these channels.[27] These features explain ivermectin's excellent safety profile.[8]

The standard dose of ivermectin in the control of onchocerciasis and lymphatic filariasis is 150 to 200 mcg/kg, and was based on earlier dose finding studies comparing 100, 150 and 200 mcg/kg which confirmed that a single-dose of 150 micrograms of ivermectin/kg was equally as effective as 200

mcg/kg for treatment of active onchocerciasis in patients with high microfilarial counts.[28] Since 1987, 1.8 billion doses of 150-200 mcg/kg have been safely administered around the globe.[10] The current standard dose for head lice is 400 mcg/kg,[29] which has been found to be safe and well tolerated.[30]

Ivermectin is safely used in pregnancy. In a study in Liberia, 200 women treated with ivermectin 150 µg/kg were inadvertently found to be pregnant. In comparison with untreated mothers in the same population, no significant differences in birth defect rates, development status or disease patterns could be found.[31] These findings were later confirmed in hundreds of women in Cameroon,[32] Mali,[33] Ghana[34] and Uganda.[35] As a result, since 1998, pregnant women in onchocerciasis-endemic areas are no longer excluded from ivermectin treatment.[36]

Four recent studies testing single-doses of 800-2,000 mcg/kg showed all the tested doses to be safe and well tolerated (see table 1).[37,38,39,40,41] In 100 adult males with onchocerciasis in Ghana, Awadzi et al compared doses of ivermectin up to 800 mcg/kg and found no difference in the tolerability or safety of the different doses.[37,38] In a safety, tolerability and pharmacokinetics study in 68 healthy, non-pregnant adults in the USA, Guzzo et al compared doses of ivermectin up to 2,000 mcg/kg and found no difference in tolerability or safety between doses.[39] In 657 adult males with onchocerciasis in Cameroon, Kamgno et al compared doses of ivermectin up to 800 mcg/kg and found an increased rate of transient mild visual side effects,[40] without structural abnormalities upon ophthalmological exam.[41]

The only known severe adverse events have been in individuals with *Loa loa* due to lysis of parasites, however *Loa loa* is not present in Kenya.[42] In other areas rapid assessment of *Loa loa* is recommended before ivermectin administration.[43]

Table 4: Studies of safety and tolerability of ivermectin incorporating dosages ≥800 mcg/kg.

Reference	Highest single dose	Total study population	Adverse events: increased vs control
Awadzi 1995, 1999[37,38]	800 mcg/kg	100 adult males with onchocerciasis in Ghana	No
Guzzo 2002[39]	2,000 mcg/kg	68 healthy adults, non-pregnant, in USA	No
Kamgno 2004[40,41]	800 mcg/kg	657 adult males with onchocerciasis in Cameroon	Transitory mild visual side effects, without structural abnormalities upon ophthalmological exam

4.7. INTERACTION: DP AND IVERMECTIN

DP and ivermectin have, to the best of our knowledge, never been administered simultaneously.

Piperaquine, the long-acting component of DP, is metabolized by and is an inhibitor of CYP3A4.[44] There is a potential for a several-fold increase of piperaquine plasma concentrations when it is co-administered with other CYP3A4 substrates (due to competition) and, especially, with CYP3A4 inhibitors.[44]

Dihydroartemisinin (DHA), the short-acting component of DP, is not metabolized by cytochrome-P450, but is deactivated via glucuronidation catalyzed by UDP-glucuronosyltransferases, in particular

UGT1A9 and UGT2B7.[45] DHA has been shown to induce CYP3A activity and also up-regulate CYP2C19 and CYP2B6.[45] DHA is a known inhibitor of CYP1A2.[44]

Ivermectin is primarily metabolized by cytochrome CYP3A4.[46] In vitro studies using human liver microsomes suggest that ivermectin does not significantly inhibit the metabolizing activities of CYP3A4, CYP2D6, CYP2C9, CYP1A2, and CYP2E1.[46]

When DP and ivermectin are administered together, there may be some competition for CYP3A4. In particular the CYP3A4-inhibitory properties of piperaquine may lead to an increased availability of ivermectin. As ivermectin is not a CYP3A4-inhibitor, the potential increase in the availability of piperaquine due to competition is expected to be low. Using plasma concentrations we will monitor this potential interaction of ivermectin and piperaquine, and expect that it could positively increase the duration of the mosquitocidal effect of ivermectin when used in combination with DP.

5. JUSTIFICATION FOR THE STUDY

5.1. WHY IS THIS STUDY NEEDED NOW?

The National Malaria Control Unit in Kenya and the Siaya County Health authorities in Siaya County are exploring options that can help towards aggressive transmission reduction to eventually reach pre-elimination of malaria in western Kenya. The stagnation in the decline of transmission seen in the KEMRI/CDC HDSS sites despite high ITN coverage for over a decade and ACT use, the potential for the vector population numbers to increase with increasing pyrethroid resistance, combined with the insight from recent mathematical modelling that high coverage of ITNs and ACTs alone are insufficient to interrupt transmission in areas starting with high levels of transmission such as western Kenya, make the evaluation of alternative options imperative.

Ivermectin offers a potent novel tool for malaria transmission reduction strategies, targeting both the vector and malaria parasites. It offers the possibility to reduce vectorial capacity, kill exophagic and exophillic mosquitoes, and stem resistance to insecticides and antimalarial drugs. Western Kenya is currently a site of an MSAT trial (KEMRI #2380), and soon MDA, with dihydroartemisinin-piperaquine (DP). The results of the limited number of field studies and of the modelling studies suggest that ivermectin can be a worthy addition to the arsenal of available tools to control and potentially interrupt malaria transmission. However higher doses may be needed for greater impact. This requires further dose finding and safety studies to help identify the best regimen(s) required to reach this goal.

5.2. OTHER RELEVANT RESEARCH ONGOING ELSEWHERE

We searched the WHO International Clinical Trials Registry Platform (ICTRP), which is the main central database containing the combined trial registration data sets from all major trial registries.

Only one study was identified evaluating the effect of ivermectin on malaria transmission. This clinical trial in Burkina Faso has been completed.[20] The study randomized 120 individuals with asymptomatic malaria to treatment with artemether-lumefantrine (AL) alone or in combination with one (day 0) or two doses (day 0+2) of ivermectin (200 mcg/kg) in a double-blind randomized trial. Outcomes were clinical safety and 10-day survival of mosquitoes that were membrane fed the blood of participants 1, 3 and 7 days after treatment. The AL-ivermectin drug combination was well tolerated. Mosquitoes

experienced a 3- to 4- fold reduced survival when feeding on day 1 and day 3 after ivermectin. The double dose ivermectin improved the duration of the mosquitocidal effect and showed a modest reduction in mosquito survival until day 7. These findings show a significant but short-lived effect of ivermectin (200 mcg/kg) on mosquito survival rates and support a role for ivermectin in preventing malaria transmission.

6. HYPOTHESIS

High dose ivermectin at 600 mcg/kg daily for 3 days is well tolerated and safe and will have a longer lasting mosquitocidal effect and a greater potential impact on malaria transmission.

7. AIM & OBJECTIVES

The overall aim of the study is to compare the effect of 3-day courses with ivermectin 0, 300, 600 mcg/kg/day when given in combination with standard 3-day course of dihydroartemisinin-piperaquine, on mosquito survival.

7.1. PRIMARY OBJECTIVE

To determine the safety and efficacy of ivermectin 0, 300, 600 mcg/kg/day for 3 days, when provided with a standard 3-day course of dihydroartemisinin-piperaquine (DP) for uncomplicated malaria, on 14-day mosquito survival after feeding experiment performed at 7 days after treatment (i.e. 5 days after the last dose of ivermectin).

7.2. SECONDARY OBJECTIVES

1. To determine the effect of different doses of ivermectin on oocyst and sporozoite rates.
2. To determine the effect of different doses of ivermectin on other ectoparasites (e.g. head lice, scabies).
3. To determine the pharmacokinetic profile of the different ivermectin regimens
4. To determine if ivermectin interacts with the pharmacokinetics of piperaquine
5. To determine whether the addition of ivermectin to DP interacts with the ability of DP to clear asexual and sexual stage parasites
6. To determine the role of genetic variants of CYP3A4 activity in metabolizing ivermectin
7. To determine the effect of direct feeding versus membrane feeding on mosquito survival

8. DESIGN & METHODOLOGY

8.1. OVERVIEW OF DESIGN

Double-blind placebo-controlled, parallel-group, 3-arm, superiority trial to determine the effect of different doses of ivermectin on mosquito survival. The unit of randomization will be the patient, and the unit of analysis will be the mosquito. The primary endpoint will be mosquito survival 14 days after a feed on blood from a patient who has started ivermectin 7 days earlier (i.e. about 5 days after the last dose of ivermectin with a 3-day regimen administering ivermectin at 0, 24, and 48 hours [days 0, 1 and 2]). Because mosquito feeding involves approximately 100 mosquitoes per feed, the study will

use a cluster randomized design with the patient as the cluster unit. The study will have a nested rich pharmacokinetic component in the first 12 patients per study arm, and a sparse sampling population pharmacokinetic component in the remaining patients.

8.2. DESIGN CONSIDERATIONS

8.2.1. Why patients with malaria?

The study will enrol patients with symptomatic uncomplicated malaria instead of asymptomatic patients with malaria parasites (carriers) or malaria negative individuals who are the predominant target population in mass drug administration campaigns. The rationale for this is that this is a labour intensive study requiring very frequent patient follow-up and blood sampling and thus a major commitment from study participants. Symptomatic patients are more likely to be agreeable to hospital admission and frequent out-patient visits than asymptomatic patients or other volunteers. The frequent follow-up is potentially also more beneficial to the patients with symptomatic malaria than asymptomatic patients.

8.2.2. Ivermectin dose considerations

We propose to study 300 and 600 mcg/kg/day dose of ivermectin which was based on the following considerations and pharmacokinetic modelling. The highest dose of ivermectin used in MDA campaigns for onchocerciasis is 800 mcg/kg given as a single dose (i.e. about 48 mg in an adult male weighing 60 kg, which is 16 tablets of 3 mg ivermectin). The pharmacokinetic profile of this single 800 mcg/kg dose was used to design a 3-day regimen that would achieve a similar C-max after the third dose. Since the highest dose of ivermectin used in humans that was tested and found to be well tolerated and safe is 2000 mcg/kg given as a single dose (40 tablets of 3 mg), this provides a large margin of safety. The middle group was chosen at 50% of the highest dose to allow for a dose response in terms of tolerance and efficacy.

Pharmacokinetic modelling using Monte-Carlo simulation of 1000 subjects assuming the PK parameters in Figure 2 (page24) with 30% variability were used. The PK parameters used were obtained from El-tahtawi et al. (2008)[47] to simulate different dosing regimens that would fall within the safety limits of Ivermectin. The simulated data are in excellent agreement with actual data observed in a dose finding study by Guzzo et al. 2002[39] (which indicated proportional pharmacokinetics at doses ranging from 30-120 mg), thus giving confidence in the parameters used in the simulations. The model showed that the Cmax in 1000 simulations associated with a single dose of 800 mcg/kg (i.e. 48 mg in 60 Kg adult) was estimated at 108 ng/ml and the 95% percentile as 164 ng/ml. A regimen of 600 mcg/kg/day for 3 days would give a similar Cmax (111 ng/mL) and corresponding 95% percentile (161 ng/mL) as the single dose 800 mcg/kg regimen (Figure 3 and Table 5). A regimen of 300 mcg/kg/day for 3 days would give approximately half those values. Both 3-day regimens were predicted to increasing the time that ivermectin concentrations remain above the lethal concentration 50 (LC50) of 16 ng/ml[19] from 46 hours with the 800 mcg/kg single dose to 86 and 162 hours with the two 3-day regimens, respectively. The 16 ng/mL dose was chosen as this is the median of three LC50 concentrations in-vivo reported to date.[16,19,20]

Figure 2: Cmax and 95% percentiles with single dose 48 mg dose (800 mcg/kg)

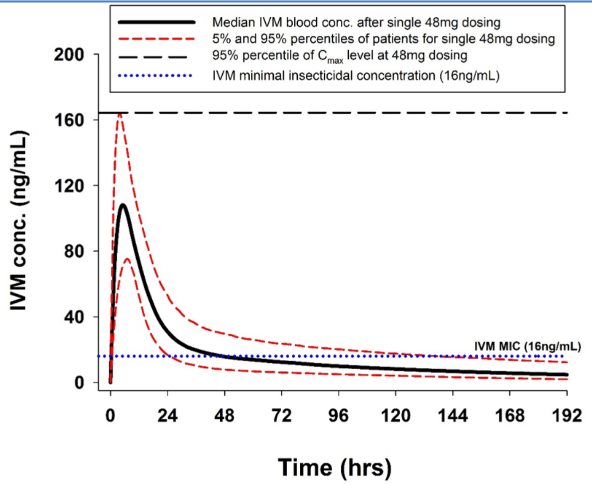

Monte-Carlo simulation of 1000 subjects assuming the PK parameters below with 30% variability.

Parameter	Value	Assumed Variability
CL/F (L/hr)	11.8	30%
Vc/F (L)	195.0	30%
Q (L/hr)	18.9	30%
Vp (L)	882	30%
K_a (hr^{-1})	0.24	30%

48mg one single IVM dose	Median	5% Percentile	95% Percentile
C_{max} (ng/mL)	108.1	75.3	164.4
Time above IVM MIC (hrs)	46	24	136

Figure 3 Compatible 300 and 600 mcg/kg/day 3-day regimen achieving similar Cmax and 50% of Cmax levels compared to the single 800 mcg/kg dose

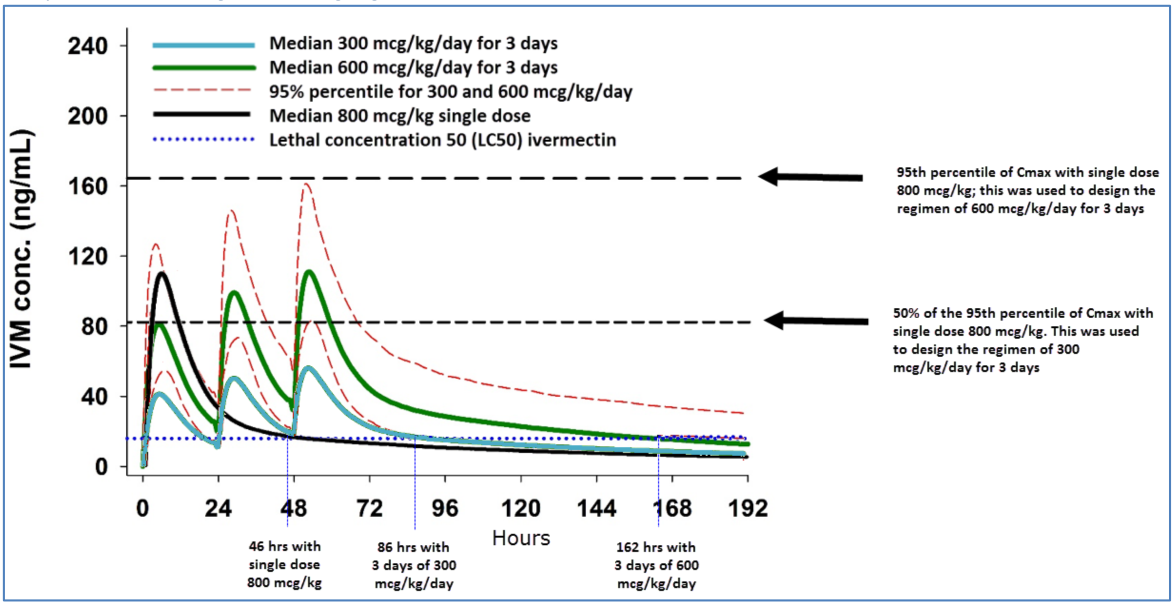

Table 5 Summary of simulated Cmax and median time above minimum inhibitory concentration (MIC)

Ivermectin Dosing Regimen	C_{max} (ng/mL)<br>Median (5th-95th percentiles)	Hours above MIC (16ng/mL)<br>Median (5th-95th percentiles)
48mg single dose<br>(= 800 mcg/kg, 60 kg)	108.1 (75.3-164.4)	46 (24-136)
36mg daily for 3 days<br>(= 600 mcg/kg/day, 60 kg)	111.0 (83.2-161.2)	162 (90-322)
18mg daily for 3 days<br>(= 300 mcg/kg/day, 60 kg)	55.4 (41.6-80.6)	86 (67-179)

8.2.3. Parallel versus dose-escalation design

The proposed study uses a standard parallel dose comparison design, comparing the 2 intervention arms with the placebo arm at the end of the study. Because the Cmax levels and the 95th percentile concentrations in the proposed highest dose group of 600 mcg/kg/day will be equivalent to the Cmax found with single dose 800 mcg/kg (see section 8.5.1.2), this parallel study design is considered appropriate because there is sufficient experience in humans with the 800 mcg/kg single dose regimen, which has been administered to hundreds of patients before as part of MDA campaigns for onchocerciasis. Because doses up to 2,000 mcg/kg (i.e. ten times the dose currently approved by the US FDA) are known to be well tolerated (see section 4.6, page 19), a dose escalation study, where we would observe the lower dose group first prior enrolling patient in the higher dose group is therefore not required.

8.2.4. Justification for host genetic studies

The cytochromes P450s (CYPs) are the major enzymes involved in drug metabolism. To be able to interpret variations in the pharmacokinetic drug profiles of piperaquine and ivermectin and any drug interactions we need to determine the genes encoding CYP enzymes (see section 4.7, "Interaction: DP and Ivermectin", page 20).

8.2.5. Direct skin feeding vs membrane feeding

The primary endpoint is based on membrane feeding of mosquitoes using blood obtained by venepuncture from patients recently treated with ivermectin. There are some indications that direct feeding (e.g. allowing [uninfected] mosquitoes to feed on the arm of the study participant by holding it in a cage with mosquitoes [see Figure 6, page 44]) results in higher mosquito mortality. It has been hypothesized that this is due to difference in drug level in venous versus capillary blood or due to drug accumulation in subcutaneous fat tissue or increase exposure of the mosquito to ivermectin through other means like perspiration. Ivermectin feeding studies with direct feeding on humans[23] (Foley 2000) and cattle[21] (Fritz 2009) have shown a longer mosquitocidal effect (>2 weeks) in comparison to studies using membrane feeding < 7days,[20] suggesting higher exposure. All pharmacological studies concerning ivermectin concentrations from humans used venous blood samples. The exception to this is one report by Baraka et al, from 1996[48] which demonstrated that ivermectin accumulates at 2-3-fold higher concentrations in fat, dermal, and fascia tissues compared to venous blood concentrations. Piperaquine, the long-acting component of DP, has also been found at 1.4-1.6-fold higher concentrations in capillary blood than venous blood.[49,50] Therefore, ivermectin may circulate at higher concentrations in capillary venuoles and arterioles than is predicted from venous blood concentrations. This is important as mosquitoes blood feed from subdermal venuoles and arterioles, not primary veins and arteries.

There have been no studies doing a head to head comparisons of direct feeding and membrane feeding on mosquito mortality following ivermectin. However, previous studies looking at infectivity (i.e. the ability of the vector to develop oocysts and sporozoites when ingesting gametocytes) showed significant differences in terms of infectivity in favour of direct feeding (odds ratio 2.39).[51] Although the mechanisms involved in infectivity studies may differ from studies addressing the killing effect of ivermectin this recent infectivity study indicates the importance of addressing the potential that the feeding method to expose mosquitoes to ivermectin may be an important effect modifier, and that studies using membrane feeding may potentially underestimate the true effect of ivermectin.

However direct skin feeding by about 50 mosquitoes is unpleasant to the human host and is labour intensive. We will therefore pilot the importance of direct feeding in the first 27 patients who provide additional consent by comparing mosquito mortality rates in clusters that are fed directly with mosquitoes clusters that are fed using standard membrane feeding. Only a single time point will be used for this (day 7 after start of ivermectin, which is the feeding time point used for the primary endpoint).

The results will be presented to the TSC and DMEC who will make a recommendation for the rest of the study. If the results show that the killing effect is superior with direct feeding then the study will continue to use direct feeding in all remaining patients (on day 7 only) in parallel to membrane feeding (all feeding days) in those that consent to direct feeding. Superior will be defined as a relative difference of 30% [relative risk 1.3]) regardless of whether this is statistically significant; e.g. if the mosquito mortality by day 14 after membrane feeding is 31.2%, and with direct feeding this is 40.6% or greater.

8.3. STUDY SETTING

8.3.1. *Study area*

The study will be conducted in Kisumu and Siaya counties, western Kenya, which have some of the highest rates of malaria transmission in Kenya. The community prevalence of *P. falciparum* parasitaemia among children <5 years of age declined from over 70% in 1997 following the widespread introduction of ITNs and has stagnated around 40% since 2008 despite sustained use of ITNs in the communities. To address the large proportion of asymptomatic infections in the population, KEMRI is conducting a series of large scale intervention studies in the area (population >100,000) involving mass screen and treat (MSAT) with DP (2013-2015) and mass drug administration (MDA) with DP.

8.3.2. *Clinical setting*

The study will be conducted in the Provincial Hospital in Kisumu, western Kenya recently renamed to the Jaramogi Oginga Odinga Teaching and Referral Hospital (JOOTRH). Dependent on the recruitment rate, enrolment will be expanded to other health facilities, based on a list of government or private (e.g. mission) health facilities in Kisumu and Siaya counties. Any additional sites will be selected purposefully, based for instance on case burden and proximity to JOOTRH. JOOTRH is a major tertiary care hospital with self-contained paediatric, medical, and obstetric wards, as well as an intensive care unit with mechanical ventilators and telemetry capabilities. Almost 25,000 outpatients are treated for clinical malaria at JOOTRH annually, of which one-third are laboratory confirmed. Approximately 20% of these patients are 18-50 years old. On a daily basis around 4.6 adults (18–50 years old) test positive for malaria in JOOTRH’s outpatient clinic.

8.3.2.1. Suggested expansion facilities

1. *Kisumu District Hospital (KDH)*, which is located just over 1 mile from the JOOTRH (about 5 minutes by car). KDH is a 195-bed government facility located in Kisumu city centre. Because of its close proximity, KDH can act either as a main recruitment site or a satellite recruitment site for JOOTRH with screening activities only and referral of eligible patients for enrolment to JOOTRH.

2. *Siaya District Hospital*: Siaya Town, Siaya County, western Kenya. The 260-bed hospital is located in an area of high perennial malaria transmission in the lake region and serves mainly a rural population. SDH is the main public hospital in the district, with a total of 260 beds.
3. *Vihiga District Hospital:* This is a 160 bed Government Hospital in Mbale town, just 30 to 45 min drive from JOOTRH in Kisumu.
4. *Lumumba Sub-County Hospital (LSCH)*, is located just over 1 mile from the JOOTRH (about 5 minutes by car). LSCH is a 15-bed government facility located in Kisumu city centre and caters to a large number of outpatients. Because of its close proximity, LSCH can act either as a main recruitment site or a satellite recruitment site for JOOTRH with screening activities only and referral of eligible patients for enrolment to JOOTRH.
5. *Migosi Sub-County Hospital (MSCH)*, is located just over 2 miles from the JOOTRH (about 10 minutes by car). MSCH is a 12-bed government facility located in Kisumu city centre centre and caters to a large number of outpatients. Because of its close proximity, MSCH can act either as a main recruitment site or a satellite recruitment site for JOOTRH with screening activities only and referral of eligible patients for enrolment to JOOTRH.
6. *Rabuor Sub-County Hospital (RSCH),* is located just over 7 miles from the JOOTRH (about 15 minutes by car). RSCH is a 30-bed government facility located just outside of Kisumu and caters to a large number of outpatients. Because of its close proximity, RSCH can act either as a main recruitment site or a satellite recruitment site for JOOTRH with screening activities only and referral of eligible patients for enrolment to JOOTRH.
7. *Rota Dispensary,* is located just over 8 miles from the JOOTRH (about 25 minutes by car). Rota is a government facility located just outside Kisumu and caters to a large number of outpatients. Because of its close proximity, RSCH can act as a satellite recruitment site for JOOTRH with screening activities only and referral of eligible patients for enrolment to JOOTRH.
8. *Avenue Hospital,* is located just across the road from the JOOTRH (about 1 minute by car). Avenue is a private facility that caters to a large number of outpatients. Because of its close proximity, Avenue can act as a satellite recruitment site for JOOTRH with screening activities only and referral of eligible patients for enrolment to JOOTRH.
9. *Aga Khan Hospital,* is located just over 1 mile from the JOOTRH (about 5 minutes by car). Aga Khan is a private facility that caters to a large number of outpatients. Because of its close proximity, Aga Khan can act as a satellite recruitment site for JOOTRH with screening activities only and referral of eligible patients for enrolment to JOOTRH.

8.3.2.2. Backup list for expansion facilities

A list of examples of backup expansion facilities is included below in case the first choice of expansion facilities turn out to be less eligible because of changes in patient numbers or logistical constraints (e.g. no space, other competing studies ongoing or planned etc).

1. *Mukumu Mission Hospital:* This is a 228-bed mission hospital located on the road between Kisumu and Kakamega town in an area with moderate malaria transmission
2. *St Mary's Hospital, Mumias*: This is a 200 bed mission hospital located in Mumias town, 1.5 hours drive from Kisumu, in an area with moderate malaria transmission.
3. *Kakamega Provincial General Hospital:* This is a large 450 bed hospital in Kakamega town in Kakamega County.

4. *Busia District Hospital:* 185-bed government facility, located in Busia town at the Kenyan-Ugandan border, Busia County, Western Province. Distance to Kisumu 110km, appr. 2 hours drive. Intense malaria transmission.
5. *Bondo District Hospital*: This 50-bed hospital is located in Bondo town, Siaya County, western Kenya, located 50 kilometres west of Kisumu in an area with intense malaria transmission.
6. *Homa Bay District Hospital*: This is a 280 bed government facility in Homa Bay County, appr. 2 hours drive (110km) from Kisumu, in an area of intense malaria transmission
7. *Nyamira District Hospital:* 203- bed government facility, located in Nyamira County, 1.5 hours drive from Kisumu.

8.4. ELIGIBILITY CRITERIA

8.4.1. Inclusion criteria

- Symptomatic, uncomplicated *Plasmodium falciparum* infection
- Positive malaria microscopy or malaria RDT (pLDH)
- Age: 18-50 years
- Provide written informed consent
- Agree to be able to travel to clinic on days: 1, 2, 7, 10, 14, 21, and 28

8.4.2. Exclusion criteria

- Signs or symptoms of severe malaria
- Unable to provide written informed consent
- For women: pregnancy or lactation
- Hypersensitivity to ivermectin or DP
- Qtc > 460 ms on ECG
- Body Mass Index (BMI) below 16 or above 32 kg/m2
- Haemoglobin concentration below 9 g/dL
- Taken ivermectin in the last month
- Taken dihydroartemisinin-piperaquine in the last 12 weeks
- *Loa loa* as assessed by travel history to Angola, Cameroon, Chad, Central African Republic, Congo, DR Congo, Equatorial Guinea, Ethiopia, Gabon, Nigeria and Sudan
- History and/or symptoms indicating chronic illness
- Current use of tuberculosis or anti-retroviral medication
- Previously enrolled in the same study

8.5. INTERVENTIONS

8.5.1. Trial Medications and interventions

Participants will be randomized (see section 8.10, page 37) to one of 3 arms:

1. Dihydroartemisinin-piperaquine (DP) alone, DP plus ivermectin 0 mcg/kg/day for 3 days (“placebo” arm).
2. DP plus ivermectin 300 mcg/kg/day plus 300 mcg/kg/day ivermectin-placebo for 3 days (“300 mcg/kg” arm).

3. DP plus ivermectin 600 mcg/kg/day (“600 mcg/kg” arm).

Patients will receive their weight-based doses of DP and ivermectin/placebo as directly observed therapy in the out-patient clinics or in-hospital if they were admitted for the pharmacokinetic sub-studies.

8.5.1.1. Dihydroartemisinin-piperaquine (DP)

All study participants will be treated for malaria with the appropriate weight-based dose of DP according to the Ministry of Health National Guidelines for Malaria Treatment, which recommend DP as the second-line drug for malaria treatment in Kenya. DP was selected as the drug of choice for the ongoing mass screen and treat study due to its comparable efficacy to artemether-lumefantrine (AL), the first-line drug, and because of the longer prophylactic effect against malaria (4–6 weeks, compared to 2-3 weeks for AL).

8.5.1.2. Ivermectin (IVM)

To ensure adequate dosing, ivermectin 3 mg or 6mg tablets will be administered per bodyweight (see 16.6 “Appendix VI. Product characteristics”, page 77).

8.5.2. Procedures for Drug handling & Accountability

8.5.2.1. Preparation and packaging

Each subject’s weight specific study drugs will be provided in drug containers or blister packs. This will be done by a pharmacy assistant who will be unblinded to the study. The full course will be prepared in one session, i.e. 3 containers/packs (one per day) (or 6 containers/packs; 2 per day one for DP and one for ivermectin/placebo) for a full course of DP or and ivermectin/placebo and will be kept in a subject specific study drug box after preparation until dispensing. Packaging used for all treatment arms will be identical, labelled and blinded to the contents.

8.5.2.2. Labelling of trial drug

Labelling will be in English and in accordance with local regulations, which will include the name of the study, the name, weight and study identification number of the participant, and his/her drug dose. The participant’s dose calculated per his/her weight will be displayed; for DP this will be the dose of DP, while for ivermectin/placebo only the total dose of ivermectin plus placebo will be displayed (the amount of ivermectin and placebo individually will not be displayed). Labelling may also include usage directions and staff contact number, precautionary measures to be observed when taking the drug.

8.5.2.3. Product Storage

All study drugs will be stored in a secure area with access limited to Investigator and authorised study site personnel, and under appropriate storage conditions. A description of the appropriate investigational product-specific storage conditions are specified on the investigational product pack label as described in more detail in 16.6 “Appendix VI. Product characteristics”, page 77.

8.5.2.4. Product accountability

The site-PI will be responsible for establishing a system for the correct handling of study drug to ensure that:

1. Deliveries of study drug from the sponsor are correctly received by a responsible person (e.g. pharmacist assistant)
2. Accurate records are maintained for the receipt of study drug, for the dispensing of study drug to subjects and for returned drug.
3. Certificates of delivery and return must be signed preferably by the investigator or authorised personnel and copies retained in the investigator file.
4. Study drug is to be handled and stored safely and properly and in agreement with the given storage instructions.
5. The study drug is to be prescribed only by the principal investigator, co-investigators or study site personnel authorised to do so by the principal investigator.
6. Study drug is dispensed only to study subjects in accordance with the protocol.
7. Subjects must return all unused medication and empty containers to the investigator.
8. At the end of the study, delivery records must be reconciled with records of usage and returned stock. Any discrepancies must be accounted for in writing.
9. Once accounted for, any returned and unused study treatment at the site will be returned to the sponsor for destruction or destroyed locally upon agreement with the sponsor. Drug destruction certificates will be issued that refers to the subject study numbers for subject specific medication that was destroyed.

8.5.2.5. Pharmacist assistant/dispenser

All efforts will be made for the preparation, packaging and labelling of the blinded study drug to be performed and documented in accordance with Good Manufacturing Practice (GMP). The sponsor will provide the pharmacist assistant with written instructions and GMP training on the preparation, packaging and labelling procedures.

8.5.3. Removal of Patients from Therapy or Assessment

Patients can discontinue from the study for any one of the following reasons.

1. Screening error resulting in incorrect enrolment (found that subject did not meet required inclusion / exclusion criteria)
2. Withdrawal of consent at any stage or subject not willing to continue in the study / voluntary discontinuation by the subject
3. Suspected or confirmed allergic reaction to the study drug (removal from therapy only)
4. Safety reasons as judged by the investigator, study safety monitor or DMEC (removal from therapy only)
5. Other

The patients who discontinue from the study treatment or from the study entirely will always be asked about the reason(s) for their discontinuation and the presence of adverse events. If a subject discontinues it should be established whether the subject:

1. Discontinues the study treatment, but continues their consent for the data capture up to that point, and to continue follow-up. These subjects will be considered ‘off study drug/on study’ and where feasible will follow the same schedule of events as those who continue the study intervention, except any adherence assessment. All of these patients will be followed until study end at 28 days from enrolment.

2. Discontinues all future activities in the study, but continues their consent for the data captured up to that point to be used in the research
3. Discontinues all future activities in the study and withdraws consent for any data captured to be used for the research

Every effort will be made to follow-up patients who discontinue due to drug related adverse events in order to determine the final outcome. If a subject discontinues due to drug-related adverse events, all the assessments that would have been carried out at the next scheduled visit will be conducted at day 28 where reasonably possible (unless consent is withdrawn). This will be recorded in the Case Record Forms (CRFs). The study drug will be returned by the subject. Subjects that have discontinued the study prematurely will not be replaced.

8.5.4. Discontinuation from storage of blood for future studies

If a subject discontinues it will also be established whether the subject:

1. Continues their consent for long term storage of the blood sample
2. Withdraws consent for long-term storage (for future studies and for genetic testing that is part of the main study protocol, but that may not yet have been conducted for that individual before de-identification of the dataset has occurred.

When a subject's consent for long-term storage is withdrawn, the stored sample will be destroyed and the withdrawal noted in the CRF. If the request is received after the dataset has been anonymised, the stored sample can no longer be withdrawn.

8.5.5. Adherence to study intervention protocol and strategies for retention

8.5.5.1. Adherence to study protocol and medication

Where feasible, study participants will be reminded about any follow-up visits through mobile phone contact. All information will be recorded on the appropriate sections of the CRF. Subjects judged to be non-compliant may continue in the study but will be counselled on the importance of taking their study medication as prescribed.

8.5.5.2. Strategies for retention

During pre-screening and consent procedures, potential participants will be asked whether they will be willing and able to comply with the frequent follow-up schedule and whether they need to travel out of the study area for an extended period during the follow-up period. The 'study catchment area' will be defined for each study site before the start of the study. All participants will be reimbursed for transportation costs to and from the clinic.

Detailed directions to the participant's homes as well as contact information, including mobile phone information, will be recorded prior to discharge. If participants do not return for scheduled follow-up visits, the study team will call them and ask them to come to the clinic for evaluation, offering transport reimbursement, or may visit their house to help arrange transport to the clinic if they are willing to come to the clinic, or, alternately, a study staff may go to their home for clinical evaluation and to assess if they still wish to participate in the study.

The participant's travel costs will be reimbursed as described in more details in section 11.9, Expenses reimbursement and incentives, page 55.

8.5.6. Prior and concomitant therapy

All concomitant medications taken during the study will be recorded in the appropriate sections of the CRF with indication, dose information, and dates of administration.

8.5.6.1. Permitted Medications during follow up period

During the treatment and follow-up phase of the study if a subject is diagnosed with malaria the investigator will prescribe antimalarial treatment based on the severity of the malaria illness (artemether-lumefantrine for uncomplicated malaria and parenteral artesunate or quinine for severe malaria).

8.5.6.2. Concomitant and prohibited Medications

Participants will be counselled to avoid concomitant and prohibited medications, specifically antimalarial drugs not prescribed within the trial protocol, or drugs that may be associated with QTc prolongation.

Prohibited medication

- Antimalarials not prescribed by the study: Chloroquine, halofantrine, mefloquine
- Diuretics (hydrochlorothiazide, furosemide)
- Drugs known to prolong the QT interval
    - Antimicrobials:
        - macrolides (e.g. erythromycin, clarithromycin, azithromycin, roxithromycin),
        - fluoroquinolones (e.g. ciprofloxacin, norfloxacin, levofloxacin, moxifloxacin, sparfloxacin)
        - pentamidine
    - Antifungals: ketoconazole, fluconazole, Itraconazole, posaconazole, voriconazole, caspofungin
    - Antiretrovirals: ARVs, specifically: indinavir, nelfinavir, atazanavir, saquinavir
    - Antiarrhythmic agents (e.g. amiodarone, sotalol)
    - Non-sedating antihistamines (astemizole, terfenadine)
    - Antipsychotics (neuroleptics): Haloperidol, Thioridazine
    - Antidepressants: Imipramin, Citalopram, Escitalopram
    - Antiemetics: Domperidone, Chlorpromazine, Ondansetron

Randomised participants who take prohibited medications resulting in the premature cessation of the study intervention, will remain in the trial and will be included in the primary, intention-to-treat analysis, but excluded from the per-protocol analysis.

8.6. ENDPOINTS / OUTCOME MEASURES

8.6.1. Primary efficacy outcome (see table Table 1):

Mosquito survival: Survival of mosquitoes at 14 days after feeding on blood taking from study participants who started the 3-day ivermectin and DP regimen 7 days earlier.

8.6.2. Secondary outcomes (see table 3):

- Mosquito survival: Survival of mosquitoes at each day up to day 21 or 28 after each feeding experiments performed at 0, 2 day+4h, 10, 14, 21, 28 days after start of treatment
- Oocyst and sporozoite prevalence: Occurrence of oocysts and/or sporozoites from day 10 onwards after each feeding as determined by PCR or Elisa (using pool testing)
- Malaria clinical and parasitological treatment response by day 28
- Plasma concentration profiles of piperaquine and ivermectin as described by standard pharmacokinetic metrics (e.g. $AUC_{0-\infty}$, $AUC0_{tlast}$, C_{max}, $t_{½}$, $t_{max,}$ etc).
- Ectoparasitic infection: bedbugs, head lice, scabies, etc as assessed by pre- and post-treatment questionnaire and physical examination.

8.6.3. Tolerability and Safety endpoints

8.6.3.1. Tolerability

- Any adverse events assessed in a general toxicity questionnaires

8.6.3.2. Safety

- Primary: Mydriasis quantitated by pupillometry [39]
- Secondary:
    - CNS effects
    - General toxicity
    - Serious adverse events
    - Haemoglobin concentrations
    - QTc interval (see section 9.4.3.2, page 45)
- Further criteria and monitoring for adverse events may be determined by the Data Monitoring and Ethics Committee (DMEC).

8.7. PARTICIPANTS TIMELINE

8.7.1. Overview Study Phases

The study plan and schedule of assessment is provided in Table 1, page 13. The participant's timeline consist of pre-screening visit (visit 1), Consent, screening & enrolment visit (visit 2), 2 subsequent treatment visits (3 and 4) on days 1 and 2, and 6 follow-up visits for assessment of efficacy parameters (visits 5 to 10). For those enrolled in the pharmacokinetic study additional visits for drug level sample are required as outlined in Table 2, page 15.

8.7.2. Visit 1: Pre-Screening interview

The purpose of the pre-screening visit is to identify potential participants with uncomplicated malaria who fulfil some of the other eligibility criteria that can be determined using assessments that are part of routine practice and do not require research specific activities. Uncomplicated malaria for pre-screening purposes will be considered any patient presenting to the clinic (usually the out-patient department) with a positive microscopy or RDT who is considered for oral malarial treatment by the clinic staff. These patients will be approached by a study team member or clinic staff trained by the study team. The patients will be given a pre-screening ID number and the study will be explained to them briefly, including the necessity to return to the clinic for multiple follow-up appointments or to

be admitted for a few days (for the standard pharmacokinetic study). If the patient responds that they are potentially interested in the study, a brief history for other eligibility criteria that can be assessed with routine practice will then be completed, including reconciliation of any medication the patient has been taken and potential presence of pregnancy and use of lactation. The results of any Hb tests conducted in the clinic will be copied to exclude moderate or severe anaemia (Hb<9/dL). If Hb tests results are not available a Haemocue or equivalent test can be performed. Alternatively the study team can determine the hb after consent is obtained as part of the baseline blood sample (see section 8.7.3.5, page 35).

8.7.2.1. Pre-Screening log

The investigator will keep a subject pre-screening log for all subjects considered for enrolment regardless of whether they were enrolled, which will be used to establish that the study sample was selected without bias. This pre-screening log will not contain names or other identifying information.

8.7.3. Visit 2: Screening, Consent & Enrolment

8.7.3.1. Consent

Patients who meet all eligibility criteria that were assessed during the pre-screening will be invited for a consent interview. Patients who are not eligible will be referred for care as appropriate. The details of the consent procedures are described in section 11.4, page 52.

8.7.3.2. Screening post-consent

After consent is obtained, further assessment will be conducted to determine if the subject fulfils the remaining eligibility criteria (screening). Because this requires study specific assessments, these screening procedures are only conduced after consent is obtained.

This includes an ECG to exclude QTc prolongation, confirmation that the patient has uncomplicated malaria (smear positive or RDT positive and ability to take oral medication), assessment of height and weight to determine BMI, and assessment of a travel history to countries with loa-loa. All women of childbearing age who are unsure of their pregnancy status, will be required to perform a pregnancy test at the time of enrolment prior to being enrolled into the study. Any woman who states that she is currently pregnant or breastfeeding, or who tests positive for pregnancy, will be excluded from the study and referred to a maternal health clinic.

All screening information will be recorded on the screening log form, which will contain the pre-screening ID number, and any study IDs once assigned to the patient (see section 8.7.3.3).

8.7.3.3. Assignment of study IDs

Subjects who provide informed consent and who meet all the eligibility criteria (for the criteria, see section 8.4, page 28) will be issued a study subject number. Once issued the study subject number they will be considered as ‘enrolled.’ This number is the subject's unique identifier and used to identify the subject on the CRFs. Subject numbers will be assigned strictly sequentially as subjects enter the study. Once a number has been assigned no attempt will be made to use that number again, for example if a subject discontinues or is a screening failure.

8.7.3.4. Clinical assessment

The subject's demographic data, and all relevant clinical information, including the previous and current medical history will be recorded in the CRF. A further clinical examination will be performed (including pupillometry, see section 9.4.3.1, page 45) and a further medical history taken, including the presence of any symptoms for endo- and ectoparasitic infection, that will serve as the baseline examination and captured on the CRF. A baseline blood sample will be drawn (see Table 2, page 15).

8.7.3.5. Baseline Laboratory Measurements

We shall record all laboratory results available as part of routine clinical care such as haemoglobin values, HIV status, and malaria test results. In addition to the standard of care tests, a venous blood sample will be taken by venepuncture or through a cannula (for those enrolled in the nested Pk study) (see Table 1, page 13 and Table 2, page 15).

8.7.3.6. Treatment allocation

Following the completion of all baseline procedures, the subject who fulfil al the eligbility crtieria and who have given informed consent will be allocated to 1 of the 3 treatment arms (as outlined in section 8.10, page 37). Subsequently, patients will receive their weight-based doses of DP and ivermectin/placebo as directly observed therapy (see section 8.5, page 28).

8.7.4. Visits 3 and 4: Treatment visits

They will return to the out-patient clinic on day 1 and 2 for the 2nd and 3rd dose of study drugs. In exceptional cases a patient will be permitted to take the study medication at home or the patient will be visited at home by study staff to administer the medication. A follow-up ECG will be taken just prior to and 4-6 hours after the last dose of DP+ivermectin on day 2.

8.7.5. Visits 5 to 10: Scheduled follow-up visits

They will return to the out-patient clinic for follow-up as specified (see Table 1, page 13 and Table 2, page 15). A questionnaire will assess the presence of signs and symptoms, including any adverse effects. A brief clinical examination will be performed and a venous blood sample will be taken for malaria diagnosis, haemoglobin levels, and drug levels. On visits 5 (Day 2+4h) and 6 (Day 7), drug levels will also be determined by finger prick. A final follow-up ECG will be taken on the day 28 visit. Participants will be asked to provide telephone numbers so that study staff may make every effort to follow-up patients who have missed scheduled visits as outlined in section 8.5.5, page 31.

8.7.6. Unscheduled visits

At any time, participants displaying signs or symptoms of severe malaria will be admitted to the inpatient ward for further evaluation and treatment free of charge. Blood samples for malaria smears, parasite genetics (filter paper dried blood spots) and haemoglobin will be taken if clinically indicated (e.g. documented fever ≥ 37.5 °C axillary, or a history of fever in the last 24 hours).

8.8. SAMPLE SIZE

8.8.1. Main trial

The study is powered for the primary outcome and is based on the following parameters: it requires a total of 141 participants (47 participants in the 0, 300 and 600 mcg/kg/day groups each) and is

powered at 80% to detect a relative increase of 30% (RR 1.300) in the 14-day post-feeding mortality rate from 24% in the control group (0 mcg/kg ivermectin) to 31.2% in the 300 mcg/kg/day group, and a 25% (RR1.246) increase from 31.2% with 300 mcg/kg/day to 38.9% in 600 mcg/kg/day recipients, measured from blood taken 7 days after the start of intake of ivermectin and using clusters of 100 anopheline mosquitoes allowing for 10% non-feeders (significance level: 0.05). The same sample size would give 90% power to detect a 35% [RR 1.348] increase from 24% (0 mcg/kg/day) to 32.4% (300 mcg/kg/day), and 27.7% increase [RR 1.285] from 32.4% (300 mcg/kg/day) to 41.3% (600 mcg/kg/day), The calculations assume an intracluster correlation coefficient (ICC) of 0.0622 and allow for 6.5% loss-to follow-up of participants by day 7 (i.e. 44 patients per arm contribute to the primary analysis).[20] The 10% non-feeding rate is based on current data from the same laboratories at KEMRI. The 24% mortality rate estimate by day 14 post-feeding in the control arm is average of observation at KEMRI (18.3%) and in a recent study in Burkina Faso, which showed a 21.2% mortality by day 10,[20] which when extrapolated with 4 additional days predicted a mortality of 29.7% by day 14. The ICC value of 0.0622 is based on the recent study in Burkina Faso that used a coefficient of variation of 0.5 in their sample size calculation and an estimated 20% mortality among mosquitoes fed on volunteers treated with 0 or 200 mcg/kg ivermectin (Bousema, personal communications).[20]

8.8.2. Nested rich pharmacokinetic study

A total of 36 participants (12 per arm) will be included to determine the standard pharmacokinetic profile of DP and of ivermectin when co-administered and when DP is given alone (with ivermectin placebo) as described in detail in section 8.11 “Pharmacokinetic (PK) studies”, page 37.

8.8.3. Nested direct feeding pilot study

A total of 27 participants (9 per arm) are required for the pilot study to determine whether direct feeding results in greater exposure to ivermectin and therefore greater killing effect than membrane feeding. This assumes that the study remains blinded and that therefore one third of the 24 patients fall in the placebo arm, and that about 8 are from the 300 and 600 mcg/kg/day arms each. The average killing effect in the 300 and 600 arms is taken as the mean of 25.03 and 32.54 = 28.79%. To show a relative difference of 66.7% (i.e. RR = 1.67) a sample of 8 per arm is required to detect a difference between 28.8% with membrane feeding and 48.1% with direct feeding (1.67 x 28.8%) with 80% power, alpha of 0.05 and ICC of 0.0622 as above. To accommodate for loss to follow-up, refusals or unsuccessful assays, 9 will be recruited per arm.

8.8.4. ECG monitoring

We anticipate that 42 of the 47 subjects can be followed until day 28 inclusive and contribute to the full ECG analysis. A sample size of 42 has 90% power to detect non-inferiority margin of equivalence is 18ms using a one-sided t-test (alpha 0.025) and assuming a true difference between the groups of 0 ms, and an average standard deviation of 35ms.[52,53]

8.9. RECRUITMENT STRATEGIES FOR ACHIEVING TARGET SAMPLE SIZE

The enrolment of the target sample size is scheduled to be completed in a 5 month period, requiring an average of 30 participants per month. The study site is chosen based on its potential to recruit at least 50 participants per month. Recruitment will start in 1 hospital and can be expanded to

approximately 3 hospitals if low recruitment rates are encountered. We have therefore included additional backup hospitals (see section 8.3.2 “Clinical setting”, page 26).

8.10. ASSIGNMENT OF INTERVENTIONS

8.10.1. Allocation

The study will use stratified randomization by BMI (2 strata) as this is an important determinant of the pharmacokinetics of ivermectin which also partly explains the difference by gender in the efficacy of ivermectin.[54] If more than one site will be involved, randomisation will also be stratified by site. Participants will be randomly assigned to 1 of the 3 study arms. The study statistician will computer-generate a randomization sequence using permuted block randomization with fixed/varying block sizes.

8.10.2. Blinding

The study will be double-blinded to all participants and study staff. Allocation concealment will be achieved by use of sealed opaque envelopes. All study participants in all 3 arms will receive standard dose DP. Additionally for the ivermectin component, the placebo group will received placebo-ivermectin tablets only, the 600 mcg/kg/day will receive active ivermectin only, and the 300 mcg/kg dose group will receive a combination of active ivermectin and ivermectin placebo tablets, such that each arm receives the same number of tablets in each weight strata.

8.11. PHARMACOKINETIC (PK) STUDIES

8.11.1. Overview

In each study arm, 12 patients will be enrolled in a rich pharmacokinetic study using frequent (rich) sampling per individual (26 samples per patient, Table 2, page 15) to determine the detailed pharmacokinetic profile of the 2 regimens and assess whether any drug interaction occurs with piperaquine that is of clinical relevance. The remaining 35 patients per arm contributed to a population PK study consisting of sparse PK sampling (maximum 13 samples per patient including baseline [1 venous sample], 6 scheduled visits as part of the main trial [6 venous and 2 finger prick samples] and 2 extra visits for population PK sampling [2 venous and 2 finger prick samples]).

The rich and population PK studies combined allow us to determine the main sources and correlates of variability in drug concentrations, including demographical, pathophysiological, such as body mass index, gender, smoking and other factors that might alter dose-concentration relationships.

Because this is a placebo controlled trial, the sampling methodology for the 47 patients in the ivermectin-placebo arm will be identical to that used for the 300 and 600 mcg/kg arms. The patients in the placebo-ivermectin arm will allow us to determine the piperaquine kinetic profile in the absence of ivermectin.

Finger prick blood draws will be performed at a maximum of 4 time points in addition to the venous blood draws. The aim is to compare the capillary and venous drug concentration levels as it has been hypothesized that these might differ for ivermectin, similar to other drugs including piperaquine. A difference between capillary and venous drug concentrations could help further explain any observed

difference in mosquito mortality between membrane and direct skin feeding (see also section 8.2.5 “Direct skin feeding vs membrane feeding”, page 25).

8.11.2. Standard pharmacokinetic study (rich sampling)

All of the 12 patients per arm will have venous blood sampled (4 ml whole blood to obtain 2 ml plasma, or 5.2 of whole if coinciding with a scheduled follow-up visit for the main trial) at baseline and each of 21 follow-up time points listed in Table 2, page 15. Additionally 4 finger pricks (0.5ml whole blood) will be taken at Days 2+4h, 3, 4 and 7. The total blood volume to be drawn from these patients is 98.4 mL whole blood over 28 days, 82.8 mL of which is taken during the first 10 days. The schedule in the first 8 days is illustrated in Figure 4. If more than 2 patients withdraw from the study without giving more than 12 samples, the withdrawing patients will be replaced. Outpatients who consent to the standard pharmacokinetic study will admitted in the hospital for the first 3 days. On admission, a cannula will be placed by venepuncture to limit the total number of venepunctures required for the first set of blood draws during the hospitalisation period. All venous blood draws performed will be drawn from the cannula provided that it is patent. Should a functioning cannula not be in situ and the participant require >1 additional in hospital venous blood draw, then a new cannula will be placed. If only 1 in hospital blood draw remains, than it will be taken by venepuncture. Cannulas will remain in the arms of patients admitted to the inpatient study for the duration of their hospitalization and will be removed at discharge. Samples will be taken by venepuncture (and finger prick) for patients during the subsequent out-patient follow-up.

*Figure 4: Approximate sampling time frame for rich pharmacokinetic study during first 8 days**

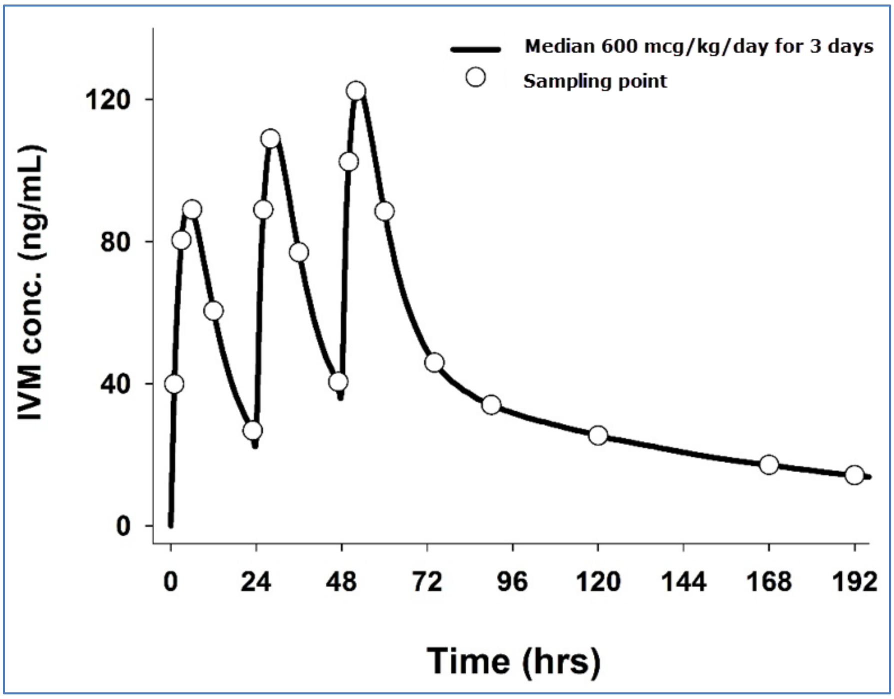

Additional sampling time points not shown include days 10, 14, 21, 28.

*The 600 mcg/kg/day dose is shown for illustrative purposes. Because the study is double blind, similar sampling schemes apply to all 3 arms.

8.11.3. Population pharmacokinetics (sparse sampling)

Each of remaining 35 patients per arm, not enrolled in the rich pharmacokinetic sub-study, contribute to the population pharmacokinetic study, which consists of 13 sampling points (Table 2), 7 of which coincide with the timing of sample for the membrane feeding (including the baseline sample), thus not requiring an extra venepuncture (i.e. days 0, 2 [52 hours; 4 hrs after last dose of ivermectin], days 7, 10, 14, 21 and 28), and 6 of which are specific for the population PK study and will require an extra venepuncture (50, 54, 60, 72, 96 and 120 hours, i.e. 2, 6, 12, 24, 48, and 120 hours after the third and last dose of ivermectin). To ensure an equal distribution of samples across the different sampling time points for the extra 2 visits, participants will be divided into 4 extra sampling groups; each of which will contribute 2 extra time points, which the exception of group B which contributes 1 extra time point (Table 6). Additionally a maximum of 4 finger pricks (0.5ml whole blood) will be taken at Days 2+4h, 7, and at each of the two popPK visits. Thus the total number of samples per participant will be 13 and involve a total of 46.4 mL of whole blood (including the 7 samples for the main trial). The sampling times will be noted in the CRF, and the patient given a reminder card to return to clinic at their allocated time.

Table 6: Schedule of extra sampling points for Population PK study by 4 sampling groups

Subject Group	Sample Day * (+hours after 3rd ivermectin dose)	Sample Absolute time (hrs)*	Number per sampling strata
A	2.08 (+2h)<br>2.25 (+6h)	50<br>54	9
B	2.25 (+6h)	54	8
C	2.50 (+12h)<br>3 (+24h)	60<br>72	9
D	4 (+48h)<br>5 (+72h)	96<br>120	9
Total			35

* Extra visits that need to be made specifically for the population PK samples. The other 7 visits contributing to the population pharmacokinetic analysis (Days 0, 2, 7, 10, 14, 21, 28) coincide with the scheduled visits in the main trial. The first day is day=0; day 1 starts 24 hours after the first dose. The allocation to the sampling strata will be at random. However if a participant indicates he/she is not able to attend a certain follow-up day, the strata can be replaced by another sampling schedule (within the same allocation strata e.g. for BMI, gender etc) until all 15 or 16 allocations per sampling group have been used.

Because of an anticipated 40% refusal rate or loss to follow-up, we anticipate that the combined rich and population PK sub-studies will contribute 361 samples including 47 baseline samples (100%) and 314 (60%) follow-up samples out of a potential 524 follow-up samples across 22 sampling time points after baseline, 20 of which overlap, with a total of 12 to 47 observations per time point (Table 2, page 15).

For both rich and population PK sampling, all of the patients will be sampled by venepuncture (4 ml of whole blood to obtain 2 ml plasma), unless two consecutive samples are within 2 to 8 hours when the patient will be provided with the option to have a cannula inserted by venepuncture and the next

sample drawn from the cannula provided that it is patent after which the cannula will be removed. The patient will be asked to remain on the hospital premises or will be admitted at no cost as day patient while the cannula remains inserted. Some patients enrolled in these PK sub-studies for whom the follow-up samples will occur in the evening hours will be admitted at no cost for the duration of the PK sampling frame. In addition to the venous samples, a maximum of 4 samples will be taken concurrently by finger prick (see above in this section).

Figure 5: Overview Pharmacokinetic samples per study arm

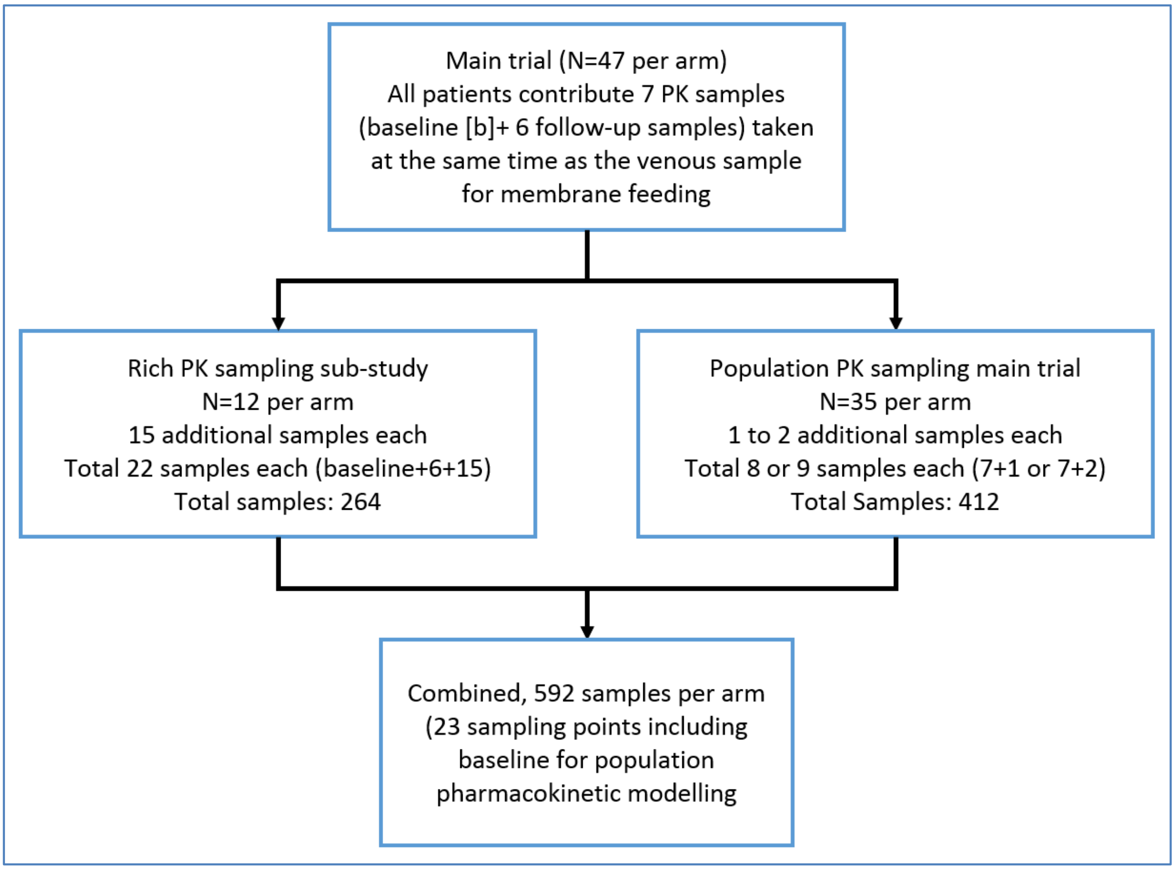

9. DATA COLLECTION, MANAGEMENT AND ANALYSIS

9.1. LABORATORY PROCEDURE

9.1.1. Mosquito colonies

See also section 9.4, Procedures for Assessing Efficacy and Safety Parameters, page 43 for use of mosquito colonies and procedures to assess the primary (mosquito survival) and secondary entomological endpoints (sporogony). This section below describes the maintenance of the mosquito colonies.

At KEMRI, we have had experience rearing mosquito colonies in our insectaries for over 10 years. The mosquito colony used in this study is the Anopheles gambiae s.s. Kisumu strain, originally from Kisumu, Kenya. The strain is susceptible to all insecticides used in public health and is free of known

metabolic and target site resistance mechanisms. When performing the membrane feeds on infected human blood, mosquitoes will be kept, and fed in cages or paper cups. The paper cups and cages will be kept in temperature and humidity controlled insectary and moved to an incubator after infectious feeds as a second line of security. The feeding and the storage of live infected mosquitoes will occur in sealed rooms with at least two doors/barriers separating the inner rooms from the outside. Mosquitoes will not be removed from their enclosures until they are killed for oocyst or sporozoite determination. If transported, live infected mosquitoes will be transported within paper cups that are enclosed within larger cages and the cages contained inside locked cool-boxes to remove any chances of escape.

Occasionally, insectary mosquitoes are shipped to Atlanta for testing to evaluate quality control and to ensure that they are free of contamination. The studies benefits from parallel membrane feeding studies of the effect of low-dose primaquine on malaria transmissibility.

9.1.2. Ivermectin plasma concentration

The lethal concentration of ivermectin able to kill 50% of exposed mosquitoes (LC50) has been estimated using spiked blood in membrane feeding essays. We will test the concentration of ivermectin in human plasma in order to provide data for a correlation and calculation of in vivo LC50 and time post-treatment that the transmission blocking effects (on mosquito survival, oocyst and sporozoites rates) lasts.

9.1.3. Hemoglobin testing

Hemoglobin will be tested using HemoCue® (Angelholm, Sweden) photometers.

9.1.4. Thick and thin blood smears for malaria

Thick and thin blood films for parasite counts will be obtained and examined. Malaria parasites will be counted against 300 white blood cells or 100 high power fields before a slide is declared negative.

9.1.5. Sero-diagnosis for Strongyloides stercoralis

We will look for the evidence of Strongyloides stercoralis infection using serodiagnosis with enzyme-linked immunosorbent assays based on crude antigen (CrAg-ELISA) or newer techniques such as the luciferase immunoprecipitation system assay (LIPS), based on a 31-kDa recombinant antigen (termed NIE) from S. stercoralis and/or the recombinant antigen S. stercoralis immunoreactive antigen (SsIR), or the NIE-ELISA have shown promise in controlled settings.[55]

9.1.6. Processing of pharmacokinetic samples

Plasma will be stored locally at site at –20° C or in liquid nitrogen and shipped to a central laboratory for storage at –70° C prior to batch analysis at the Liverpool School of Tropical Medicine / University of Liverpool. Samples will be shipped in dry ice to the laboratories in Liverpool, UK where the plasma concentrations of ivermectin and piperaquine will be determined using approved assays. Plasma concentration-time data will be used to evaluate pharmacokinetic parameters including: CL/F (oral clearance), V/F (oral volume of distribution), Ka (absorption rate constant) using population pharmacokinetic methods. Area under the curve and half-life may also be calculated.

9.2. DATA COLLECTION METHODS & STORAGE

Patient data will be collected using standardized case reporting forms on tablet computers. Laboratory and membrane feeding results will be maintained in paper laboratory books and then double entered into an Excel spreadsheet or Access database (Microsoft®. Redmond, Washington, USA). All data storage will be encrypted and password protected.

9.3. STATISTICAL METHODS

A study statistical analytical plan for the final analysis, that will supersede the study protocol, will be drawn up during the course of the study before the unblinding of data at database lock.

9.3.1. Trial profile and flowchart

A trial profile will be developed and presented as a flow chart following CONSORT guidelines, consisting of the number of participants screened, eligible, enrolled, randomized, and followed to 1 month, number contributing to primary efficacy outcomes. It will also include the number of participants who withdrew or were lost to follow-up.

9.3.2. Baseline characteristics

Descriptive statistics of baseline characteristics, overall and by treatment group will be provided in a table consisting of parameters collected prior to randomisation. No statistical comparisons will be made between the groups, but any differences between groups at baseline which are also associated with the outcome variable will be taken into account in subsequent analysis.

9.3.3. Analysis Populations

9.3.3.1. Screening failures

If a subject gives informed consent and is provided with a study ID, but then is found not to fulfil the randomisation eligibility criteria, they will be classified as a screening failure and excluded from the intention-to-treat (ITT) and the per protocol (PP) analysis.

9.3.3.2. Intention-to-treat (ITT) Population

The intention-to-treat population (the full analysis population) is defined as all patients who have received at least one dose of study medication (dosed subjects) and will be included in the intention-to-treat analysis regardless of whether they completed the last study visit.

9.3.3.3. Per protocol (PP) population

The per-protocol population is defined as all patients who took all 3 daily doses approximately (+/- 6 hours) within the 48 hour timeline, and who completed the primary endpoint visit.

9.3.3.4. Safety population

All patients who were randomized and received the first dose of study intervention and were followed up; i.e. provided information on potential adverse events.

9.3.4. Missing Data

Every effort will be made to minimise the amount of missing data in the trial. Whenever possible, information on the reason for missing data will be obtained. No adjustments will be made for missing outcome data, but missing data may be imputed for co-variates.

9.3.5. Assessment of efficacy

Primary outcomes will be summarised by number (%) of events and analysed using a Generalized Estimating Equations (GEE) model with binomial distribution and log link function that includes treatment as a single predictor taking the cluster design into account, which will generate risk ratios together with their 95% confidence intervals of having a primary endpoint between two active dose groups and the placebo group. Exchangeable covariance structure will be used. The primary endpoint analysis will be based on the ITT population. The main conclusions in the clinical report will be based on the ITT analysis of the primary outcome. An additional analysis of the primary outcome will also be performed on the per-protocol (PP) population.

Binary secondary outcomes will be analysed in a similar way as the primary endpoint. Continuous secondary outcomes will be summarised using number, mean, geometric mean, standard deviation, median, minimum and maximum and analysed using a GEE model with Gauss distribution and identity link function to derive the mean differences and 95% CI. Exchangeable covariance structure will be used.

For survival data analysis, hazard ratios and corresponding 95% confidence intervals will be calculated using Cox's regression with shared frailty to allow for the correlation between mosquito observations from the same feed. Secondary outcomes analyses will be performed on both ITT and PP populations.

9.3.6. Analysis of adverse events

Adverse reactions will be reported and tabulated for each treatment arm, overall and per body system on per protocol basis. Treatment emergent adverse events are defined as adverse events that had an onset day on or after the day of the first dose of study medication. Adverse events that have missing onset dates will be considered to be treatment emergent. No formal statistical testing will be undertaken. All laboratory data will be listed and summarised.

9.4. PROCEDURES FOR ASSESSING EFFICACY AND SAFETY PARAMETERS

9.4.1. Primary efficacy outcome

9.4.1.1. Membrane Feeding and mosquito survival

The primary outcome will be the survival of mosquitoes at 14 days after feeding on blood taken from study participants who started the 3-day ivermectin and DP regimen 7 days earlier.

The following procedures will be conducted in accordance with a standard membrane feeding protocol.[56] Mosquitoes will be fed through glass bell membrane feeding on patients' blood taken at baseline (0h), and 4h after the last dose of ivermectin and 7, 10, 14, 21, and 28 days post-ivermectin. For each feeding two new cages of 50, 3-5 day old female, insectary-reared *An. gambiae s.s.* mosquitoes will be presented to the membrane feeder for 30 minutes. The number of mosquitoes with an engorged abdomen will be counted and those with lean abdomens discarded. Each day up to day 14, the number of dead mosquitoes will be counted and removed. After the initial feeding on human blood, the mosquitoes will be kept in an incubator and maintained on sugar feeds.

9.4.1.2. Direct Skin Feeding and mosquito survival

Figure 6: Difference between membrane feeding and direct feeding

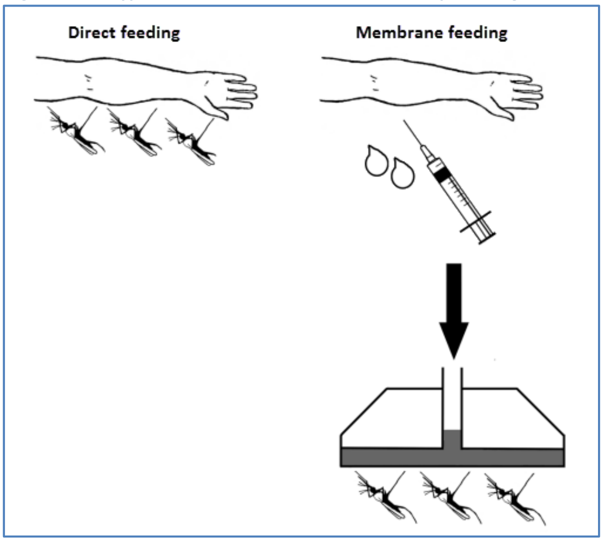

Adapted from Bousema et al 2012[51] In direct skin feeding assays, about 50 mosquitoes are placed directly on the skin of the human host. In membrane feeding assays, a venous or finger prick blood sample from the host is offered to mosquitoes. Water-jacketed glass feeders are kept at approximately 37°C and mosquitoes feed through a membrane on the whole blood taken from the host. The proportion of mosquitoes that feed within a fixed period (e.g. 10 or 15 minutes) will be recorded. The number of mosquitoes per cup will be standardized across the membrane feeding and skin feeding (e.g. 50).

9.4.2. Secondary efficacy outcomes

9.4.2.1. Daily Mosquito survival

Although the primary endpoint is assessed at day 14, the study will collect survival of mosquitoes at each day up to day 21 or 28 after each feeding experiments performed at 0, 2 day+4h, 10, 14, 21, 28 days after start of treatment. The methods will be identical to described for the primary outcome above where each day beyond day 14 the number of dead mosquitoes will be counted and removed until day 28 inclusive. The exact number of follow-up days (21 or 28 days) will be subject to logistical constraints the laboratory, and mortality rates in the mosquito populations which will be further determined prior to the start of the study. The aim is to be able to at least be able to determine the median time to mortality, which requires that at least half of the mosquito population has died in each arm. It is anticipated that 21 days will be sufficient.

9.4.2.2. Infectivity to mosquitoes

Membrane feeding: To determine mosquito infectivity in those with patent gametocytaemia (estimated to be 15 to 20% of the overall sample), 1 mL of blood will be drawn into a sodium heparinised tube and stored at 37.5° C and transported to the insectary. In the insectary, using standardized procedures, the blood will be placed into a glass bell of a membrane feeder, and a membrane will be used. Over the course of two consecutive feeds, each lasting 20 minutes, laboratory reared female Anopheles gambiae Kisumu strain mosquitoes will be fed on this blood. Separate cups will be used for oocysts, sporozoites and mosquito survival assays to allow for minimal interferences with mosquitoes after feeding and to have sufficient numbers per assay.

Assessment of oocysts and sporozoites: From day 10 post-feeding onwards, when residual DNA from the blood meal is highly unlikely,[20,57,58] all dead mosquitoes will be preserved to determine oocyst and sporozoite prevalence by Elisa or by polymerase chain reaction (PCR). Mosquitoes will be individually homogenized and processed, first in pooled batches, and then in smaller batches and eventually individually if the batch was found to be positive for oocysts or sporozoites. The exact number of

mosquitoes per feed (likely 100 as 2x50 per feed) will be determined in the insectary at Kisian prior to initiation of the study, and the findings will be standardized throughout the study. If inadequate funding is available for PCR for both oocysts and sporozoites, preference will be given to assessment of oocysts. If inadequate funding for PCR is available, the presence of oocysts will be assessed by microscopy in dissected mosquitoes on samples taken between 7 to 10 days post-feeding.

9.4.2.3. Asexual treatment response and parasite clearance

Standard methods will be used to assess the in-vivo treatment response to DP using the microscopy and RDT data collected at each scheduled follow-up visit and criteria described by WWARN.[59]

9.4.3. Safety outcomes

9.4.3.1. Pupillometry

Pupil diameter size will be measured at baseline and each schedule visit using a portable, single-button activation, battery operated hand-held pupillometry device which very accurately measures pupil size requiring no calibration (such as the Neuroptics VIP™-200 Variable Pupillometer, which measures the pupil 30 times per second over a two-second period and provides the average pupil diameter and standard deviation (+/- 0.1 mm).

9.4.3.2. ECG monitoring

We will do a 12-lead ECGs to measure the QTc interval at baseline, and Day 2 pre-last dose, Day 2 at 4-6h post-last dose and again at Day 28. The day 28 sample is included as a true baseline is difficult to assess in patients with acute malaria as malaria and fever are known to increase the heart rate and decrease the QTc interval. On day 28 most, if not all, patients, will be malaria free and residual piperaquine levels low enough not to affect QTc intervals.

A portable ECG machine will be used with automated ECG interpretation. All automated outputs will be verified manually in a blinded manner. Patients with a QTc value of 500 ms or greater prior to the last dose of DP will not receive the last dose of DP, but receive a full course of artemether lumefantrine instead. Fridericia's correction will be used to calculate the QTc values for final data analysis ($QTc = QT/RR^{0.33}$).

9.5. MONITORING

9.5.1. Data Monitoring

9.5.1.1. Data Monitoring and Ethics Committee (DMEC)

Since the study is a clinical trial an independent Data Monitoring and Ethics Committee (DMEC) will be set up. The DMEC will be critical to ensure that the subjects are protected from harm, while also ensuring that the study integrity is not compromised. The DMEC will consist of 3 independent members knowledgeable in the conduct of clinical trials. They will meet regularly (e.g. twice yearly or more frequent if so required) during data collection period to provide a review of blinded (and if requested unblinded) data to ensure the safety, rights and well-being of trial participants. The trial statistician could also be asked to attend the meetings. The role and membership of the DMEC is described in more detail in Appendix II. Terms of Reference Oversight committees, page 65.

9.5.1.2. Interim analyses and criteria for termination of the trial

An interim analyses of the required sample size for efficacy and safety data will be conducted when 50% of participants have completed 14 days follow-up. The DMEC will remain blinded when presented with the interim analysis, unless the DMEC judges that for safety reasons the study blind should be broken. A detailed plan for interim analysis, any planned statistical adjustments to be employed as a result of interim analysis, the provisional stopping rules and how the stopping rules will be applied, will be drawn up prior to the start of the interim analysis and documented in the study statistical analysis plan.

In addition, regular review of the quality of the study data will be conducted at each meeting of the DMEC.

The sponsor reserves the right to temporarily suspend or prematurely discontinue this study at any time for reasons including, but not limited to, safety or ethical issues or severe non-compliance. If the sponsor determines such action is needed, it will discuss this with the investigator. When feasible, the sponsor will provide advance notification to the investigator of the impending action prior to it taking effect. The sponsor will promptly inform the IEC/IRB and provide the reason for the suspension or termination.

9.5.1.3. An interim analysis for continuation of direct feeding

An additional interim analysis will be conducted after the first 27 patients enrolled in the first feeding assay sub-study. The results will be presented to the DMEC who will be requested to look at the results after unblinding the individual allocation codes for these 24 patients (i.e. without having to break the master code for the entire trial), and advice whether the results justify the use of continued direct feeding on day 7 in the remaining of the patients.

9.6. SAFETY MONITORING AND REPORTING

The principles of ICH GCP require that both investigators and sponsors follow specific procedures when notifying and reporting adverse events or reactions in clinical trials.

9.6.1. Definitions

The following definitions apply to this protocol:

9.6.1.1. Adverse Event (AE)

Any untoward medical occurrence in a patient or clinical trial subject administered a medicinal product and which does not necessarily have a causal relationship with this treatment.

9.6.1.2. Adverse Reaction (AR)

Any untoward and unintended response to an investigational medicinal product related to any dose administered.

Comment: All adverse events judged by either the reporting investigator or the sponsor as having a reasonable causal relationship to a medicinal product would qualify as adverse reactions. The expression ‘reasonable causal relationship’ means to convey, in general, that there is evidence or argument to suggest a causal relationship.

9.6.1.3. Serious Adverse Event (SAE) or Serious Adverse Reaction (SAR)

Any adverse event or adverse reaction that results in death, is life-threatening*, requires hospitalisation or prolongation of existing hospitalisation, results in persistent or significant disability or incapacity, or is a congenital anomaly or birth defect.

Comment: Medical judgement should be exercised in deciding whether an adverse event/reaction should be classified as serious in other situations. Important adverse events/reactions that are not immediately life-threatening or do not result in death or hospitalisation, but may jeopardise the subject or may require intervention to prevent one of the other outcomes listed in the definition above, should also be considered serious.

*Life-threatening in the definition of a serious adverse event or serious adverse reaction refers to an event in which the subject was at risk of death at the time of the event. It does not refer to an event which hypothetically might have caused death if it were more severe.

9.6.1.4. Suspected Unexpected Serious Adverse Reaction (SUSAR)

An adverse reaction that is both unexpected (not consistent with the applicable product information) and also meets the definition of a Serious Adverse Event/Reaction.

9.6.1.5. Intensity

The intensity of each AE recorded in the case report form should be assigned to a grade (1-5), which will be determined following the definitions set forth in the Common Terminology Criteria for Adverse Events v3.0 (CTCAE) (Cancer Therapy Evaluation Program, 2006). Use of these standardized guidelines will allow for uniform reporting. The grades are defined as follows:

- Grade 1: Mild AE
- Grade 2: Moderate AE
- Grade 3: Severe AE
- Grade 4: Life-threatening or disabling AE
- Grade 5: Death related to AE

9.6.2. Identifying, managing adverse events

Participants who develop adverse events as a consequence of ivermectin treatment or other treatments will be identified at follow-up visits and referred to the designated hospital for evaluation and treatment according to local guidelines. Mild adverse events will be noted in the participant's case report form; no further action will be taken by study staff except in the case of vomiting, in which case the study medication may need to be re-administered. In the case of any severe adverse event (difficulty breathing, convulsions, change in mental status), subjects will be referred to the hospital for management. Transportation to the hospital will be provided. All hospitalized participants will undergo record review to identify potential adverse consequences of study participation.

9.6.3. Assessment of Causality

The investigator is obligated to assess the relationship between investigational product and the occurrence of each AE/SAE. The investigator will use clinical judgment to determine the relationship. Alternative causes, such as natural history of the underlying diseases, concomitant therapy, other risk factors, and the temporal relationship of the event to the investigational product will be considered

and investigated. The investigator will also consult the drug information and the DMEC as needed in the determination of his/her assessment.

There may be situations when an SAE has occurred and the investigator has minimal information to include in the initial report (see 9.6.4). However, it is very important that the investigator always make an assessment of causality for every event prior to transmission of the SAE report to KEMRI and LSTM. The investigator may change his/her opinion of causality in light of follow-up information, amending the SAE case report form accordingly.

9.6.4. Reporting adverse event procedures

All SAEs will be reported to the in country principal investigator or an assigned representative within 24 hours of the staff becoming aware of it, using an SAE form, which should be completed, scanned and sent electronically. The SAE form asks for nature of event, date of onset, severity, corrective therapies given, outcome and causality (i.e. unrelated, unlikely, possible, probably, definitely). The responsible study clinician should assign the causality of the event.

9.6.4.1. Expedited reporting

SAEs that are unexpected and are at least 'possibly related' to the study drug require expedited reporting within 24 hours of the country principal investigator or assigned representative becoming aware of it (e-mail notification); i.e. this will be a maximum of 48 hours after the event occurred (including the 24 hours required for the field staff to report to the principal investigator / representative). Additional information will be sent within 14 additional days (full SAE report) if the reaction had not resolved at the time of e-mail notification.

9.6.4.2. Monthly reporting

Other SAEs and AEs will be reported annually in an aggregated report. AEs that will not be reported include common illnesses that do not result in hospitalization, including but not limited to clinical malaria, respiratory, gastrointestinal, and skin diseases, unless they are considered at least possibly related to the intervention.

9.6.4.3. Recipients of reports

The study will comply with local regulations pertaining to reporting of SAEs to their local Research Ethics Committee and/or Research & regulatory offices. In addition to the primary ethics committees, we will report safety data to the DMEC, the sponsor and to manufacturers of ivermectin and DP. A copy of the final study report will be provided to all RECs, DMEC, local regulator, and the manufacturers of ivermectin and DP.

Figure 7: Safety reporting assessment flowchart[60]

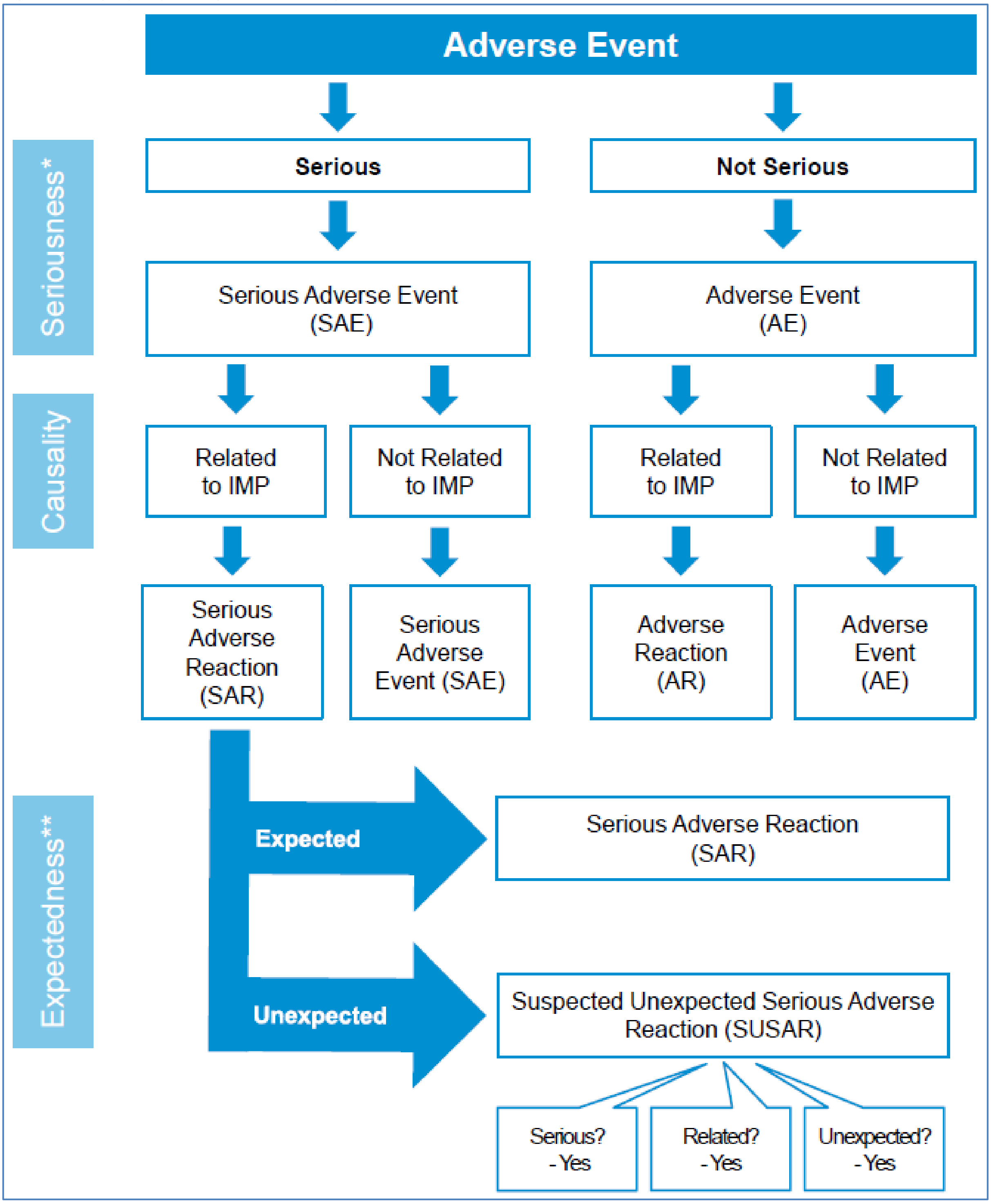

IMP: Investigational Medicinal Product

*See definition of SAE in section 9.6.1.3, page 47

**Assessed in line with the current approved Investigator's Brochure (IB)

9.7. QUALITY ASSURANCE

9.7.1. *Clinical monitoring and auditing*

9.7.1.1. Clinical monitoring

9.7.2. *Clinical Monitoring*

Monitoring of this trial will be conducted to ensure compliance with Good Clinical Practice and scientific integrity will be managed and oversight retained, by the sponsor. Clinical monitoring will be sub-contracted to an independent clinical monitor. A total of 3 monitoring visits will be carried out; one at trial initiation, one at close-out and one 3 month after the start or half-way the study.

Prior to subject enrolment, the monitor will visit the study site to determine the adequacy of facilities, review the protocol and data collection procedures and discuss the responsibilities of the investigator and other study site personnel.

During the study, the monitor will have regular site contacts, including conducting on-site visits to:

1. Confirm that the study is being performed according to the protocol, ICH GCP and applicable regulations, data are being accurately recorded in the CRFs and that investigational product accountability is being performed.
2. Conduct source data verification
3. Confirm facilities remain acceptable
4. Provide information and support to the investigators
5. Evaluate study progress

Upon completion of the study the monitor will visit the study site to verify that all CRFs are completed and collected, all data queries have been resolved and filed, conduct final accountability, reconciliation and arrangements for investigational product and verify all study site records are complete.

The PI and relevant staff will be available at monitoring visits and agree to allocate sufficient time to the monitor to discuss any issues and address their resolution.

9.7.2.1. Auditing

The independent clinical monitoring process will be audited by a study staff from the sponsor’s research office at LSTM in Liverpool, UK. The auditor will accompany the clinical monitor during at least one of the site visits. After this visit it will be determined by the sponsor if more auditing visits are required.

9.7.3. *Training*

The principal Investigators are responsible for the conduct of the study at the study sites, including delegation of specified study responsibilities, and training of study staff. The PIs will maintain a record of all individuals involved in the study (medical, nursing and other staff) and will ensure that all persons assisting with the trial receive the appropriate training about the protocol, the investigational product(s) and their trial-related duties and functions, including formal certified GCP training. During the study the regular spot checks will be conducted to assess the performance of study site staff members and re-training provided where necessary.

9.7.4. Quality assurance/control of laboratory tests

Regular audits of laboratory performance will be completed by experienced supervisors according to standard operating procedures. All malaria blood smears will be read by two different microscopists; any significantly discordant results based on positive/negative results or difference in parasites above a defined threshold will be verified by a third expert microscopist.

10. TIMEFRAME AND DURATION OF THE STUDY

The anticipated start date for enrolment is March 2015.

Activity	M1	M2	M3	M4	M5	M6	M7	M8	M9	M10	M11	M12
Protocol development												
IRB Review												
Pre-testing in the insectary												
Enrolment												
Study												
Data analysis												
Manuscript preparation												

11. ETHICAL CONSIDERATIONS & REGULATORY APPROVALS

11.1. DECLARATION OF HELSINKI

The trial will be conducted in compliance with the principles of the Declaration of Helsinki (1996) (See section 16.3, "Appendix III. Declaration of Helsinki", page 68), the principles of GCP and in accordance with all applicable regulatory requirements in Uganda and Kenya.

11.2. REGULATORY APPROVAL AND TRIAL AUTHORISATION

Since the trial is conducted outside the EU, no authorisation from a European regulator is required. Trial authorisation will be sought from the Kenyan regulator (the Kenyan Pharmacy & Poison Board).

11.3. RESEARCH ETHICS APPROVAL

11.3.1. Review Process

This protocol, the informed consent document, patient information sheets will be reviewed and approved by the Research Ethics Committees at KEMRI, Nairobi, Kenya (primary committee), and the Liverpool School of Tropical Medicine, Liverpool (LSTM).

11.3.2. Protocol amendments

No change will be made to the approved protocol without the agreement of the sponsor.

If it is necessary for the protocol to be amended, the protocol amendment will be submitted to the primary ethics committee for approval before implementation. Any protocol amendments will be submitted to the primary ethics committees (KEMRI) before implementation. Any change to the informed consent form must also be approved by the sponsor and the primary ethics committee in each country IRB/IEC, before the revised form is used.

The sponsor will distribute amendments to each principal investigator, who in turn is responsible for the distribution of these documents to the staff at his/her study site.

11.4. INFORMED CONSENT PROCEDURES

11.4.1. Consent procedures

Written, informed consent will be obtained in the local language. The consent process shall be initiated at the time of enrolment into the study and shall continue throughout the patient's participation. Patients meeting the initial eligibility criteria (adults with symptomatic malaria) will have the study explained to them by a member of the study team. If the patient meets the study enrolment criteria, the full consent process will follow, with a written consent form provided.

For illiterate participants, an independent witness will be present during the informed consent process and will sign the consent form as a witness, while the participant will be asked to indicate consent by use of thumbprint. The participant may withdraw consent at any time throughout the course of the study, and this will be made clear in the informed consent process. A copy of the informed consent document will be given to the caregiver for their records, unless they state that they do not wish to have a copy. All individuals will be informed that there is no requirement to join the study and that standard medical care will remain the same regardless of study enrolment.

If the patient chooses not to be enrolled in the study, the case will be turned over to the attending physician currently on duty for routine care of their condition.

11.4.2. Consent forms

There will be three consent forms for:

1. The main trial
2. The direct feeding sub-study.
3. The nested rich pharmacokinetic study

The main trial consent form will include two parts. This first part gives permission for all study related procedures, including copying all relevant information from the hospital and clinic records and laboratory registries and the collection of biological samples. The second part will give permission for long-term storage of the blood for future studies as well as for genetic studies on the patient's blood sample related to malaria and drug metabolism. For this test the samples shall be stored as frozen venous blood samples and in filter papers and shipped for analysis to the respective laboratories overseas.

All consent forms will be translated into both Kiswahili and Dholuo and back translated into English to ensure accuracy.

11.5. PROTECTION OF PRIVACY AND CONFIDENTIALITY

11.5.1. Privacy

Personal and medical information relating to research participants will be treated as confidential. The risk of disclosure will be minimized by secure storage of documents and use of linked data by replacing personal identifiers with a unique study code to conceal the identity of the patient.

11.5.2. Privacy of individual

Individual data such as tests for malaria and anaemia will be reported to the participant at point of care, to relevant study staff and where appropriate will be recorded in the patients' medical record book in addition to study CRFs.

11.5.3. Confidentiality of data

All information regarding the participants will remain confidential to the extent allowed by law. Unique numerical identifiers will be used for data entry. All screening forms and case report forms will be kept in a secured location with access limited to authorized study staff. Unique numerical identifiers will be used for the computer-based data entry and blood samples. Publications will contain only aggregate data. No identifying information will be included.

11.6. DECLARATION OF INTEREST

None of the principal investigators have paid consultancies with the pharmaceutical companies involved in the trial, or other competing interest for the overall trial or in each study site.

11.7. ACCESS TO SOURCE DATA/DOCUMENTS

In addition to the clinical monitors, authorised representatives of the sponsor/CRO, an IEC/IRB or regulatory authority may visit the study site to perform audits or inspections, including source data verification. The investigator agrees to allow the sponsor and CRO representatives, including the monitor and study safety monitor, the DMEC, the IRB/IEC direct access to source data and other relevant documents.

11.8. RISKS AND BENEFITS

11.8.1. Risks to Study Participants

11.8.1.1. Ivermectin

Ivermectin has been shown to be well tolerated with doses up to 2000 mcg/kg; i.e. up to double the Cmax that is anticipated with the highest proposed dose (600 mcg/kg/day for 3 days) that will be used in the trial and safe, including during pregnancy (see Section 4.6 "Safety of ivermectin in humans", page 19). The only known severe adverse events have been in individuals with *Loa loa* due to lysis of parasites, however *Loa loa* is not present in Kenya. In an onchocerciasis study in Cameroon, transitory mild visual side effects were seen without structural abnormalities upon ophthalmological exam.

11.8.1.2. Dihydroartemisinin-piperaquine

DP is currently the second-line antimalarial in Kenya, but is the first-line antimalarial in many Asian countries.

Artemisinin derivatives are well tolerated, and are both the first and second line antimalarials (except in women who are pregnant in the first trimester) in Kenya. There are rare reports of urticarial reactions.

Piperaquine is also generally well tolerated, but may cause dizziness, headaches, nausea, vomiting, abdominal pain or diarrhoea. The main safety concerns with piperaquine relate to its dose-dependent QTc prolongation. Transient QTc prolongation has been confirmed in clinical trials, but these were mild and similar to many other anti-malarials[61] and there is no indication from clinical data signalling that it is associated with clinically significant arrhythmias.[61,62,63] This is consistent with recent in-vitro models which confirmed that despite mild QTc prolongation, the potential cardiac proarrhythmic risk with piperaquine is low and similar to that observed with lumefantrine (the long-acting component in Coartem), and lower than for chloroquine. This study concluded that DP does not appear to induce potential torsadogenic effects in vitro (which could result in life threatening abnormality of heart rhythm).

Nevertheless, this study is designed to exclude a drug interaction between DP and ivermectin, thus in addition to drug levels, all patients will be monitored for QTc prolongation and the dose of DP discontinued if the QTc interval exceeds 500 ms.

All subjects will be monitored for adverse events, and will have complete clinical histories, with particular attention to known side-effects of the study drugs, and physical examinations performed at every visit. In particular, participants will be referred for ophthalmological consultation should they experience any ophthalmological side-effects. Any adverse events incurred as a direct result of study participation will be treated free of charge.

11.8.1.3. Blood sampling

Blood sampling may be inconvenient to the participants, and may cause minor discomfort and bruising and local infection if not conducted adequately. The volume of blood collected from each participant will be not more than 5.7 mL each time and a 98.4 ml in total in the standard pharmacokinetic group, and 46.4 mL in the remaining patients. Well-trained clinicians, nurses and laboratory staff employed on the trial will perform blood-sampling tasks. New and sterile disposable needles and lancets will used for blood sample collection. Universal precaution measures for blood handling and disposal will be observed when performing the procedures and used needles and other waste will be safely discarded immediately after use.

11.8.1.4. Direct skin feeding

Direct skin feeding may be considered unpleasant by the participant. Secondary infection of the mosquito bites in the patients taking part in the direct skin feeding experiments will be monitored at each subsequent scheduled and unscheduled follow-up visit and treated appropriately.

11.8.2. Benefits to study participants

11.8.2.1. Anticipated benefits to study participants

Ivermectin is an antihelminth drug and may be beneficial to patients with helminth infections and other endoparasites and ectoparasites. All patients will receive treatment for malaria, helminths, scabies, bedbugs and lice, free of charge. Patients will also be provided with a superior level of supervision and monitoring than they otherwise would have been.

11.8.2.2. Benefit to the community

This project is designed to generate the information required to determine whether ivermectin can be considered as adjunct therapy in MDA for malaria transmission reduction. As such the study helps support science and potential progress towards enhanced options in the arsenal of tools for malaria control and elimination. Subject to the finding, in the longer term, the ultimate beneficiaries of this research could be the populations living in malaria endemic parts of the world, whose quality of life, health, welfare and creative output will be enhanced by reduced malaria transmission.

11.9. ANCILLARY AND POST-TRIAL CARE

11.9.1. Health care during the trial

All care directly related to the proper and safe conduct of the trial, and the treatment of immediate adverse events related to trial procedures will be provided free of charge by the study in the study hospitals. The provision of ancillary care beyond that immediately required for conduct of the trial will not be covered by the trial.

11.9.2. Trial insurance

The sponsor will take out trial insurance such that participants enrolled into the study are covered by indemnity for negligent harm and non-negligent harm associated with the protocol. This will include cover for additional health care, compensation or damages whether awarded voluntarily by the Sponsor, or by claims pursued through the courts. The liability of the manufacturer of the trial drug ivermectin and DP is limited to those claims arising from faulty manufacturing of the commercial product and not to any aspects of the conduct of the study.

11.9.3. Post-trial care

The study budget is not in a position to fund post-study care or implementation of ivermectin as policy. However, the investigators work in close collaboration with local and international policy makers (e.g. WHO) and funders (e.g. President Malaria Initiative) to ensure that policy makers and funders are informed early of germane research findings.

11.10. EXPENSES REIMBURSEMENT AND INCENTIVES

The study will provide payment for all study drugs, study procedures, study-related visits and reasonable medical expenses that are incurred in study clinics or hospitals as a result of the study, including expenses for transport for any study related visits including unscheduled visits in between scheduled visits to study clinics. The study will not cover the costs of any non-malaria or non-study related events, including scheduled or unscheduled surgery or trauma related events (e.g. accidents,

burns etc.) if this is not deemed to be related to the study by the principal investigators or their representative.

Table 7: Reimbursement of expenses and incentives provided by the study

To Who	What	Approximate Amount
Hospital	Improvement of infrastructure where required	~$5,000/hospital
	Training of routine staff adult ward	~ $2,000/hospital
	Study procedure costs and study drugs and admission fees for inpatients	~ $70,000/hospital
Patient	Travel expenses for participant as per KEMRI guidelines*	Up to ~$3.5-5.5 (300-500 Ksh per round trip based on distance as per KEMRI guidelines
	Compensation for each day they are scheduled to come to the out-patient clinic and need to stay or wait for the more than 4 hours (e.g. because of a repeat blood sample that has to be taken a fixed number of hours after the dose of ivermectin)	$ ~2.3 (200 Ksh) per day (excluding any travel expenses)
	Compensation for each day that the patient is admitted in the hospital (Rich Pk sub-study).	$ ~5.5 (500 Ksh) per day + $3.5 to $5.5 (300-500) Ksh per day travel expenses for family/spouse
	Meal or reimbursement for breakfast, lunch or dinner for the participant and any accompanying if required to stay for more than half a day (lunch) or overnight (breakfast and dinner), as per KEMRI guidelines	Up to $ ~2.8 (about 250 Ksh)) per person per meal

*The amounts of Ksh 300 to 500 for travel and Ksh 500 reimbursement for each day that they are hospitalized and/or are scheduled to come for a visit has been established after discussions with KEMRI Ethical Review Committee and is deemed as fair compensation for money spent to travel in Kisumu city to the hospital from the catchment areas.

12. DISSEMINATION AND APPLICATION OF THE RESULTS

12.1. RESULT DISSEMINATION AND PUBLICATION POLICY

At the end of the trials, the results will first be disseminated to national policy makers, government departments, academics from local research institutions and universities, and professional bodies in Kenya at the national stakeholders' meeting. Subject to the findings of the study and based on consensus emerging at these meetings, project partners in Kenya will support national and international policy makers to in deciding whether there is a role for ivermectin in malaria control.

Research results will also be disseminated to the global malaria research community, technical agencies, and international government bodies via peer reviewed journals and at international scientific fora, including the annual American Society of Tropical Medicine and Hygiene (ASTMH) meeting, and via meetings at WHO in Geneva comprised of leading scientists in the field of malaria.

We will also inform other international organisations and funders of large scale malaria control initiatives including DFID, USAID and the US President's Malaria Initiative (PMI) which aim to improve malaria at regional and local levels and are instrumental in supporting countries to implement malaria control policies in Africa.

12.2. IMPACT

New strategies for malaria control, and eventually towards elimination are critically needed. This protocol explores a research and programmatic question that is currently highly discussed. In addition to the other known and planned studies occurring in other malaria endemic countries, this protocol will seek to answer the question as to whether it will be feasible to introduce ivermectin for the control of malaria in Kenya, including for case management of symptomatic malaria and for adjunct therapy in MSaT or MDA campaign. We expect the results of this protocol to inform national malaria control programs in malaria endemic countries, to inform WHO guidelines, to be published in high-profile peer-reviewed journals, to be presented at domestic and international conferences, and the data to be shared with other groups for meta-analysis.

12.3. TRAINING, FELLOWSHIPS AND CAPACITY BUILDING

Research capacity in research partner institutes in Kenya will be enhanced by provision of training and mentorship for clinic and research staff. By running this trial, capacity in trial management will be enhanced. The research study will strengthen the clinical skills of health workers in managing patients. There will be 1 PhD candidate (MS) who will conduct his research as part of this project. Partners from KEMRI and LSTM Liverpool will jointly act as supervisors.

In addition, Kenyan laboratory scientist/staff from KEMRI will have the opportunity to learn new assay and analytical techniques in pharmacokinetics (of piperaqine and ivermectin) at the LSTM in Kenya.

12.4. AUTHORSHIP AND PUBLICATIONS

The study will have a publications committee consisting of the two PIs (MS and EO) and Chief Investigator (FtK). Potential authors include all professionals that have participated in the trial for a minimum of 4 months. Authorship of any presentations or publications arising from this study will also be governed by the principles for authorship criteria of the International Committee of Medical Journal Editors has designed.[64] Disputes regarding authorship will be settled by the publications committee, with further involvement of the independent chair of the TSC if so required. The manufacturer of the study medication will be provided with a draft of the manuscript but will have no role in review, data interpretation, or writing of the article.

12.5. DATA SHARING STATEMENT

The full protocol will be available on request to any interested professional and may be published in a peer reviewed journals or deposited in an online repository. Individual, de-identified participant data

will be made available for meta-analyses as soon as the data analysis is completed, with the understanding that results of the meta-analysis will not be published prior to the results of the individual trial without prior agreement of the investigators. No later than 5 years after the publication of the trial a fully de-identified data set will be available for sharing purposes. All requests for data for secondary analysis will be considered by the publication committee.

13. REFERENCES

1. Hamel MJ, Adazu K, Obor D, Sewe M, Vulule J, Williamson JM, Slutsker L, Feikin DR, Laserson KF, 2011. A reversal in reductions of child mortality in western Kenya, 2003-2009. Am J Trop Med Hyg 85: 597-605.
2. Beier JC, Perkins PV, Onyango FK, Gargan TP, Oster CN, Whitmire RE, Koech DK, Roberts CR, 1990. Characterization of malaria transmission by Anopheles (Diptera: Culicidae) in western Kenya in preparation for malaria vaccine trials. J Med Entomol 27: 570-7.
3. Sudoi RK, Githinji S, Nyandigisi A, Muturi A, Snow RW, Zurovac D, 2012. The magnitude and trend of artemether-lumefantrine stock-outs at public health facilities in Kenya. Malar J 11: 37.
4. Sanitation; MoPHa, 2011. 2010 Kenya Malaria Indicator Survey.
5. Mathias DK, Ochomo E, Atieli F, Ombok M, Bayoh MN, Olang G, Muhia D, Kamau L, Vulule JM, Hamel MJ, Hawley WA, Walker ED, Gimnig JE, 2011. Spatial and temporal variation in the kdr allele L1014S in Anopheles gambiae s.s. and phenotypic variability in susceptibility to insecticides in Western Kenya. Malar J 10: 10.
6. Morgan JC, Irving H, Okedi LM, Steven A, Wondji CS, 2010. Pyrethroid resistance in an Anopheles funestus population from Uganda. PLoS One 5: e11872.
7. McCann RS, Ochomo E, Bayoh MN, Vulule JM, Hamel MJ, Gimnig JE, Hawley WA, Walker ED, 2014. Reemergence of Anopheles funestus as a vector of Plasmodium falciparum in western Kenya after long-term implementation of insecticide-treated bed nets. Am J Trop Med Hyg 90: 597-604.
8. Chaccour CJ, Kobylinski KC, Bassat Q, Bousema T, Drakeley C, Alonso P, Foy BD, 2013. Ivermectin to reduce malaria transmission: a research agenda for a promising new tool for elimination. Malar J 12: 153.
9. Gonzalez P, Gonzalez FA, Ueno K, 2012. Ivermectin in human medicine, an overview of the current status of its clinical applications. Curr Pharm Biotechnol 13: 1103-9.
10. 2012. Mectizan Donation Program: Annual Highlights.
11. Ejere HO, Schwartz E, Wormald R, Evans JR, 2012. Ivermectin for onchocercal eye disease (river blindness). Cochrane Database Syst Rev 8: Cd002219.
12. Shmidt E, Levitt J, 2012. Dermatologic infestations. Int J Dermatol 51: 131-41.
13. Foy BD, Kobylinski KC, da Silva IM, Rasgon JL, Sylla M, 2011. Endectocides for malaria control. Trends Parasitol 27: 423-8.
14. Butters MP, Kobylinski KC, Deus KM, da Silva IM, Gray M, Sylla M, Foy BD, 2012. Comparative evaluation of systemic drugs for their effects against Anopheles gambiae. Acta Trop 121: 34-43.
15. Chaccour C, Lines J, Whitty CJ, 2010. Effect of ivermectin on Anopheles gambiae mosquitoes fed on humans: the potential of oral insecticides in malaria control. J Infect Dis 202: 113-6.
16. Kobylinski KC, Deus KM, Butters MP, Hongyu T, Gray M, da Silva IM, Sylla M, Foy BD, 2010. The effect of oral anthelmintics on the survivorship and re-feeding frequency of anthropophilic mosquito disease vectors. Acta Trop 116: 119-26.
17. Sylla M, Kobylinski KC, Gray M, Chapman PL, Sarr MD, Rasgon JL, Foy BD, 2010. Mass drug administration of ivermectin in south-eastern Senegal reduces the survivorship of wild-caught, blood fed malaria vectors. Malar J 9: 365.

18. Kobylinski KC, Sylla M, Chapman PL, Sarr MD, Foy BD, 2011. Ivermectin mass drug administration to humans disrupts malaria parasite transmission in Senegalese villages. Am J Trop Med Hyg 85: 3-5.
19. Kobylinski KC, Foy BD, Richardson JH, 2012. Ivermectin inhibits the sporogony of Plasmodium falciparum in Anopheles gambiae. Malar J 11: 381.
20. Ouedraogo AL, Bastiaens GJ, Tiono AB, Guelbeogo WM, Kobylinski KC, Ouedraogo A, Barry A, Bougouma EC, Nebie I, Ouattara MS, Lanke KH, Fleckenstein L, Sauerwein RW, Slater HC, Churcher TS, Sirima SB, Drakeley C, Bousema T, 2014. Efficacy and Safety of the Mosquitocidal Drug Ivermectin to Prevent Malaria Transmission After Treatment: A Double-Blind, Randomized, Clinical Trial. Clin Infect Dis.
21. Fritz ML, Siegert PY, Walker ED, Bayoh MN, Vulule JR, Miller JR, 2009. Toxicity of bloodmeals from ivermectin-treated cattle to Anopheles gambiae s.l. Ann Trop Med Parasitol 103: 539-47.
22. malERA Consultative Group on Drugs, 2011. A research agenda for malaria eradication: drugs. PLoS Med 8: e1000402.
23. Foley DH, Bryan JH, Lawrence GW, 2000. The potential of ivermectin to control the malaria vector Anopheles farauti. Trans R Soc Trop Med Hyg 94: 625-8.
24. Alout H, Krajacich BJ, Meyers JI, Grubaugh ND, Brackney DE, Kobylinski KC, Diclaro JW, 2nd, Bolay FK, Fakoli LS, Diabate A, Dabire RK, Bougma RW, Foy BD, 2014. Evaluation of ivermectin mass drug administration for malaria transmission control across different West African environments. Malar J 13: 417.
25. Slater HC, Walker PG, Bousema T, Okell LC, Ghani AC, 2014. The Potential Impact of Adding Ivermectin to a Mass Treatment Intervention to Reduce Malaria Transmission: a Modelling Study. J Infect Dis.
26. Omura S, 2008. Ivermectin: 25 years and still going strong. Int J Antimicrob Agents 31: 91-8.
27. Fox LM, 2006. Ivermectin: uses and impact 20 years on. Curr Opin Infect Dis 19: 588-93.
28. White AT, Newland HS, Taylor HR, Erttmann KD, Keyvan-Larijani E, Nara A, Aziz MA, D'Anna SA, Williams PN, Greene BM, 1987. Controlled trial and dose-finding study of ivermectin for treatment of onchocerciasis. J Infect Dis 156: 463-70.
29. Burgess IF, 2011. Head lice. Clin Evid (Online) 2011.
30. Chosidow O, Giraudeau B, Cottrell J, Izri A, Hofmann R, Mann SG, Burgess I, 2010. Oral ivermectin versus malathion lotion for difficult-to-treat head lice. N Engl J Med 362: 896-905.
31. Pacque M, Munoz B, Poetschke G, Foose J, Greene BM, Taylor HR, 1990. Pregnancy outcome after inadvertent ivermectin treatment during community-based distribution. Lancet 336: 1486-9.
32. Chippaux JP, Gardon-Wendel N, Gardon J, Ernould JC, 1993. Absence of any adverse effect of inadvertent ivermectin treatment during pregnancy. Trans R Soc Trop Med Hyg 87: 318.
33. Doumbo O, Soula G, Kodio B, Perrenoud M, 1992. [Ivermectin and pregnancy in mass treatment in Mali]. Bull Soc Pathol Exot 85: 247-51.
34. Gyapong JO, Chinbuah MA, Gyapong M, 2003. Inadvertent exposure of pregnant women to ivermectin and albendazole during mass drug administration for lymphatic filariasis. Trop Med Int Health 8: 1093-101.
35. Ndyomugyenyi R, Kabatereine N, Olsen A, Magnussen P, 2008. Efficacy of ivermectin and albendazole alone and in combination for treatment of soil-transmitted helminths in pregnancy and adverse events: a randomized open label controlled intervention trial in Masindi district, western Uganda. Am J Trop Med Hyg 79: 856-63.
36. Brown KR, 1998. Changes in the use profile of Mectizan: 1987-1997. Ann Trop Med Parasitol 92 Suppl 1: S61-4.
37. Awadzi K, Opoku NO, Addy ET, Quartey BT, 1995. The chemotherapy of onchocerciasis. XIX: The clinical and laboratory tolerance of high dose ivermectin. Trop Med Parasitol 46: 131-7.

38. Awadzi K, Attah SK, Addy ET, Opoku NO, Quartey BT, 1999. The effects of high-dose ivermectin regimens on Onchocerca volvulus in onchocerciasis patients. Trans R Soc Trop Med Hyg 93: 189-94.
39. Guzzo CA, Furtek CI, Porras AG, Chen C, Tipping R, Clineschmidt CM, Sciberras DG, Hsieh JY, Lasseter KC, 2002. Safety, tolerability, and pharmacokinetics of escalating high doses of ivermectin in healthy adult subjects. J Clin Pharmacol 42: 1122-33.
40. Kamgno J, Gardon J, Gardon-Wendel N, Demanga N, Duke BO, Boussinesq M, 2004. Adverse systemic reactions to treatment of onchocerciasis with ivermectin at normal and high doses given annually or three-monthly. Trans R Soc Trop Med Hyg 98: 496-504.
41. Fobi G, Gardon J, Kamgno J, Aimard-Favennec L, Lafleur C, Gardon-Wendel N, Duke BO, Boussinesq M, 2005. A randomized, double-blind, controlled trial of the effects of ivermectin at normal and high doses, given annually or three-monthly, against Onchocerca volvulus: ophthalmological results. Trans R Soc Trop Med Hyg 99: 279-89.
42. Gardon J, Gardon-Wendel N, Demanga N, Kamgno J, Chippaux JP, Boussinesq M, 1997. Serious reactions after mass treatment of onchocerciasis with ivermectin in an area endemic for Loa loa infection. Lancet 350: 18-22.
43. Wanji S, Akotshi DO, Mutro MN, Tepage F, Ukety TO, Diggle PJ, Remme JH, 2012. Validation of the rapid assessment procedure for loiasis (RAPLOA) in the democratic republic of Congo. Parasit Vectors 5: 25.
44. Sigma Tao, 2011. Eurartesim product information. Available at: http://www.ema.europa.eu/docs/en_GB/document_library/EPAR_-_Product_Information/human/001199/WC500118113.pdf. Accessed.
45. Whirl-Carrillo M, McDonagh EM, Hebert JM, Gong L, Sangkuhl K, Thorn CF, Altman RB, Klein TE, 2012. Pharmacogenomics knowledge for personalized medicine. Clin Pharmacol Ther 92: 414-7.
46. Merck & Co, 2010. Ivermectin product information. Available at: https://www.merck.com/product/usa/pi_circulars/s/stromectol/stromectol_pi.pdf. Accessed.
47. El-Tahtawy A, Glue P, Andrews EN, Mardekian J, Amsden GW, Knirsch CA, 2008. The effect of azithromycin on ivermectin pharmacokinetics--a population pharmacokinetic model analysis. PLoS Negl Trop Dis 2: e236.
48. Baraka OZ, Mahmoud BM, Marschke CK, Geary TG, Homeida MM, Williams JF, 1996. Ivermectin distribution in the plasma and tissues of patients infected with Onchocerca volvulus. Eur J Clin Pharmacol 50: 407-10.
49. Tarning J, Thana P, Phyo AP, Lwin KM, Hanpithakpong W, Ashley EA, Day NP, Nosten F, White NJ, 2014. Population Pharmacokinetics and Antimalarial Pharmacodynamics of Piperaquine in Patients With Plasmodium vivax Malaria in Thailand. CPT Pharmacometrics Syst Pharmacol 3: e132.
50. Ashley EA, Stepniewska K, Lindegardh N, Annerberg A, Tarning J, McGready R, Phaiphun L, Singhasivanon P, White NJ, Nosten F, 2010. Comparison of plasma, venous and capillary blood levels of piperaquine in patients with uncomplicated falciparum malaria. Eur J Clin Pharmacol 66: 705-12.
51. Bousema T, Dinglasan RR, Morlais I, Gouagna LC, van Warmerdam T, Awono-Ambene PH, Bonnet S, Diallo M, Coulibaly M, Tchuinkam T, Mulder B, Targett G, Drakeley C, Sutherland C, Robert V, Doumbo O, Toure Y, Graves PM, Roeffen W, Sauerwein R, Birkett A, Locke E, Morin M, Wu Y, Churcher TS, 2012. Mosquito feeding assays to determine the infectiousness of naturally infected Plasmodium falciparum gametocyte carriers. PLoS One 7: e42821.
52. Kannankeril P, Norris KJ, Carter S, Roden DM, 2011. Factors Affecting the Degree of QT Prolongation with Drug Challenge in a Large Cohort of Normal Volunteers: QT Prolongation with Drug Challenge in Normals. Heart Rhythm 8: 1530-1534.

53. Manning J, Vanachayangkul P, Lon C, Spring M, So M, Sea D, Se Y, Somethy S, Phann ST, Chann S, Sriwichai S, Buathong N, Kuntawunginn W, Mitprasat M, Siripokasupkul R, Teja-Isavadharm P, Soh E, Timmermans A, Lanteri C, Kaewkungwal J, Auayporn M, Tang D, Chour CM, Prom S, Haigney M, Cantilena L, Saunders D, 2014. Randomized, Double-Blind, Placebo-Controlled Clinical Trial of a Two-Day Regimen of Dihydroartemisinin-Piperaquine for Malaria Prevention Halted for Concern over Prolonged Corrected QT Interval. Antimicrob Agents Chemother 58: 6056-6067.
54. Ouédraogo AL, Bastiaens G, Tiono A, Guelbeogo M, Lanke K, Ouédraogo A, Aïssata B, Ouattara MS, Nebie I, Sauerwein RW, Sirima S, Drakeley C, Bousema T, 2013. Efficacy and safety of ivermectin to prevent malaria transmission after treatment of P. falciparum infections with artemether-lumefantrine: a double-blind randomized clinical trial. ASTMH Annual Meeting. Washington D.C.
55. Krolewiecki AJ, Ramanathan R, Fink V, McAuliffe I, Cajal SP, Won K, Juarez M, Di Paolo A, Tapia L, Acosta N, Lee R, Lammie P, Abraham D, Nutman TB, 2010. Improved diagnosis of Strongyloides stercoralis using recombinant antigen-based serologies in a community-wide study in northern Argentina. Clin Vaccine Immunol 17: 1624-30.
56. Ouédraogo AL, Guelbéogo WM, Cohuet A, Morlais I, King JG, Gon-çalves BP, Bastiaens GJH, Vaanhold M, Sattabongkot J, Wu Y, Coulibaly M, Ibrahima B, Jones S, Morin M, Drakeley C, Dinglasan RR, Bousema T, 2013. A protocol for membrane feeding assays to determine the infectiousness of P. falciparum naturally infected individuals to Anopheles gambiae. Malaria World Journal 4: supplement.
57. Stone WJ, Eldering M, van Gemert GJ, Lanke KH, Grignard L, van de Vegte-Bolmer MG, Siebelink-Stoter R, Graumans W, Roeffen WF, Drakeley CJ, Sauerwein RW, Bousema T, 2013. The relevance and applicability of oocyst prevalence as a read-out for mosquito feeding assays. Sci Rep 3: 3418.
58. Bell AS, Ranford-Cartwright LC, 2004. A real-time PCR assay for quantifying Plasmodium falciparum infections in the mosquito vector. Int J Parasitol 34: 795-802.
59. World Wide Antimalarial Resistance Network (WWARN), 2014. https://www.wwarn.org/toolkit/analyse. Accessed.
60. National Institute for Health Research - National Health Services UK (NHS), 2014. CT-toolokit. Available at: http://www.ct-toolkit.ac.uk/glossary/suspected-unexpected-serious-adverse-reactions-susar. Accessed 15 September 2014, 2014.
61. Mytton OT, Ashley EA, Peto L, Price RN, La Y, Hae R, Singhasivanon P, White NJ, Nosten F, 2007. Electrocardiographic safety evaluation of dihydroartemisinin piperaquine in the treatment of uncomplicated falciparum malaria. Am J Trop Med Hyg 77: 447-50.
62. Keating GM, 2012. Dihydroartemisinin/Piperaquine: A Review of its Use in the Treatment of Uncomplicated Plasmodium falciparum Malaria. Drugs 72: 937-61.
63. Bigira V, Kapisi J, Clark TD, Kinara S, Mwangwa F, Muhindo MK, Osterbauer B, Aweeka FT, Huang L, Achan J, Havlir DV, Rosenthal PJ, Kamya MR, Dorsey G, 2014. Protective efficacy and safety of three antimalarial regimens for the prevention of malaria in young ugandan children: a randomized controlled trial. PLoS Med 11: e1001689.
64. 2010. Uniform requirements for manuscripts submitted to biomedical journals: Writing and editing for biomedical publication. J Pharmacol Pharmacother 1: 42-58.

14. FINANCIAL ASPECTS AND CONFLICT OF INTEREST

14.1. FUNDING OF THE TRIAL

Funding to conduct the trial is provided by MESA from ISGlobal in Spain, which in turn is funded by a grant from the Bill and Melinda Gates Foundation. The Liverpool School of Tropical Medicine is providing salary support for FtK. The US Centers for Disease control and Prevention (CDC) is providing salary support for MD, based in Kenya, and infrastructural support for the trial conduct in Kenya and centralised data management.

The funder had no role in the design of this trial and will not have any during the execution, analysis, interpretation of the data, or decision to submit the results

14.2. PROVISION OF THE STUDY DRUGS

DP, Ivermectin and placebo will be sought free of charge or purchased from one of the major global manufactures (e.g. DP: Sigma Tau, Italy, or HolleyCotec, China, etc; ivermectin: Merck Sharp & Dohme Corp or Roux-Ocefa, Argentina, Valeant, Argentina, Abbot-India, etc). The study will provide copies of safety reports of SAEs and AEs to the manufacturer (expedited where required). The manufacturers will not be involved in the design of the trial.

15. BUDGET & BUDGET JUSTIFICATION

See 16.4 “Appendix IV. Budget and budget justification”, page 71.

16. APPENDICES

16.1. APPENDIX I. ROLE INVESTIGATORS

16.1.1. Protocol development: authors' contributions

Feiko ter Kuile (FtK) and Menno Smit (MS) conceived the study. MS, Eric Ochomo (EO), and FtK drafted the protocol. Duolao Wang (DW) provided statistical expertise and verified the sample size calculation. Ghaith Aljayyoussi (GA) and Steve ward (SW) conducted the Monte Carlo simulations to define the dosing regimen and further developed the pharmacokinetic sub studies. MS, PPH and FtK wrote the grant. All investigators contributed to the refinement of the study protocol and approved the final version.

16.1.2. Role Investigators

Menno Smit (MD, MPH), is a PhD candidate at LSTM, based at KEMRI/CDC in Kisumu and will act as co-principal investigator and trial coordinator. He will serve as the medical doctor on the study, overseeing clinical aspects.

Eric Ochomo (MS), is an entomologist at KEMRI/CDC with a PhD from Maseno University. He is the co-principal investigator together with Menno Smit, and together with Prof Feiko ter Kuile will share overall responsibility for the study and serve as the lead entomologist on the study. He will oversee all aspects of the membrane feeding, including mosquito rearing, mosquito survival and collection for specimen for oocysts and sporozoite analysis by PCR or ELISA.

Feiko Ter Kuile (MD, PhD), is a Professor of Tropical Epidemiology from the Liverpool School of Tropical Medicine (LSTM) based at KEMRI in Kisumu. He is the Chief Investigator and grant holder and will carry overall responsibility for the coordination of the trial and for the linkages with the sponsor, funders and with international partners involved with similar transmission reduction research.

Titus Kwambai (MD, MS), is a Kenyan Medical Officer from the Ministry of Health and based at KEMRI as researcher and a PhD candidate at LSTM. He will provide clinical expertise to the study.

Simon Kariuki (PhD), is the KEMRI/CDC Malaria Branch Chief and will take leadership for technical malaria components, laboratory oversight, liaising with research staff, and KEMRI/CDC management.

Nabie Bayoh (PhD), is a KEMRI/CDC Entomologist and will support Eric Ochomo to provide supervision and training of entomological staff, and technical guidance, and liaison with the MoH.

Meghna Desai (PhD, MPH), is a CDC Senior Epidemiologist and Technical Advisor to the Malaria Branch based at KEMRI/CDC. She will provide liaison with USA specialist advisors, MoH collaborators, and linkage with other local malaria field programs.

John Gimnig (PhD), is an entomologist at CDC in Atlanta and will provide regular scientific support and supervision to the entomological team in Kenya.

Penelope Phillips-Howard (PhD), is a Public Health Epidemiologist at LSTM, based in Kisumu, and will support protocol development and reporting and liaise with ministry and technical advisors.

Aaron Samuels (MD, MHS), is a Medical Officer and Epidemiologist at the CDC Malaria Branch in Atlanta, and will provide technical advice on ivermectin and field research activities.

Ben Abong'o (MS), is an entomologist at KEMRI/CDC, and will work on aspects of membrane feeding, including mosquito rearing, mosquito survival and monitoring, oocysts and sporozoite analysis by PCR or ELISA.

16.1.3. Role Non-engaged Collaborators

Prof Steve Ward, PhD [SW] is Professor of Parasitology at the Liverpool School of Tropical Medicine specialised in tropical pharmacology and drug discovery. He supervise Dr Ghaith Aljayyoussi and will coordinate the pharmacokinetic analysis of the samples in the laboratories in Liverpool UK.

Dr Ghaith Aljayyoussi, PhD [GA] is a post-doctoral research assistant at the Liverpool School of Tropical Medicine specialised in tropical pharmacology and pharmacokinetics. He will support the pharmacokinetic modelling and regimen design component and the pharmacokinetic analysis of the samples in the laboratories in Liverpool UK.

Prof Duolao Wang, PhD [DW] is Professor of Medical Statistics at the Liverpool School of Tropical Medicine and will be the trial statistician.

16.2. APPENDIX II. TERMS OF REFERENCE OVERSIGHT COMMITTEES

16.2.1. Trial Management Group (TMG)

16.2.1.1. Purpose

The TMG is responsible for the administrative management and day to day running of the trial.

16.2.1.2. Membership

1. Co-Principal Investigators
2. Site clinicians
3. Trial Coordinator
4. Administrators
5. Others who are involved in the day to day running of the trial
6. Chief Investigator (ad hoc)

The TMC will be chaired by one of the Principal Investigators or the Trial Coordinator.

16.2.1.3. Responsibilities:

- Study planning
- Organisation of Trial Steering Committee and Data Monitoring and Ethics Committee (DMEC) meetings
- Provide risk report to regulators, manufacture and ethics committees
- SUSAR [Serious unexpected suspected adverse events] reporting
- Responsible for trial master file
- Budget administration and contractual issues
- Advice for lead investigators
- Organisation of central data management and sample collection

16.2.2. Trial Steering Committee (TSC)

16.2.2.1. Membership TSC

Independent members

1. Chair: Dr Teun Bousema, PhD
Department of Medical Microbiology, Radboud University Medical Center, Nijmegen, The Netherlands, and London School of Hygiene and Tropical Medicine, London, UK, teun.bousema@radboudumc.nl

2. Dr. Kevin Kobilinsky
Entomology Branch, Walter Reed Army Institute of Research, Silver Spring, MD 20910, USA, kevin.c.kobylinski.ctr@us.army.mil

3. Dr Brian D Foy
Arthropod-borne and Infectious Diseases Laboratory, Department of Microbiology, Immunology & Pathology, Colorado State University, Fort Collins, Colorado, USA, brian.foy@colostate.edu

Trial members

- The Chief Investigator and Co-Principal Investigators.
- Other co-investigators and the trial statistician will attend the meetings if and when required.

16.2.2.2. Roles and Responsibilities TSC

The TSC is a trial governing body which includes a majority of its members who are independent of the trial management group. The TSC concentrates on the progress of the trial and ensures that the trial is conducted to the standards set out in the Guidelines for Good Clinical Practice with consideration given to participant safety and provision of informed consent.

- To evaluate the progress of the trial in relation to timeliness, data quality and other factors that can affect the overall objectives of the trial
- To ensure participant rights and safety are adhered to and that the protocol demands freely given informed consent
- To review relevant information from other sources
- To consider the recommendations of the Data Monitoring and Ethics Committee (DMEC) and in light of it to inform the Chief Investigator and TMG the need to changes to the trial protocol
- To ensure that the trial results are disseminated appropriately and consideration be given to the implementation of the results into policy

16.2.2.3. Operational TSC

The CI will present the full protocol to the TSC as an agenda before the start-up of data collection. The TSC members shall review the timeline set out in the protocol for participant recruitment, informed consent documents and plans for data safety monitoring.

The TSC shall see that the finalised protocol is sent to the sponsor and funders before the start of participant recruitment and data collection.

The TSC in its first meeting shall approve the nominated members of the DMEC and establish the DMEC which shall meet regularly to review and report on the data quality and the results of interim analyses.

In all their deliberations the TSC should consider any deviations from the trial protocol, participant safety and information provided to the participants and consenting procedures.

16.2.2.4. Frequency of Meetings

The TSC shall have an initial face to face start up meeting to discuss the protocol and establish the DMEC. A second meeting shall take place before the initiation of the trial to finalise the protocol and approve the commencement of the trial. Thereafter the TSC will normally meet once a year in the life span of the trial and one meeting at the closure of the trial.

The Chair and at least 2 of the three independent members together with the CI and trial co-ordinator shall constitute the quorum. If so required, in addition a member of the funder can be invited to attend the meetings.

16.2.2.5. Trial Reports and actions TSC meeting

The TSC shall provide at each meeting a summary report of their findings and recommendations which must be submitted to the funder, the Sponsor and the TMG.

If the TSC makes a recommendation that the trial should be stopped or suspended, the Sponsor will take the necessary action to ensure that new recruitment to the trial is stopped whilst the TSC report is evaluated and the Research Ethics Committee is informed.

16.2.3. Data Monitoring and Ethics Committee (DMEC)

16.2.3.1. Membership DMEC

1. Alejandro Krolewiecki, chair, topic expert and Infectious Disease Physician, Consejo Nacional de Investigaciones Científicas y Técnicas (CONICET) Buenos Aires, Argentina.
2. Prof James Oloo. local physician and University Professor, Kisumu, Kenya.
3. Dr Timothy Collier, statistician, London School of Hygiene and Tropical Medicine, UK.
4. Dr Carlos Chaccour, topic expert, Physician, Clínica Universidad de Navarra & Medical Research Fellow Barcelona Institute for Global Health (ISGlobal), Barcelona, Spain.

16.2.3.2. Role DMEC

The DMEC consist of 3-4 members (including one or more clinicians and one statistician, all with experience in clinical trials).

The DMEC shall assess the data regularly (before the annual TSC meeting) to review the data and the interim analysis. The assessment could be via email or other electronic medium annually prior to the TSC meeting. In the first year of recruitment more frequent assessment (bi-annually) is recommended for this trial with one face to face meeting at least once during the trial.

The members should be the only personnel to see the results separated by treatment group during the trial. They are independent and look at the trial from an ethical point of view of the participant safety, future patients and society in general. It is their responsibility to prevent patients being exposed to any excess risks by recommending to the Trial Steering Committee (TSC) for the trial suspension or termination early if the safety or efficacy results are sufficiently convincing. The trial statistician is usually invited to attend part of the DMEC meeting to present the most current data from the trial. This will be blinded, unless the DMEC specifically requests for an unblinded analysis.

16.2.3.3. Responsibilities DMEC

- To determine how frequently interim analysis of trial data should be undertaken.
- To consider the blinded or unblinded interim data from the trial and relevant information from other sources.
- To consider any requests for unblinding and release of interim trial data and to recommend to the TSC on the importance of this.
- To report (following each DMEC meeting) to the TSC and to recommend whether the trial should continue, the protocol be modified or the trial be stopped.

A full confidence report should be submitted in writing to the TSC at the end of each DMEC meeting

16.3. APPENDIX III. DECLARATION OF HELSINKI

WORLD MEDICAL ASSOCIATION DECLARATION OF HELSINKI

Recommendations guiding physicians in

Biomedical research involving human subjects

Adopted by the 18th World Medical Assembly, Helsinki

, Finland, June 1964,

Amended by the 29th World Medical Assembly, Tokyo, Japan, October 1975,

35th World Medical Assembly, Venice, Italy, October 1983

41st World Medical Assembly Hong Kong, September 1989

and the

48th General Assembly, Somerset West, Republic of South Africa,

October 1996

INTRODUCTION

It is the mission of the physician to safeguard the health of the people. His or her knowledge and conscience are dedicated to the fulfillment of this mission.

The Declaration of Geneva of the World Medical Association binds the physician with the words, "The health of my patient will be my first consideration", and the International Code of Medical Ethics declares that, "A physician shall act only in the patient's interest when providing medical care which might have the effect of weakening the physical and mental condition of the patient."

The purpose of biomedical research involving human subjects must be to improve diagnostic, therapeutic and prophylactic procedures and the understanding of the aetiology and pathogenesis of disease.

In current medical practice most diagnostic, therapeutic or prophylactic procedures involve hazards. This applies especially to biomedical research.

Medical progress is based on research which ultimately must rest in part on experimentation involving human subjects.

In the field of biomedical research a fundamental distinction must be recognized between medical research in which the aim is essentially diagnostic or therapeutic for a patient, and medical research, the essential object of which is purely scientific and without implying direct diagnostic or therapeutic value to the person subjected to the research.

Special caution must be exercised in the conduct of research which may affect the environment, and the welfare of animals used for research must be respected.

Because it is essential that the results of laboratory experiments be applied to human beings to further scientific knowledge and to help suffering humanity, the World Medical Association has prepared the following recommendations as a guide to every physician in biomedical research involving human subjects. They should be kept under review in the future. It must be stressed that the standards as drafted are only a guide to physicians all over the world. Physicians are not relieved from criminal, civil and ethical responsibilities under the laws of their own countries.

Basic principles

1. Biomedical research involving human subjects must conform to generally accepted scientific principles and should be based on adequately performed laboratory and animal experimentation and on a thorough knowledge of the scientific literature.
2. The design and performance of each experimental procedure involving human subjects should be clearly formulated in an experimental protocol which should be transmitted for consideration, comment and guidance to a specially appointed committee independent of the investigator and the sponsor provided that this independent committee is in conformity with the laws and regulations of the country in which the research experiment is performed.
3. Biomedical research involving human subjects should be conducted only by scientifically qualified persons and under the supervision of a clinically competent medical person. The responsibility for the human subject must always rest with a medically qualified person and never rest on the subject of the research, even though the subject has given his or her consent.
4. Biomedical research involving human subjects cannot legitimately be carried out unless the importance of the objective is in proportion to the inherent risk to the subject.
5. Every biomedical research project involving human subjects should be preceded with careful assessment of predictable risks in comparison with foreseeable benefits to the subject or to others. Concern for the interests of the subject must always prevail over the interests of science and society.
6. The right of the research subject to safeguard his or her integrity must always be respected. Every precaution should be taken to respect the privacy of the subject and to minimize the impact of the study on the subject's physical and mental integrity and on the personality of the subject.
7. Physicians should abstain from engaging in research projects involving human subjects unless they are satisfied that the hazards involved are believed to be predictable. Physicians should cease any investigation if the hazards are found to outweigh the potential benefits.
8. In publication of the results of his or her research, the physician is obliged to preserve the accuracy of the results. Reports of experimentation not in accordance with the principles laid down in this Declaration should not be accepted for publication.
9. In any research on human beings, each potential subject must be adequately informed of the aims, methods, anticipated benefits and potential hazards of the study and the discomfort it may entail. He or she should be informed that he or she is at liberty to abstain from participation in the study and that he or she is free to withdraw his or her consent to participation at any time. The physician should then obtain the subject's freely-given informed consent, preferably in writing.

10. When obtaining informed consent for the research project, the physician should be particularly cautious if the subject is in a dependent relationship to him or her or may consent under duress. In that case the informed consent should be obtained by a physician who is not engaged in the investigation and who is completely independent of this official relationship.
11. In case of legal incompetence, informed consent should be obtained from the legal guardian in accordance with national legislation. Where physical or mental incapacity makes it impossible to obtain informed consent, or when the subject is a minor, permission from the responsible relative replaces that of the subject in accordance with national legislation. Whenever the minor child is in fact able to give a consent, the minor's consent must be obtained in addition to the consent of the minor's legal guardian.
12. The research protocol should always contain a statement of the ethical considerations involved and should indicate that the principles enunciated in the present Declaration are complied with.

Medical research combined with professional care (Clinical Research)

1. In the treatment of the sick person, the physician must be free to use a new diagnostic and therapeutic measure, if in his or her judgement it offers hope of saving life, re-establishing health or alleviating suffering.
2. The potential benefits, hazards and discomfort of a new method should be weighed against the advantages of the best current diagnostic and therapeutic methods.
3. In any medical study, every patient - including those of a control group, if any - should be assured of the best proven diagnostic and therapeutic method. This does not exclude the use of inert placebo in studies where no proven diagnostic or therapeutic method exists.
4. The refusal of the patient to participate in a study must never interfere with the physician-patient relationship.
5. If the physician considers it essential not to obtain informed consent, the specific reasons for this proposal should be stated in the experimental protocol for transmission to the independent committee (I,2).
6. The physician can combine medical research with professional care, the objective being the acquisition of new medical knowledge, only to the extent that medical research is justified by its potential diagnostic or therapeutic value for the patient.

Non-therapeutic biomedical research involving human subjects (Non-clinical biomedical research)

1. In the purely scientific application of medical research carried out on a human being, it is the duty of the physician to remain the protector of the life and health of that person on whom biomedical research is being carried out.
2. The subjects should be volunteers -- either healthy persons or patients for whom the experimental design is not related to the patient's illness.
3. The investigator or the investigating team should discontinue the research if in his/her or their judgement it may, if continued, be harmful to the individual.
4. In research on man, the interest of science and society should never take precedence over considerations related to the well-being of the subject.

16.4. APPENDIX IV. BUDGET AND BUDGET JUSTIFICATION

16.4.1. Budget

***Table** 8 Budget*

Description	Cost (USD)
Laboratory supplies	9,434
Entomology supplies	43,729
Personnel	46,422
Transport	9,451
Travel	15,000
**Sub-Total**	**124,036**
KEMRI Overhead (3%)	**3,907**
KEMRI/CDC Overhead (20%)	**26,047**
Unforeseen	6,202
**Grand Total**	**160,193**

16.4.2. Budget Justification

Laboratory supplies $ 9,434

Important laboratory procedures will be critical to realization of study objectives. These include microscopy, haemoglobin and pregnancy tests. Provisions will also be made for laboratory consumables which are required for the day to day running of the laboratories.

Entomology supplies $43,729

Membrane feeding studies make up an important part of study outcomes. Supplies include larval trays, mosquito cages, dissecting forceps and disposables.

Personnel $ 46,422

Key staff will be needed to collect vital study information. These include nurses, microscopists, laboratory technicians and entomology assistants.

Transport $ 6,581

Travel by study staff will be required to the study sites for all the activities including day to day routine engagement with study participants. Transport reimbursement will be given to all study participants in order to attend clinic visits.

Travel $15,000

International travel for study investigators based abroad.

Base contribution $ 29,955

All projects within the KEMRI/CDC Research and Public Collaboration contribute to the administration of the whole program. Project ICST contribution will be 3% to KEMRI and 20% to KEMRI/CDC; a total of 23% of the total budget costs.

16.5. APPENDIX V. SPIRIT 2013 CHECKLIST CLINICAL TRIAL PROTOCOL

SPIRIT 2013 Checklist: Recommended items to address in a clinical trial protocol and related documents*

SPIRIT — Standard Protocol Items: Recommendations for Interventional Trials

Section/item	Item No	Description	Addressed on page number
**Administrative information**			
Title	1	Descriptive title identifying the study design, population, interventions, and, if applicable, trial acronym	1
Trial registration	2a	Trial identifier and registry name. If not yet registered, name of intended registry	1
	2b	All items from the World Health Organization Trial Registration Data Set	8
Protocol version	3	Date and version identifier	1
Funding	4	Sources and types of financial, material, and other support	1, 62, 71
Roles and responsibilities	5a	Names, affiliations, and roles of protocol contributors	1 and 63
	5b	Name and contact information for the trial sponsor	1
	5c	Role of study sponsor and funders, if any, in study design; collection, management, analysis, and interpretation of data; writing of the report; and the decision to submit the report for publication, including whether they will have ultimate authority over any of these activities	62
	5d	Composition, roles, and responsibilities of the coordinating centre, steering committee, endpoint adjudication committee, data management team, and other individuals or groups overseeing the trial, if applicable (see Item 21a for data monitoring committee)	65 to 67
**Introduction**			

Background and rationale	6a	Description of research question and justification for undertaking the trial, including summary of relevant studies (published and unpublished) examining benefits and harms for each intervention	15 to 21
	6b	Explanation for choice of comparators	17
Objectives	7	Specific objectives or hypotheses	22
Trial design	8	Description of trial design including type of trial (eg, parallel group, crossover, factorial, single group), allocation ratio, and framework (eg, superiority, equivalence, noninferiority, exploratory)	22
Methods: Participants, interventions, and outcomes			
Study setting	9	Description of study settings (eg, community clinic, academic hospital) and list of countries where data will be collected. Reference to where list of study sites can be obtained	26
Eligibility criteria	10	Inclusion and exclusion criteria for participants. If applicable, eligibility criteria for study centres and individuals who will perform the interventions (eg, surgeons, psychotherapists)	28
Interventions	11a	Interventions for each group with sufficient detail to allow replication, including how and when they will be administered	23 to24, 28
	11b	Criteria for discontinuing or modifying allocated interventions for a given trial participant (eg, drug dose change in response to harms, participant request, or improving/worsening disease)	29 to 31
	11c	Strategies to improve adherence to intervention protocols, and any procedures for monitoring adherence (eg, drug tablet return, laboratory tests)	31
	11d	Relevant concomitant care and interventions that are permitted or prohibited during the trial	32
Outcomes	12	Primary, secondary, and other outcomes, including the specific measurement variable (eg, systolic blood pressure), analysis metric (eg, change from baseline, final value, time to event), method of aggregation (eg, median, proportion), and time point for each outcome. Explanation of the clinical relevance of chosen efficacy and harm outcomes is strongly recommended	32 to 33
Participant timeline	13	Time schedule of enrolment, interventions (including any run-ins and washouts), assessments, and visits for participants. A schematic diagram is highly recommended (see Figure)	13, 33, 39, 15

Sample size	14	Estimated number of participants needed to achieve study objectives and how it was determined, including clinical and statistical assumptions supporting any sample size calculations	35
Recruitment	15	Strategies for achieving adequate participant enrolment to reach target sample size	36
Methods: Assignment of interventions (for controlled trials)			
Allocation:			
Sequence generation	16a	Method of generating the allocation sequence (eg, computer-generated random numbers), and list of any factors for stratification. To reduce predictability of a random sequence, details of any planned restriction (eg, blocking) should be provided in a separate document that is unavailable to those who enrol participants or assign interventions	37
Allocation concealment mechanism	16b	Mechanism of implementing the allocation sequence (eg, central telephone; sequentially numbered, opaque, sealed envelopes), describing any steps to conceal the sequence until interventions are assigned	37
Implementation	16c	Who will generate the allocation sequence, who will enrol participants, and who will assign participants to interventions	37
Blinding (masking)	17a	Who will be blinded after assignment to interventions (eg, trial participants, care providers, outcome assessors, data analysts), and how	37
	17b	If blinded, circumstances under which unblinding is permissible, and procedure for revealing a participant's allocated intervention during the trial	29, 37, , 42, 45, 46, 67
Methods: Data collection, management, and analysis			
Data collection methods	18a	Plans for assessment and collection of outcome, baseline, and other trial data, including any related processes to promote data quality (eg, duplicate measurements, training of assessors) and a description of study instruments (eg, questionnaires, laboratory tests) along with their reliability and validity, if known. Reference to where data collection forms can be found, if not in the protocol	40
	18b	Plans to promote participant retention and complete follow-up, including list of any outcome data to be collected for participants who discontinue or deviate from intervention protocols	29 to 31

Data management	19	Plans for data entry, coding, security, and storage, including any related processes to promote data quality (eg, double data entry; range checks for data values). Reference to where details of data management procedures can be found, if not in the protocol	40
Statistical methods	20a	Statistical methods for analysing primary and secondary outcomes. Reference to where other details of the statistical analysis plan can be found, if not in the protocol	42
	20b	Methods for any additional analyses (eg, subgroup and adjusted analyses)	42
	20c	Definition of analysis population relating to protocol non-adherence (eg, as randomised analysis), and any statistical methods to handle missing data (eg, multiple imputation)	42
Methods: Monitoring			
Data monitoring	21a	Composition of data monitoring committee (DMC); summary of its role and reporting structure; statement of whether it is independent from the sponsor and competing interests; and reference to where further details about its charter can be found, if not in the protocol. Alternatively, an explanation of why a DMC is not needed	45 and 67
	21b	Description of any interim analyses and stopping guidelines, including who will have access to these interim results and make the final decision to terminate the trial	46
Harms	22	Plans for collecting, assessing, reporting, and managing solicited and spontaneously reported adverse events and other unintended effects of trial interventions or trial conduct	46
Auditing	23	Frequency and procedures for auditing trial conduct, if any, and whether the process will be independent from investigators and the sponsor	50
Ethics and dissemination			
Research ethics approval	24	Plans for seeking research ethics committee/institutional review board (REC/IRB) approval	51
Protocol amendments	25	Plans for communicating important protocol modifications (eg, changes to eligibility criteria, outcomes, analyses) to relevant parties (eg, investigators, REC/IRBs, trial participants, trial registries, journals, regulators)	52

Consent or assent	26a	Who will obtain informed consent or assent from potential trial participants or authorised surrogates, and how (see Item 32)	52
	26b	Additional consent provisions for collection and use of participant data and biological specimens in ancillary studies, if applicable	52
Confidentiality	27	How personal information about potential and enrolled participants will be collected, shared, and maintained in order to protect confidentiality before, during, and after the trial	53
Declaration of interests	28	Financial and other competing interests for principal investigators for the overall trial and each study site	53
Access to data	29	Statement of who will have access to the final trial dataset, and disclosure of contractual agreements that limit such access for investigators	53
Ancillary and post-trial care	30	Provisions, if any, for ancillary and post-trial care, and for compensation to those who suffer harm from trial participation	55
Dissemination policy	31a	Plans for investigators and sponsor to communicate trial results to participants, healthcare professionals, the public, and other relevant groups (eg, via publication, reporting in results databases, or other data sharing arrangements), including any publication restrictions	56
	31b	Authorship eligibility guidelines and any intended use of professional writers	57
	31c	Plans, if any, for granting public access to the full protocol, participant-level dataset, and statistical code	57
Appendices			
Informed consent materials	32	Model consent form and other related documentation given to participants and authorised surrogates	84
Biological specimens	33	Plans for collection, laboratory evaluation, and storage of biological specimens for genetic or molecular analysis in the current trial and for future use in ancillary studies, if applicable	25, 87

16.6. APPENDIX VI. PRODUCT CHARACTERISTICS

Ivermectin product insert (Stromectol)

This product info is provided as an example. The brand name and manufacturer of ivermectin have not yet been finalised at the time of writing.

Merck Sharp & Dohme Corp., a subsidiary of
MERCK & CO., INC.
Whitehouse Station, NJ 08889, USA

9032319
87447/080610
8495

TABLETS
STROMECTOL®
(IVERMECTIN)

DESCRIPTION

STROMECTOL* (Ivermectin) is a semisynthetic, anthelmintic agent for oral administration. Ivermectin is derived from the avermectins, a class of highly active broad-spectrum, anti-parasitic agents isolated from the fermentation products of *Streptomyces avermitilis*. Ivermectin is a mixture containing at least 90% 5-*O*-demethyl-22,23-dihydroavermectin A_{1a} and less than 10% 5-*O*-demethyl-25-de(1-methylpropyl)-22,23-dihydro-25-(1-methylethyl)avermectin A_{1a}, generally referred to as 22,23-dihydroavermectin B_{1a} and B_{1b}, or H_2B_{1a} and H_2B_{1b}, respectively. The respective empirical formulas are $C_{48}H_{74}O_{14}$ and $C_{47}H_{72}O_{14}$, with molecular weights of 875.10 and 861.07, respectively. The structural formulas are:

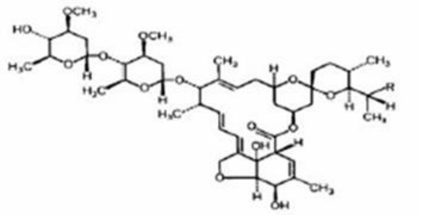

Component B_{1a}, R = C_2H_5 Component B_{1b}, R = CH_3

Ivermectin is a white to yellowish-white, nonhygroscopic, crystalline powder with a melting point of about 155°C. It is insoluble in water but is freely soluble in methanol and soluble in 95% ethanol.

STROMECTOL is available in 3-mg tablets containing the following inactive ingredients: microcrystalline cellulose, pregelatinized starch, magnesium stearate, butylated hydroxyanisole, and citric acid powder (anhydrous).

CLINICAL PHARMACOLOGY

Pharmacokinetics

Following oral administration of ivermectin, plasma concentrations are approximately proportional to the dose. In two studies, after single 12-mg doses of STROMECTOL in fasting healthy volunteers (representing a mean dose of 165 mcg/kg), the mean peak plasma concentrations of the major component (H_2B_{1a}) were 46.6 (±21.9) (range: 16.4-101.1) and 30.6 (±15.6) (range: 13.9-68.4) ng/mL, respectively, at approximately 4 hours after dosing. Ivermectin is metabolized in the liver, and ivermectin and/or its metabolites are excreted almost exclusively in the feces over an estimated 12 days, with less than 1% of the administered dose excreted in the urine. The plasma half-life of ivermectin in man is approximately 18 hours following oral administration.

The safety and pharmacokinetic properties of ivermectin were further assessed in a multiple-dose clinical pharmacokinetic study involving healthy volunteers. Subjects received oral doses of 30 to 120 mg (333 to 2000 mcg/kg) ivermectin in a fasted state or 30 mg (333 to 600 mcg/kg) ivermectin following a standard high-fat (48.6 g of fat) meal. Administration of 30 mg ivermectin following a high-fat meal resulted in an approximate 2.5-fold increase in bioavailability relative to administration of 30 mg ivermectin in the fasted state.

---

* Registered trademark of Merck Sharp & Dohme Corp., a subsidiary of **Merck & Co., Inc.**
Copyright © 1996, 2007 Merck Sharp & Dohme Corp., a subsidiary of **Merck & Co., Inc.**
All rights reserved

1

STROMECTOL® (Ivermectin) 9032319
87447/080610
8495

In vitro studies using human liver microsomes and recombinant CYP450 enzymes have shown that ivermectin is primarily metabolized by CYP3A4. Depending on the *in vitro* method used, CYP2D6 and CYP2E1 were also shown to be involved in the metabolism of ivermectin but to a significantly lower extent compared to CYP3A4. The findings of *in vitro* studies using human liver microsomes suggest that clinically relevant concentrations of ivermectin do not significantly inhibit the metabolizing activities of CYP3A4, CYP2D6, CYP2C9, CYP1A2, and CYP2E1.

Microbiology

Ivermectin is a member of the avermectin class of broad-spectrum antiparasitic agents which have a unique mode of action. Compounds of the class bind selectively and with high affinity to glutamate-gated chloride ion channels which occur in invertebrate nerve and muscle cells. This leads to an increase in the permeability of the cell membrane to chloride ions with hyperpolarization of the nerve or muscle cell, resulting in paralysis and death of the parasite. Compounds of this class may also interact with other ligand-gated chloride channels, such as those gated by the neurotransmitter gamma-aminobutyric acid (GABA).

The selective activity of compounds of this class is attributable to the facts that some mammals do not have glutamate-gated chloride channels and that the avermectins have a low affinity for mammalian ligand-gated chloride channels. In addition, ivermectin does not readily cross the blood-brain barrier in humans.

Ivermectin is active against various life-cycle stages of many but not all nematodes. It is active against the tissue microfilariae of *Onchocerca volvulus* but not against the adult form. Its activity against *Strongyloides stercoralis* is limited to the intestinal stages.

Clinical Studies

Strongyloidiasis

Two controlled clinical studies using albendazole as the comparative agent were carried out in international sites where albendazole is approved for the treatment of strongyloidiasis of the gastrointestinal tract, and three controlled studies were carried out in the U.S. and internationally using thiabendazole as the comparative agent. Efficacy, as measured by cure rate, was defined as the absence of larvae in at least two follow-up stool examinations 3 to 4 weeks post-therapy. Based on this criterion, efficacy was significantly greater for STROMECTOL (a single dose of 170 to 200 mcg/kg) than for albendazole (200 mg b.i.d. for 3 days). STROMECTOL administered as a single dose of 200 mcg/kg for 1 day was as efficacious as thiabendazole administered at 25 mg/kg b.i.d. for 3 days.

Summary of Cure Rates for Ivermectin Versus Comparative Agents in the Treatment of Strongyloidiasis

	Cure Rate* (%)	
	Ivermectin**	Comparative Agent
Albendazole*** Comparative<br>International Study<br>WHO Study	 24/26 (92)<br>126/152 (83)	 12/22 (55)<br>67/149 (45)
Thiabendazole† Comparative<br>International Study<br>US Studies	 9/14 (64)<br>14/14 (100)	 13/15 (87)<br>16/17 (94)

* Number and % of evaluable patients
** 170-200 mcg/kg
*** 200 mg b.i.d. for 3 days
† 25 mg/kg b.i.d. for 3 days

In one study conducted in France, a non-endemic area where there was no possibility of reinfection, several patients were observed to have recrudescence of *Strongyloides* larvae in their stool as long as 106 days following ivermectin therapy. Therefore, at least three stool examinations should be conducted over the three months following treatment to ensure eradication. If recrudescence of larvae is observed, retreatment with ivermectin is indicated. Concentration techniques (such as using a Baermann apparatus) should be employed when performing these stool examinations, as the number of *Strongyloides* larvae per gram of feces may be very low.

2

Onchocerciasis

The evaluation of STROMECTOL in the treatment of onchocerciasis is based on the results of clinical studies involving 1278 patients. In a double-blind, placebo-controlled study involving adult patients with moderate to severe onchocercal infection, patients who received a single dose of 150 mcg/kg STROMECTOL experienced an 83.2% and 99.5% decrease in skin microfilariae count (geometric mean) 3 days and 3 months after the dose, respectively. A marked reduction of >90% was maintained for up to 12 months after the single dose. As with other microfilaricidal drugs, there was an increase in the microfilariae count in the anterior chamber of the eye at day 3 after treatment in some patients. However, at 3 and 6 months after the dose, a significantly greater percentage of patients treated with STROMECTOL had decreases in microfilariae count in the anterior chamber than patients treated with placebo.

In a separate open study involving pediatric patients ages 6 to 13 (n=103; weight range: 17-41 kg), similar decreases in skin microfilariae counts were observed for up to 12 months after dosing.

INDICATIONS AND USAGE

STROMECTOL is indicated for the treatment of the following infections:

Strongyloidiasis of the intestinal tract. STROMECTOL is indicated for the treatment of intestinal (i.e., nondisseminated) strongyloidiasis due to the nematode parasite *Strongyloides stercoralis*.

This indication is based on clinical studies of both comparative and open-label designs, in which 64-100% of infected patients were cured following a single 200-mcg/kg dose of ivermectin. (See CLINICAL PHARMACOLOGY, *Clinical Studies*.)

Onchocerciasis. STROMECTOL is indicated for the treatment of onchocerciasis due to the nematode parasite *Onchocerca volvulus*.

This indication is based on randomized, double-blind, placebo-controlled and comparative studies conducted in 1427 patients in onchocerciasis-endemic areas of West Africa. The comparative studies used diethylcarbamazine citrate (DEC-C).

NOTE: STROMECTOL has no activity against adult *Onchocerca volvulus* parasites. The adult parasites reside in subcutaneous nodules which are infrequently palpable. Surgical excision of these nodules (nodulectomy) may be considered in the management of patients with onchocerciasis, since this procedure will eliminate the microfilariae-producing adult parasites.

CONTRAINDICATIONS

STROMECTOL is contraindicated in patients who are hypersensitive to any component of this product.

WARNINGS

Historical data have shown that microfilaricidal drugs, such as diethylcarbamazine citrate (DEC-C), might cause cutaneous and/or systemic reactions of varying severity (the Mazzotti reaction) and ophthalmological reactions in patients with onchocerciasis. These reactions are probably due to allergic and inflammatory responses to the death of microfilariae. Patients treated with STROMECTOL for onchocerciasis may experience these reactions in addition to clinical adverse reactions possibly, probably, or definitely related to the drug itself. (See ADVERSE REACTIONS, *Onchocerciasis*.)

The treatment of severe Mazzotti reactions has not been subjected to controlled clinical trials. Oral hydration, recumbency, intravenous normal saline, and/or parenteral corticosteroids have been used to treat postural hypotension. Antihistamines and/or aspirin have been used for most mild to moderate cases.

PRECAUTIONS

General

After treatment with microfilaricidal drugs, patients with hyperreactive onchodermatitis (sowda) may be more likely than others to experience severe adverse reactions, especially edema and aggravation of onchodermatitis.

Rarely, patients with onchocerciasis who are also heavily infected with *Loa loa* may develop a serious or even fatal encephalopathy either spontaneously or following treatment with an effective microfilaricide. In these patients, the following adverse experiences have also been reported: pain (including neck and back pain), red eye, conjunctival hemorrhage, dyspnea, urinary and/or fecal incontinence, difficulty in standing/walking, mental status changes, confusion, lethargy, stupor, seizures, or coma. This syndrome has been seen very rarely following the use of ivermectin. In individuals who warrant treatment with ivermectin for any reason and have had significant exposure to *Loa loa*-endemic areas of West or Central Africa, pretreatment assessment for loiasis and careful post-treatment follow-up should be implemented.

Information for Patients

STROMECTOL should be taken on an empty stomach with water. (See CLINICAL PHARMACOLOGY, *Pharmacokinetics*.)

Strongyloidiasis: The patient should be reminded of the need for repeated stool examinations to document clearance of infection with *Strongyloides stercoralis*.

Onchocerciasis: The patient should be reminded that treatment with STROMECTOL does not kill the adult *Onchocerca* parasites, and therefore repeated follow-up and retreatment is usually required.

Drug Interactions

Post-marketing reports of increased INR (International Normalized Ratio) have been rarely reported when ivermectin was co-administered with warfarin.

Carcinogenesis, Mutagenesis, Impairment of Fertility

Long-term studies in animals have not been performed to evaluate the carcinogenic potential of ivermectin.

Ivermectin was not genotoxic *in vitro* in the Ames microbial mutagenicity assay of *Salmonella typhimurium* strains TA1535, TA1537, TA98, and TA100 with and without rat liver enzyme activation, the Mouse Lymphoma Cell Line L5178Y (cytotoxicity and mutagenicity) assays, or the unscheduled DNA synthesis assay in human fibroblasts.

Ivermectin had no adverse effects on the fertility in rats in studies at repeated doses of up to 3 times the maximum recommended human dose of 200 mcg/kg (on a mg/m^2/day basis).

Pregnancy, Teratogenic Effects

Pregnancy Category C

Ivermectin has been shown to be teratogenic in mice, rats, and rabbits when given in repeated doses of 0.2, 8.1, and 4.5 times the maximum recommended human dose, respectively (on a mg/m^2/day basis). Teratogenicity was characterized in the three species tested by cleft palate; clubbed forepaws were additionally observed in rabbits. These developmental effects were found only at or near doses that were maternotoxic to the pregnant female. Therefore, ivermectin does not appear to be selectively fetotoxic to the developing fetus. There are, however, no adequate and well-controlled studies in pregnant women. Ivermectin should not be used during pregnancy since safety in pregnancy has not been established.

Nursing Mothers

STROMECTOL is excreted in human milk in low concentrations. Treatment of mothers who intend to breast-feed should only be undertaken when the risk of delayed treatment to the mother outweighs the possible risk to the newborn.

Pediatric Use

Safety and effectiveness in pediatric patients weighing less than 15 kg have not been established.

Geriatric Use

Clinical studies of STROMECTOL did not include sufficient numbers of subjects aged 65 and over to determine whether they respond differently from younger subjects. Other reported clinical experience has not identified differences in responses between the elderly and younger patients. In general, treatment of an elderly patient should be cautious, reflecting the greater frequency of decreased hepatic, renal, or cardiac function, and of concomitant disease or other drug therapy.

Strongyloidiasis in Immunocompromised Hosts

In immunocompromised (including HIV-infected) patients being treated for intestinal strongyloidiasis, repeated courses of therapy may be required. Adequate and well-controlled clinical studies have not been conducted in such patients to determine the optimal dosing regimen. Several treatments, i.e., at 2-week intervals, may be required, and cure may not be achievable. Control of extra-intestinal strongyloidiasis in these patients is difficult, and suppressive therapy, i.e., once per month, may be helpful.

STROMECTOL® (Ivermectin) 9032319
87447/080610
8495

ADVERSE REACTIONS

Strongyloidiasis

In four clinical studies involving a total of 109 patients given either one or two doses of 170 to 200 mcg/kg of STROMECTOL, the following adverse reactions were reported as possibly, probably, or definitely related to STROMECTOL:

Body as a Whole: asthenia/fatigue (0.9%), abdominal pain (0.9%)

Gastrointestinal: anorexia (0.9%), constipation (0.9%), diarrhea (1.8%), nausea (1.8%), vomiting (0.9%)

Nervous System/Psychiatric: dizziness (2.8%), somnolence (0.9%), vertigo (0.9%), tremor (0.9%)

Skin: pruritus (2.8%), rash (0.9%), and urticaria (0.9%).

In comparative trials, patients treated with STROMECTOL experienced more abdominal distention and chest discomfort than patients treated with albendazole. However, STROMECTOL was better tolerated than thiabendazole in comparative studies involving 37 patients treated with thiabendazole.

The Mazzotti-type and ophthalmologic reactions associated with the treatment of onchocerciasis or the disease itself would not be expected to occur in strongyloidiasis patients treated with STROMECTOL. (See ADVERSE REACTIONS, *Onchocerciasis*.)

Laboratory Test Findings

In clinical trials involving 109 patients given either one or two doses of 170 to 200 mcg/kg STROMECTOL, the following laboratory abnormalities were seen regardless of drug relationship: elevation in ALT and/or AST (2%), decrease in leukocyte count (3%). Leukopenia and anemia were seen in one patient.

Onchocerciasis

In clinical trials involving 963 adult patients treated with 100 to 200 mcg/kg STROMECTOL, worsening of the following Mazzotti reactions during the first 4 days post-treatment were reported: arthralgia/synovitis (9.3%), axillary lymph node enlargement and tenderness (11.0% and 4.4%, respectively), cervical lymph node enlargement and tenderness (5.3% and 1.2%, respectively), inguinal lymph node enlargement and tenderness (12.6% and 13.9%, respectively), other lymph node enlargement and tenderness (3.0% and 1.9%, respectively), pruritus (27.5%), skin involvement including edema, papular and pustular or frank urticarial rash (22.7%), and fever (22.6%). (See WARNINGS.)

In clinical trials, ophthalmological conditions were examined in 963 adult patients before treatment, at day 3, and months 3 and 6 after treatment with 100 to 200 mcg/kg STROMECTOL. Changes observed were primarily deterioration from baseline 3 days post-treatment. Most changes either returned to baseline condition or improved over baseline severity at the month 3 and 6 visits. The percentages of patients with worsening of the following conditions at day 3, month 3 and 6, respectively, were: limbitis: 5.5%, 4.8%, and 3.5% and punctate opacity: 1.8%, 1.8%, and 1.4%. The corresponding percentages for patients treated with placebo were: limbitis: 6.2%, 9.9%, and 9.4% and punctate opacity: 2.0%, 6.4%, and 7.2%. (See WARNINGS.)

In clinical trials involving 963 adult patients who received 100 to 200 mcg/kg STROMECTOL, the following clinical adverse reactions were reported as possibly, probably, or definitely related to the drug in ≥1% of the patients: facial edema (1.2%), peripheral edema (3.2%), orthostatic hypotension (1.1%), and tachycardia (3.5%). Drug-related headache and myalgia occurred in <1% of patients (0.2% and 0.4%, respectively). However, these were the most common adverse experiences reported overall during these trials regardless of causality (22.3% and 19.7%, respectively).

A similar safety profile was observed in an open study in pediatric patients ages 6 to 13.

The following ophthalmological side effects do occur due to the disease itself but have also been reported after treatment with STROMECTOL: abnormal sensation in the eyes, eyelid edema, anterior uveitis, conjunctivitis, limbitis, keratitis, and chorioretinitis or choroiditis. These have rarely been severe or associated with loss of vision and have generally resolved without corticosteroid treatment.

Laboratory Test Findings

In controlled clinical trials, the following laboratory adverse experiences were reported as possibly, probably, or definitely related to the drug in ≥1% of the patients: eosinophilia (3%) and hemoglobin increase (1%).

Post-Marketing Experience

The following adverse reactions have been reported since the drug was registered overseas:

Onchocerciasis
Conjunctival hemorrhage
All Indications
Hypotension (mainly orthostatic hypotension), worsening of bronchial asthma, toxic epidermal necrolysis, Stevens-Johnson syndrome, seizures, hepatitis, elevation of liver enzymes, and elevation of bilirubin.

OVERDOSAGE

Significant lethality was observed in mice and rats after single oral doses of 25 to 50 mg/kg and 40 to 50 mg/kg, respectively. No significant lethality was observed in dogs after single oral doses of up to 10 mg/kg. At these doses, the treatment-related signs that were observed in these animals include ataxia, bradypnea, tremors, ptosis, decreased activity, emesis, and mydriasis.

In accidental intoxication with, or significant exposure to, unknown quantities of veterinary formulations of ivermectin in humans, either by ingestion, inhalation, injection, or exposure to body surfaces, the following adverse effects have been reported most frequently: rash, edema, headache, dizziness, asthenia, nausea, vomiting, and diarrhea. Other adverse effects that have been reported include: seizure, ataxia, dyspnea, abdominal pain, paresthesia, urticaria, and contact dermatitis.

In case of accidental poisoning, supportive therapy, if indicated, should include parenteral fluids and electrolytes, respiratory support (oxygen and mechanical ventilation if necessary) and pressor agents if clinically significant hypotension is present. Induction of emesis and/or gastric lavage as soon as possible, followed by purgatives and other routine anti-poison measures, may be indicated if needed to prevent absorption of ingested material.

DOSAGE AND ADMINISTRATION

Strongyloidiasis
The recommended dosage of STROMECTOL for the treatment of strongyloidiasis is a single oral dose designed to provide approximately 200 mcg of ivermectin per kg of body weight. See Table 1 for dosage guidelines. Patients should take tablets on an empty stomach with water. (See CLINICAL PHARMACOLOGY, *Pharmacokinetics*.) In general, additional doses are not necessary. However, follow-up stool examinations should be performed to verify eradication of infection. (See CLINICAL PHARMACOLOGY, *Clinical Studies*.)

Table 1
Dosage Guidelines for STROMECTOL for Strongyloidiasis

Body Weight (kg)	Single Oral Dose<br>Number of 3-mg Tablets
15-24	1 tablet
25-35	2 tablets
36-50	3 tablets
51-65	4 tablets
66-79	5 tablets
≥80	200 mcg/kg

Onchocerciasis
The recommended dosage of STROMECTOL for the treatment of onchocerciasis is a single oral dose designed to provide approximately 150 mcg of ivermectin per kg of body weight. See Table 2 for dosage guidelines. Patients should take tablets on an empty stomach with water. (See CLINICAL PHARMACOLOGY, *Pharmacokinetics*.) In mass distribution campaigns in international treatment programs, the most commonly used dose interval is 12 months. For the treatment of individual patients, retreatment may be considered at intervals as short as 3 months.

Table 2
Dosage Guidelines for STROMECTOL for Onchocerciasis

Body Weight (kg)	Single Oral Dose Number of 3-mg Tablets
15-25	1 tablet
26-44	2 tablets
45-64	3 tablets
65-84	4 tablets
≥85	150 mcg/kg

HOW SUPPLIED

No. 8495 — Tablets STROMECTOL 3 mg are white, round, flat, bevel-edged tablets coded MSD on one side and 32 on the other side. They are supplied as follows:

NDC 0006-0032-20 unit dose packages of 20.

Storage

Store at temperatures below 30°C (86°F).

Dist. by: Merck Sharp & Dohme Corp., a subsidiary of
MERCK & CO., INC., Whitehouse Station, NJ 08889, USA

Manufactured by:
Merck Sharp & Dohme BV
Waarderweg 39
2031 BN Haarlem
Netherlands

Issued May 2010
Printed in the Netherlands

16.7. APPENDIX VII. PARTICIPANT INFORMATION SHEETS AND INFORMED CONSENT FORMS

16.7.1. Informed Consent Participant Information Sheet: Main trial (English)

Flesch-Kincaid
Reading Level: 6.7

IVERMECTIN DOSE FINDING STUDY (IVERMAL)

Participant Information Sheet

Title: Efficacy and safety of high-dose ivermectin for reducing malaria transmission: A dose finding study

Investigators

Dr Menno Smit, MD, MPH (KEMRI, LSTM)
Dr Eric Ochomo, PhD (KEMRI/CDC)
Dr Titus Kwambai, MD, MSc (MOH, KEMRI/CDC, LSTM) [TK]
Dr Simon Kariuki, PhD (KEMRI/CDC) [SK]
Dr Nabie Bayoh, PhD (KEMRI/CDC) [NB]
Dr Meghna Desai, PhD, MPH (KEMRI/CDC, CDC-Kenya) [MD]
Dr John Gimnig, PhD (CDC) [JG]
Dr Penelope Phillips-Howard, PhD (KEMRI, LSTM) [PPH]
Dr Aaron Samuels, MD, MHS (CDC) [AS]
Mr Ben Abong'o, MS (KEMRI/CDC)
Institutions

KEMRI- Kenya Medical Research Institute and CDC, Kisumu, Kenya
CDC- Centers for Disease Control and Prevention, Atlanta, Georgia, USA
MOH- Ministry of Health, Kenya
LSTM- Liverpool School of Tropical Medicine

Purpose of research study

The Kenya Medical Research Institute (KEMRI), the Centers for Disease Control and Prevention (CDC), and the Liverpool School of Tropical Medicine (LSTM) are doing a research study. People in Kenya get dihydroartemisinin-piperaquine (DP) to treat malaria. DP kills the parasite that makes you feel sick. It does not kill the mosquito. Ivermectin kills the mosquito that spreads the disease. Ivermectin does not kill the parasite that makes you feel sick. We want to test if giving ivermectin may stop malaria from spreading by the mosquito to other people. If we give you both DP and ivermectin, we know that we can decrease the spread of malaria for a short time. We want to know if a larger amount of ivermectin will stop the spread of malaria for a longer time.

What we will do
You have malaria. We will ask you if you would like to be in this study. If you say yes, we will check to see if you can be in the study. We will ask you some questions, examine you and take some blood. We will test you for low blood. If you are a woman and not known to be pregnant, we will ask to do a pregnancy test from your urine or blood. If you are pregnant or your blood is too low, you may not participate in the study and we will refer you for care. Also, if you are breastfeeding, you may not participate.

We will either give you DP and a placebo or DP and ivermectin. We will watch you take the first dose. We will ask you to return to the hospital on 10 dates we will give you. On each of these visits, a nurse will ask you questions and take some blood from your arm. On 4 visits the nurse will also do a finger prick.

We will not take more than 5.7 ml (1 teaspoon) of blood on any day. Most days we will take less than ½ teaspoon of blood. That is a very small portion of your blood. Normally your body replaces it without you ever noticing.

We will also do a heart film to monitor your heart rate today and again in 2 days and one month from now. This is called an ECG. Each ECG will take about 15 minutes.

Potential benefits
You will be treated for malaria free of charge. Ivermectin will also treat worms, scabies, bedbugs and lice. We will watch you closely for any health problems during your follow-up. We will give you treatment for conditions related to the study free of charge. We will give you a telephone number to call if you are concerned at any time.

Potential risks
There may be small bruises where we draw blood from you. The drugs DP and ivermectin are well tolerated and safe. Ivermectin can give problems to people with *Loa loa*. However, *Loa loa* is not found in Kenya. If you have travelled to a country with *Loa loa,* you may not participate in the study.

Privacy and confidentiality
Information about yourself will be kept confidential to the maximum extent allowable by law. The data we collect will be stored securely in locked cabinets and on password-protected computers at the KEMRI/CDC offices in Kisumu. Only members of the study staff will review the records with your name on it. We will use the information you give to us only for research. The information collected will be shared with other people, but your name will not appear on any reports. At the end of the study, we will remove all names from the data so that no one can identify your information or your blood sample.

Your rights to participate, say no, or withdraw
You are free to choose to be part of this study. You have the right to refuse. If you do not want to go on with this study, you can stop at any time. If you choose not to participate, it will not affect the care that you will get for malaria now. You will still be treated for malaria.

Costs and compensation for being in the study
You will not be asked to pay anything to participate in this study. Transport reimbursement for all scheduled or sick visits to our study hospital will be provided to you as per KEMRI/CDC guidelines currently at a maximum of Ksh 300-500 every time you attend a visit.

Consent for long-term storage of blood samples for future studies
We are asking people who join this study if they will let the researchers' use their blood sample for future studies. These future studies may help find new ways to prevent malaria. If you say yes, we will store your blood with a unique code and not with your name in a secure area at the KEMRI/CDC laboratories. We may share your malaria test results with researchers at other organizations but we will not give them your name, address, or any information that could identify you. After the study period has ended, we will remove any means to link the sample to you, and we will not be able to find your sample. If you do not wish to have your blood stored, you may still participate in our study. You will still be examined for malaria and low blood. If you have malaria or low blood, we will still treat you.

Contact information for questions or concerns
If you have any questions about this study, you can contact Dr Eric Ochomo at KEMRI/CDC, P.O. Box 1578-40100, Kisumu, or at 057 20 22902.You can also contact any of our study staff at the hospital. If you have any questions about your rights as a study participant, or if you want to talk about the study with someone who is not directly involved with this study, please contact The Secretary, KEMRI Scientific and Ethics Review Unit (SERU), PO Box 54840-00200, Nairobi; Telephone numbers: 020 2722541, 0722205901 or 0733400003. Email address: seru@kemri.org. You may also contact the Secretary or Chairman of the same committee if you feel you have been harmed by this study. If you do not have access to a telephone, or you do not know how to read and write, this will not stop you from participating in this study. You may ask for contact information from the nearest health facility if you wish to talk with one of the listed individuals to raise any concerns.

If you are sick, please call Dr Eric Ochomo at KEMRI/CDC at 057 20 22902 and please go to the nearest health facility.
Thank you very much for your time. If you agree to take part in this study, we will ask you to sign this form.

16.7.2. Consent Statement: Main trial (English)

IVERMECTIN DOSE FINDING STUDY
(IVERMAL)

Consent Statement

Title of Study: Efficacy and safety of high-dose ivermectin for reducing malaria transmission: A dose finding study

Your signature below means that you voluntarily agree to participate in this research:

The above has been explained to me and I agree to take part in the study. I understand that I am free to choose to be in this study and that saying "NO" will have no effect on me. I agree for my blood to be tested for malaria and low blood. I also understand and agree that some of my blood may be sent to laboratories at CDC Atlanta or LSTM Liverpool or other collaborating laboratories for tests that cannot be done in Kenya. I understand that relevant parts of my health records and facts collected during the study may be looked at by staff from KEMRI/CDC. I give permission for these persons to have access to my records. I also give permission to share the facts collected through this study, without my name and address, with other studies.	If you agree, circle "YES,"
	YES

	Name	Signature or thumbprint*	Today's date
Adult providing consent for self		Signature*	
		Thumbprint*	
Witness*			
Study staff consenting participant			

* A witness is only needed if the participant cannot read. The witness must be a person independent from the study. The participant can provide a thumbprint below and verbally state his/her consent in the presence of a witness who will then sign.

IVERMECTIN DOSE FINDING STUDY (IVERMAL)Consent for long term storage of blood samples and future studies

Title of Study: Efficacy and safety of high-dose ivermectin for reducing malaria transmission: A dose finding study

Your signature below means that you voluntarily agree for your samples to be stored long term for future research studies:

<table>
<tr><td rowspan="2">The storage of my blood was explained to me and I agree for KEMRI/CDC to store my blood sample for at least 15 years for future studies. I also understand and agree that my blood samples may be sent to laboratories at CDC Atlanta or LSTM Liverpool or other collaborating laboratories for tests that cannot be done in Kenya. I understand that I can change my mind to not have my blood sample sent, stored and used for future research. To do this, I may tell Dr. Meghna Desai of my request and they will tell the other study staff at KEMRI/CDC.</td><td colspan="2">If you agree circle “YES”, if you do not agree circle ‘NO’</td></tr>
<tr><td>YES</td><td>NO</td></tr>
</table>

<table>
<tr><td></td><td>Name</td><td>Signature or thumbprint*</td><td>Today’s date</td></tr>
<tr><td rowspan="2">Adult providing consent for self</td><td></td><td>Signature*</td><td></td></tr>
<tr><td></td><td>Thumbprint*</td><td></td></tr>
<tr><td>Witness*</td><td></td><td></td><td></td></tr>
<tr><td>Study staff consenting participant</td><td></td><td></td><td></td></tr>
</table>

* A witness is only needed if the participant cannot read. The witness must be a person independent from the study. The participant can sign or verbally state his/her consent in the presence of a witness who will then sign.

16.7.3. Informed Consent Participant Information Sheet: Rich PK study (English)

IVERMECTIN DOSE FINDING STUDY
(IVERMAL)

Participant Information Sheet:
Pharmacokinetic (PK) study

Title: Efficacy and safety of high-dose ivermectin for reducing malaria transmission: A dose finding study

Investigators

Dr Menno Smit, MD, MPH (KEMRI, LSTM)
Dr Eric Ochomo, PhD (KEMRI/CDC)
Dr Titus Kwambai, MD, MSc (MOH, KEMRI/CDC, LSTM) [TK]
Dr Simon Kariuki, PhD (KEMRI/CDC) [SK]
Dr Nabie Bayoh, PhD (KEMRI/CDC) [NB]
Dr Meghna Desai, PhD, MPH (KEMRI/CDC, CDC-Kenya) [MD]
Dr John Gimnig, PhD (CDC) [JG]
Dr Penelope Phillips-Howard, PhD (KEMRI, LSTM) [PPH]
Dr Aaron Samuels, MD, MHS (CDC) [AS]
Mr Ben Abong'o, MS (KEMRI/CDC)
Institutions

KEMRI- Kenya Medical Research Institute and CDC, Kisumu, Kenya
CDC- Centers for Disease Control and Prevention, Atlanta, Georgia, USA
MOH- Ministry of Health, Kenya
LSTM- Liverpool School of Tropical Medicine

Purpose of research study
The Kenya Medical Research Institute (KEMRI), the Centers for Disease Control and Prevention (CDC), and the Liverpool School of Tropical Medicine (LSTM) are doing a research study. In addition to the main study described in the other consent form, we would like to also study how many days the drug stays in the blood. This is called a pharmacokinetic study or PK study. We will ask 36 of the 141 participants to join this extra study.

What we will do
If you agree to join this extra PK study we will admit you to the hospital ward for a total of 3 days. During these 3 days we will take a small amount of blood from you (1 teaspoon) 5 times per day. We do this by putting a small plastic needle in your arm to draw blood from (see the picture). The plastic needle will stay in your arm for 3 days. This means we should not have to prick your arm more than once. After 3 full days in the hospital you will be able to go home. However we would like you to come

back almost every day for another week (on days 4, 5, 7, 8, and 10), and then once per week for 3 more weeks (days 14, 21 and 28). This is a total of 8 visits after you leave the hospital. At each of these visits we will take some extra blood (1 teaspoon) from your arm, and 4 times also from your finger. The visits will take approximately 30 minutes.

We will put the blood into a small tube and process it in the laboratory of KEMRI. We will then ship the blood to England or America. In England /America they will tests each blood sample for the ivermectin drug level. They will test the blood for in born factors that determine how the body metabolises drugs.

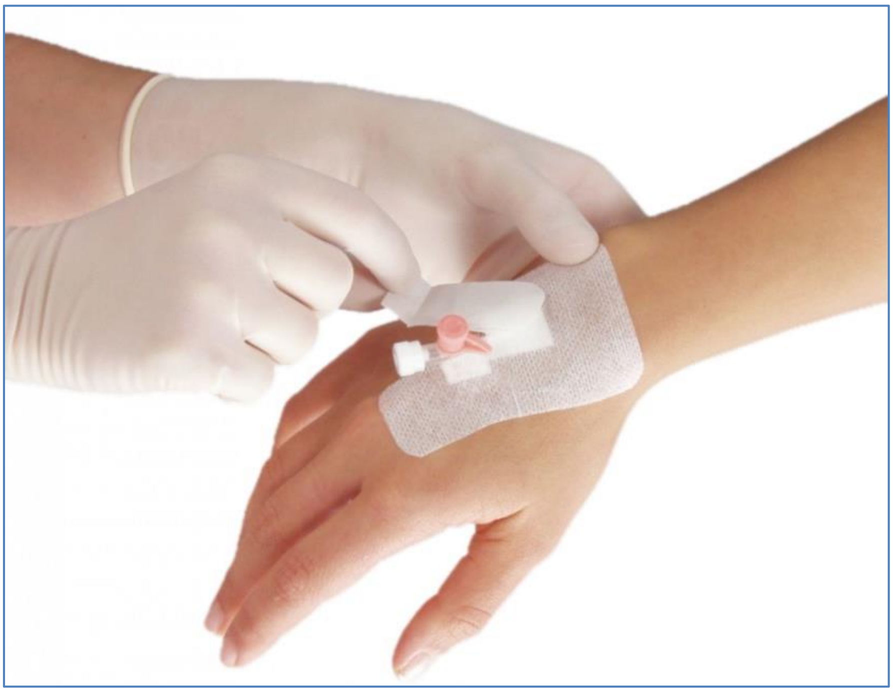

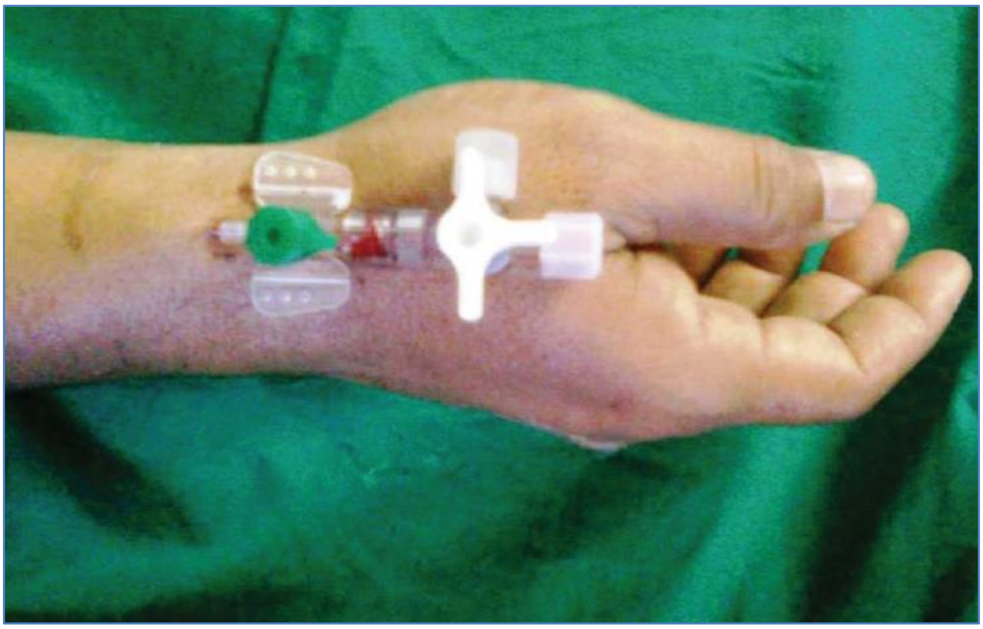

Potential benefits

There will be no potential benefit to yourself, but your contribution will help advance the science of malaria. We hope to understand how the body metabolizes ivermectin. In the future this will hopefully help to reduce malaria in western Kenya.

Potential risks

The amount of blood to be taken is too small to cause any problems. There may be itchiness at the site of plastic needle. There may be short pain when the plastic needle is inserted. There also may be a short pain when we take blood from your arm when you come back to the hospital for follow-up. There may also be a small bruise at the site of the prick. The plastic needle can sometimes also get infected but our careful procedures make this very unlikely. If it gets infected, we will have to remove it. If you agree, we will then put a new needle in another place on the same arm or in your other arm.

What else do we expect from you?

This extra study requires that you spend the next 3 days in the hospital and then come back 8 more times in the following 4 weeks. It is important for the study that patients are able and have the time to stick to the follow-up schedule.

Costs and compensation for being in the study

You will not be asked to pay anything for you to participate in this study. You will receive Ksh 500 for each day you are admitted in the hospital, plus one transport reimbursement of KSh 300-500 per day so that a family member or friend can visit you. You will also receive meals while you are admitted in the hospital. We will reimburse your transport costs for the follow-up visits on days 4, 5, 7, 8, 10, 14, 21, 28. The amount will be determined as per KEMRI/CDC guidelines. This is currently at a maximum of Ksh 300-500 every time you attend a follow-up visit.

Your rights to participate, say no, or withdraw

You are free to choose to be part of this study. You have the right to refuse. If you do not want to go on with this part of the study, or the entire study, you can stop at any time. If you choose not to participate, it will not affect the care that you will get for malaria now or whether you are allowed to participate in the main study. You will still be treated for malaria.

Contact information for questions or concerns

If you have any questions about this study, you can contact Dr Eric Ochomo at KEMRI/CDC, P.O. Box 1578-40100, Kisumu, or at 057 20 22902. You can also contact any of our study staff at the hospital. If you have any questions about your rights as a study participant, or if you want to talk about the study with someone who is not directly involved with this study, please contact The Secretary, KEMRI Scientific and Ethics Review Unit (SERU), PO Box 54840-00200, Nairobi; Telephone numbers:. Email address: seru@kemri.org. You may also contact the Secretary or Chairman of the same committee if you feel you have been harmed by this study. If you do not have access to a telephone, or you do not know how to read and write, this will not stop you from participating in this study. You may ask for contact information from the nearest health facility if you wish to talk with one of the listed individuals to raise any concerns.

If you are sick, please call Dr Eric Ochomo at KEMRI/CDC at 057 20 22902 and please go to the nearest health facility.

16.7.4. Consent Statement: Rich PK study (English)

IVERMECTIN DOSE FINDING STUDY (IVERMAL)Consent Statement PK study

Title of Study: Efficacy and safety of high-dose ivermectin for reducing malaria transmission: A dose finding study

Your signature below means that you voluntarily agree to participate in this research:

The above has been explained to me and I agree to take part in the PK study. I understand that I am free to choose to be in this study and that saying "NO" will have no effect on me. I understand that this PK study requires me to stay in the hospital for the first 3 days. I understand that small amount of blood will be taken from my arm 5 times during each of the 3 days. I understand that a plastic needle will be inserted in my arm for 3 days and that this may be slightly uncomfortable. I also understand that I will be asked to come back for follow-up visits 8 more times on days 4, 5, 7, 8, 10, 14, 21, 28. I understand that a small amount of blood will be taken during each of these visits. I also understand and agree that some of my blood may be sent to laboratories at CDC Atlanta or LSTM Liverpool or other collaborating laboratories to test drug level and for factors that may affect how my body clears drug levels. I also give permission to share the facts collected through this study, without my name and address, with other studies.	If you agree, circle "YES,"<br><br>YES

	Name	Signature or thumbprint*	Today's date
Adult providing consent for self		Signature*	
		Thumbprint*	
Witness*			
Study staff consenting participant			

* A witness is only needed if the participant cannot read. The witness must be a person independent from the study. The participant can provide a thumbprint below and verbally state his/her consent in the presence of a witness who will then sign.

16.7.5. Informed Consent Participant Information Sheet: Direct feeding (English)

IVERMECTIN DOSE FINDING STUDY
(IVERMAL)

Participant Information Sheet

Direct Skin Feeding

Title: Efficacy and safety of high-dose ivermectin for reducing malaria transmission: A dose finding study

Investigators

Dr Menno Smit, MD, MPH (KEMRI, LSTM)
Dr Eric Ochomo, PhD (KEMRI/CDC)
Dr Titus Kwambai, MD, MSc (MOH, KEMRI/CDC, LSTM) [TK]
Dr Simon Kariuki, PhD (KEMRI/CDC) [SK]
Dr Nabie Bayoh, PhD (KEMRI/CDC) [NB]
Dr Meghna Desai, PhD, MPH (KEMRI/CDC, CDC-Kenya) [MD]
Dr John Gimnig, PhD (CDC) [JG]
Dr Penelope Phillips-Howard, PhD (KEMRI, LSTM) [PPH]
Dr Aaron Samuels, MD, MHS (CDC) [AS]
Mr Ben Abong'o, MS (KEMRI/CDC)
Institutions

KEMRI- Kenya Medical Research Institute and CDC, Kisumu, Kenya
CDC- Centers for Disease Control and Prevention, Atlanta, Georgia, USA
MOH- Ministry of Health, Kenya
LSTM- Liverpool School of Tropical Medicine

Purpose of research study
The Kenya Medical Research Institute (KEMRI), the Centers for Disease Control and Prevention (CDC), and the Liverpool School of Tropical Medicine (LSTM) are doing a research study. In addition to the study described in the other consent form, we would like to know if mosquitoes that feed directly on your arm are more likely to die compared to mosquitoes that feed on blood taken from you via a needle. This is because there is likely to be a higher dose of ivermectin in skin than in your blood.

What we will do
One week from today, we will ask you to spread out your arm and then place it in a cage containing about 50 mosquitoes for about 20 minutes. The mosquitoes will bite you, which will be a little uncomfortable. It will feel similar to you getting bitten by mosquitoes in your house or outdoors. However there is no risk that you will develop an infection or malaria because the mosquitoes are reared in our laboratories and are free of disease. Further, the mosquitoes only take very small

volumes of blood. In total the mosquitoes will take less than a teaspoon of blood. We will only need to do this once during the study. On the same day we take a teaspoon of blood by a needle in your arm. We need to do this to compare the mosquito bites to the needle.

Potential benefits
There will be no potential benefit to yourself, but your contribution will help advance the science of malaria. If we discover that the needle is similar to the mosquito bites, then in the future we will hopefully no longer have to ask people to be bitten by mosquitoes in order to study malaria.

Potential risks
There may be itchiness at the site of mosquito feeding but this will disappear in a few days.

Your rights to participate, say no, or withdraw
You are free to choose to be part of this study. You have the right to refuse. If you do not want to go on with this part of the study, or the entire study, you can stop at any time. If you choose not to participate, it will not affect the care that you will get for malaria now or whether you are allowed to participate in the main study. You will still be treated for malaria.

Costs and compensation for being in the study
There will be no extra compensation compared to the standard study described in the other form. You will still receive your transport reimbursement (300-500 KSh) which you would have otherwise received for this study visit.

Contact information for questions or concerns
If you have any questions about this study, you can contact Dr Eric Ochomo at KEMRI/CDC, P.O. Box 1578-40100, Kisumu, or at 057 20 22902. You can also contact any of our study staff at the hospital. If you have any questions about your rights as a study participant, or if you want to talk about the study with someone who is not directly involved with this study, please contact The Secretary, KEMRI Scientific and Ethics Review Unit (SERU), PO Box 54840-00200, Nairobi; Telephone numbers:. Email address: seru@kemri.org. You may also contact the Secretary or Chairman of the same committee if you feel you have been harmed by this study. If you do not have access to a telephone, or you do not know how to read and write, this will not stop you from participating in this study. You may ask for contact information from the nearest health facility if you wish to talk with one of the listed individuals to raise any concerns.

If you are sick, please call Dr Eric Ochomo at KEMRI/CDC at 057 20 22902 and please go to the nearest health facility.

16.7.6. Consent Statement: Direct feeding (English)

IVERMECTIN DOSE FINDING STUDY
(IVERMAL)

Consent Statement: Direct Feeding

Title of Study: Efficacy and safety of high-dose ivermectin for reducing malaria transmission: A dose finding study

Your signature below means that you voluntarily agree to participate in this research:

The above has been explained to me and I agree to take part in the study. I understand that I am free to choose to be in this study and that saying "NO" will have no effect on me. I understand and agree that mosquitoes can feed on my arm for 20 minutes. I understand that this may be slightly uncomfortable and may result in itching of my arm for several days afterwards. I also understand that these are laboratory mosquitoes that do not carry diseases. I also give permission to share the facts collected through this study, without my name and address, with other studies.	If you agree, circle "YES,"
	YES

	Name	Signature or thumbprint*	Today's date
Adult providing consent for self		Signature*	
		Thumbprint*	
Witness*			
Study staff consenting participant			

* A witness is only needed if the participant cannot read. The witness must be a person independent from the study. The participant can provide a thumbprint below and verbally state his/her consent in the presence of a witness who will then sign.

16.7.7. Informed Consent Participant Information Sheet: Main trial (Kiswahili)

Flesch-Kincaid Reading Level: 6.7

UTAFITI WA KUPIMA KIWANGO CHA DAWA IVERMECTIN (IVERMAL)

Fomu ya maelezo ya mshiriki

Kichwa: Utafiti wa kupima kiwango cha ivermectin kilicho salama kwa kupunguza kuenea kwa ugonjwa wa malaria

Watafiti:

Dr Menno Smit, MD, MPH (KEMRI, LSTM)
Dr Eric Ochomo, PhD (KEMRI/CDC)
Dr Titus Kwambai, MD, MSc (MOH, KEMRI/CDC, LSTM) [TK]
Dr Simon Kariuki, PhD (KEMRI/CDC) [SK]
Dr Nabie Bayoh, PhD (KEMRI/CDC) [NB]
Dr Meghna Desai, PhD, MPH (KEMRI/CDC, CDC-Kenya) [MD]
Dr John Gimnig, PhD (CDC) [JG]
Dr Penelope Phillips-Howard, PhD (KEMRI, LSTM) [PPH]
Dr Aaron Samuels, MD, MHS (CDC) [AS]
Mr Ben Abong'o, MS (KEMRI/CDC)
Vituo vyao vya utafiti:

KEMRI- Kenya Medical Research Institute and CDC, Kisumu, Kenya
CDC- Centers for Disease Control and Prevention, Atlanta, Georgia, USA
MOH- Ministry of Health, Kenya
LSTM- Liverpool School of Tropical Medicine

Kusudi la utafiti

Taasisi ya Kenya Medical Research Institute (KEMRI), Center for Disease Control and Prevention (CDC), and the Liverpool School of Tropical Medical (LSTM) wanafanya utafiti. Watu nchini Kenya wanapata dawa iitwayo "dihydroartemisinin-piperaquine(DP) ili kutibu ugonjwa wa malaria, DP inawauua vimelea inayowafanya kuwa wagonjwa lakini haiwaui mbu. Ivermectin inawaua mbu wanaosambaza ugonjwa lakini haiwaui vimelea inayowafanya kuwa wagonjwa.tunawapa watu ivermectin ili kukomesha malaria isisambae kwa watu wengine. Tunawapa dawa zote mbili, DP na ivermectin, tunajua kwamba tunaweza kuzuia kuenea kwa malaria kwa muda mfupi. Tunataka kujua kama kiasi kikumbwa cha ivermectin kinaweza kuzuia kuenea kwa malaria kwa muda mrefu.

Tutakayo fanya
Uko na malaria. Tutakuuliza kama utataka kuwa mmoja wa washiriki katika mradi huu. Jibu lako likiwa "ndio, tutaangalia kama unaweza kuwa mmoja wa washirki katika mradi. Tutakuuliza maswali, kisha tuchunguze na tuchukue baadhi ya damu. Tutachunguza kiwango cha damu. Kama wewe ni mwanamke na unajulikana kwa kutokuwa na mimba, tutakuuliza idhini yako ya kuchunguza uwezekano wako wa kuwa na mimba kwa kutumia damu na mkojo. Ukipatikana kuwa na mimba au kiwango cha chini cha damu hautaruhusiwa kushiriki katika utafiti na tutakuelekeza kwa huduma bora pia kama unanyonyesha hautashiriki.

Tutakupa DP na placebo au DP na ivermectin. Tutakufuatilia ukichukua dozi ya kwanza. Tutakuuliza urejee hospitali kwa siku kumi tutakayo kupa. Kila siku ya kutembelea hospitali, mhudumu wa afya atakuuliza maswali na atachukua baadhi ya damu kutoka kwa mkono wako. Mara nne pia mhudumu atachukua damu kwenye kidole chako pia.

Hatutachukua zaidi ya 5.7 ml (inayolingana na kijiko cha chai) ya damu kwa kila siku tutachukua kiwango cha nusu kijiko ya damu. Hii ni kiwango cha chini sana. Kwa kawaidi mwili wako unarudisha bila wewe kujua.

Tutachukua picha ya moyo ili kutazama kasi ya kupiga leo, tena baada ya siku mbili na tena baada ya mwezi mmoja. Huu unaitwa ECG. Kila ECG utakuchukua Muda wa dakika kumi na tano.

Faida
Utatibiwa ugonjwa wa malaria bila malipo yoyote. Pia ivermectin itatibu minyoo, upele, kunguni na chawa. Tutakuangalia kwa ukaribu kwa matatizo ya afya wakati wa kukufuatilia. Tutakupa tiba ya hali yanayohusiana na utafiti bila malipo yoyote. Pia tutakupa nambari ya kupiga simu kama una wasiwasi wowote

Uwezekanao wa hatari
Kunawezekano wa kuwa na michubuko kwa mahali ambapo tutakuwa tumetolea damu. Dawa ya DP na ivermectin ni salama. Ivermectin inaweza kuwa na shida kwa watu wenye ugonjwa wa "loa loa". Hata hivyo "loa loa" haipatikani Kenya. Ikiwa utakuwa umetoka kwa nchi yenye "loa loa", basi kuna uwezekana ya wewe kutoshiriki kwa utafiti.

Faragha na usiri
Taarifa kukuhusu utakuwa siri kiwango cha juu cha kwamba sheria itakubali. Taarifa tuliyokusanya kuhifadhiwa na kulindwa kwa masanduku yenye kufuli na kompyuta inayofunguliwa na watu wenye idhini pekee ilioko kwa afisi za KEMRI/CDC, Kisumu. Tutatumia taarifa utakayotupa kwa utafiti pekee. Jina lako halitakuwa kwa ripoti yoyote. Mwisho wa utafiti, tutatoa jina lako kwa karatasi za utafiti ili taarifa yanayokuhusu au aina ya damu yako kutojulikana kwa mtu mwingine.

Haki zako za kushiriki, kukataa au kukubali
Unauhuru wa kuchagua kuwa mmoja wa washiriki wa mradi huu. Pia una uhuru wa kukataa. Ikiwa hutaki kuendelea na mradi huu, unaweza kusimamisha wakati wowote. Ikiwa utachagua kutoshiriki, haiwezi kuathiri huduma utakazopata za malaria. Utaendelea kutibiwa kutokana na malaria.

Gharama na fidia kwa kuwa mradi

Hautaulizwa kulipa chochote ili kuwa kwa mradi huu. Nauli zote za shughuli uliopangwa kufanyika au ziara ya hospitali, zitatolewa kulingana na miongozo wa KEMRI/CDC kwa sasa upeo wa 300-500 kwa kila ziara.

Ridhaa ya muda mrefu kwa ifadhi ya sampuli ya damu kwa utafiti za baadaye

Tunawauliza watu watakaojiunga na utafiti kama watakubalia wachunguzi kutumia sampuli ya damu zao kwa utafiti wa baadaye. Utafiti huu wa baadaye unaweza saidia kupata njia mapya ya kuzuia malaria. Ikiwa utakubali tutahifadhi damu yako ikiwa na nambari za kipekee lakini si jina lako kwa eneo salama katika maabara za KEMRI/CDC. Tunaweza kushiriki matokeo ya uchunguzi wa malaria kwa wachunguzi wa mashirika mengine lakini hatutawapa majina yako, anwani au taarifa mengine ambayo inaweza kukujulisha. Baadaya muda wa utafiti kuisha tutatoa taarifa yoyote ambayo unaweza kuambatanisha sampuli na wewe. Ikiwa hutataka damu yako ihifadhiwe, unaweza bado kushiriki katika utafiti. Utachunguzwa ugonjwa wa malaria na kiwango kidogo ya damu.

Mawasiliano ya habari kwa maswali na wasiwasi

Ikiwa una maswali yoyote kuhusu mradi huu, Piga simu kwa Dr Eric Ochomo akiwa KEMRI/CDC, Sanduku la Posta 1578-40100, Kisumu, au kwa 057 20 22902. Pia unaweza kuwasiliana na wafanyikazi wa utafiti huu katika hospitali. Ikiwa una maswali kuhusu haki zako kama mmoja wa washiriki katika utafiti huu, au kama unataka kuongea kuhusu utafiti na mtu ambaye hana huusiano wa moja kwa moja kwa utafiti, tafadhali wasiliana na na katibu wa KEMRI Scientific and Ethics Review Unit (SERU), Sanduku la Posta 54840-00200, Nairobi, Simu 020 2722541, 0722205901 au 0733400003. Barua pepe seru@kemri.org. Pia unaweza kuwasiliana na katibu au mkuu wa kamati ukihisi kuwa umeumizwa na mradi huu. Kama huna simu au hujui kusoma au kuandika, hii haiwezi kukuzuia kwa kushiriki katika mradi huu. Unaweza kuuliza habari ya mawasiliano kutoka kituo cha afya iliokaribu nawe, unaweza kuuliza habari ya mawasiliano kutoka kwa watu waliotajwa kuhusu wasiwasi wowote.

Ukihisi mgonjwa, mpigie simu Dr. Eric Ochomo wa KEMRI/CDC kwa nambari ya simu 057 20 22902 na uende kwenye kituo cha afya kilicho karibu nawe, na uenda kituo cha afya ilio karibu nawe

Asante sana kwa muda wote. Ikiwa utakubali kushiriki kwa mradi huu, tutakuuliza utie sahihi kwa fomu hii.

16.7.8. Consent Statement: Main trial (Kiswhili)

UTAFITI WA KUPIMA KIWANGO CHA DAWA IVERMECTIN (IVERMAL)

Kauli ya ridhaa

Kichwa ya uchunguzi: Utafiti wa kupima kiwango cha ivermectin kilicho salama kwa kupunguza kuenea kwa ugonjwa wa malaria

Sahihi yako hapo chini inamaanisha kuwa umekubali kwa hiari kushiriki/kuwa mtoto wako kushiriki katika uchunguzi huu wa utafiti:

Maelezo ya hapo juu nimeyaelewa na nimekubali kuchukua sehemu katika utafiti huu.Nimeelewa kuwa nina uhuru wa kuchagua kuwa kwa mradi huu na kwa kukataa haita kuwa na athari zozote kwangu.Ninakubali damu kupimwa malaria na kiwango cha chini.Pia ninaelewa kuwa sehemu ya damu yangu itapelekwa maabara za CDC Atlanta au LSTM Liverpool mahabara zinginezo zinazohusika kwa uchunguzi ambao hautafanyika Kenya.Ninaelewa kuwa sehemu muhimu ya kumbukumbu ya afya yangu inaweza kuangaliwa na wafanyikazi wa KEMRI/CDC.Ninatoa ruhusa kwa watu hawa kupata kumbukumbu zangu.pia ninatoa ruhusa kushiriki ukweli uliokusanywa wakati wa utafiti bila jina na anwani na utafiti zingine.	Ikiwa unakubali tia mviringo “Ndiyo”<br><br>NDIYO

	Jina	Sahihi	Tarehe ya leo
Mtu mzima anayepeana ridhaa kwake mwenyewe		Sahihi	
		Alama ya kidole cha gumba	
Shahidi			
Mfanyikazi wa mradi anayetoa ridhaa kwa mshirika			

*Shahidi anaitajika ikiwa mshiriki hawezi kusoma.Lazima shahidi awe asiye na uhusiano wowote na utafiti.mshirika anaweza kutia sahihi au kusema kwa hali ya maneno ridhaa yake mbele ya shahidi atakaye tia sahihi

UTAFITI WA KUPIMA KIWANGO CHA DAWA IVERMECTIN (IVERMAL)

Ridhaa ya kuhifadhi sampluli ya damu kwa muda mrefu kwa uchunguzi wa baadaye

Kichwa ya uchunguzi: Utafiti wa kupima kiwango cha ivermectin kilicho salama kwa kupunguza kuenea kwa ugonjwa wa malaria

Sahihi yako hapo chini inamaanisha kuwa umekubali kwa hiari kushiriki/kuwa mtoto wako kushiriki katika uchunguzi huu wa utafiti:

<table>
<tr><td rowspan="2">Nimeelezwa kuhifadhiwa kwa damu yangu na ninakubalia KEMRI/CDC kuhifadhi damu yangu kwa muda ya miaka 15 au zaidi kwa utafiti wa baadaye.pia ninakubali kuwa damu yangu inaweza kutumwa katika maabara za CDC Atlanta au LSTM Liverpool mahabara zinginezo zinazohusika kwa uchunguzi ambayo haitafanywa nchini Kenya.Pia ninaelewa kuwa ninaweza kubadili mawazo yako kutoruhusu sampuli ya damu yangu kutumwa,kuhifadhiwa au kutumiwa kwa utafiti wa baadaye.Ili kufanya hivi ninaweza kumweleza Dr Eric Ochomo kuhusu ombi langu na atawaambia wafanyikazi wengine wa KEMRI/CDC</td><td colspan="2">Ikiwa utakubali zingira “NDIO”,kama utakataa,zingira”LA”</td></tr>
<tr><td>NDIYO</td><td>LA</td></tr>
</table>

	Jina	Sahihi	Tarehe ya leo
Mtu mzima anayepeana ridhaa		Sahihi	
		Alama ya kidole cha gumba	
Shahidi			
Mfanyikazi wa utafiti anayepeana ridhaa			

*Shahidi anaitajika ikiwa mshiriki hawezi kusoma.Lazima shahidi awe asiye na uhusiano wowote na utafiti.mshirika anaweza kutia sahihi au kusema kwa hali ya maneno ridhaa yake mbele ya shahidi atakaye tia sahihi

16.7.9. Informed Consent Participant Information Sheet: Rich PK study (Kiswahili)

UTAFITI WA KUPIMA KIWANGO CHA DAWA IVERMECTIN (IVERMAL)

Fomu ya maelezo ya mshiriki

Kichwa: Utafiti wa kupima kiwango cha ivermectin kilicho salama kwa kupunguza kuenea kwa ugonjwa wa malaria

Investigators

Dr Menno Smit, MD, MPH (KEMRI, LSTM)
Dr Eric Ochomo, PhD (KEMRI/CDC)
Dr Titus Kwambai, MD, MSc (MOH, KEMRI/CDC, LSTM) [TK]
Dr Simon Kariuki, PhD (KEMRI/CDC) [SK]
Dr Nabie Bayoh, PhD (KEMRI/CDC) [NB]
Dr Meghna Desai, PhD, MPH (KEMRI/CDC, CDC-Kenya) [MD]
Dr John Gimnig, PhD (CDC) [JG]
Dr Penelope Phillips-Howard, PhD (KEMRI, LSTM) [PPH]
Dr Aaron Samuels, MD, MHS (CDC) [AS]
Mr Ben Abong'o, MS (KEMRI/CDC)
Institutions

KEMRI- Kenya Medical Research Institute and CDC, Kisumu, Kenya
CDC- Centers for Disease Control and Prevention, Atlanta, Georgia, USA
MOH- Ministry of Health, Kenya
LSTM- Liverpool School of Tropical Medicine

Kusudi la utafiti

Taasisi ya Kenya Medical Research Institute (KEMRI), Center for Disease Control and Prevention(CDC),and the Liverpool School of Tropical Medical (LSTM) wanafanya utafiti. Pamoja na utafiti mkuu ulioelezwa kwenye fomu lile lingine la maelezo kwa mshiriki, tungependa pia kujua ni siku ngapi dawa itakaa kwenye damu yako. Huu unaitwa kwa kimombo, 'Pharmacokinetic study'. Tutawauliza washiriki 36 kati ya wote 141 kujisajili kwa utafiti huu.

Tutakayo fanya

Ukikubali kujiunga na utafiti huu wa kuchunguza hati ya ivermectin kwenye damu, tutakulaza kwenye wodi ya hospitali kwa siku tatu. Katika siku hizi tatu, tutachukua kiasi kidogo cha damu (inayolingana na kijiko cha chai) mara 5 kwa siku. Tutafanya hivi kwa kutumia sindano kidogo ya plastiki kutoa damu kutoka kwenye mkono wako (tazama picha). Sindano hiyo itakaa mkononi mwako kwa siku tatu ili tusikudunge sindano zaidi ya mara moja. Baada ya siku tatu hospitalini, tutakuruhusu kwenda nyumbani. Lakini tutakuuliza uweze kurudi hospitalini kwa karibu kila siku wiki ya kwanza (siku ya 4, 5, 7, 8, and 10) halafu tena mara moja kila wiki kwa wiki tatu (siku ya 14, 21 na 28). Kwa pamoja, tutakuhitaji kurudi hospitalini mara 8 baada ya kutoka hospitalini. Kila utakaporudi

hospitalini, tutachukua damu kidogo (inayolingana na kijiko cha chai) kutoka mkononi mwako, na mara nne pia kutoka kwa kidole chako. Kila utakapokuja hospitalini tutachukua muda usiozidi nusu saa.

Tutaweka damu yako kwenye chombo kidogo na kuifanyia utafiti kwenye maabara yetu ya KEMRI. Halafu tutatuma damu hii hadi Uingereza ambapo kila sampuli ya damu itachunguzwa kupima kiwango cha ivermectin. Pili tofauti za kigenetiki zinazoamua jinsi mwili unavyo tumia dawa.

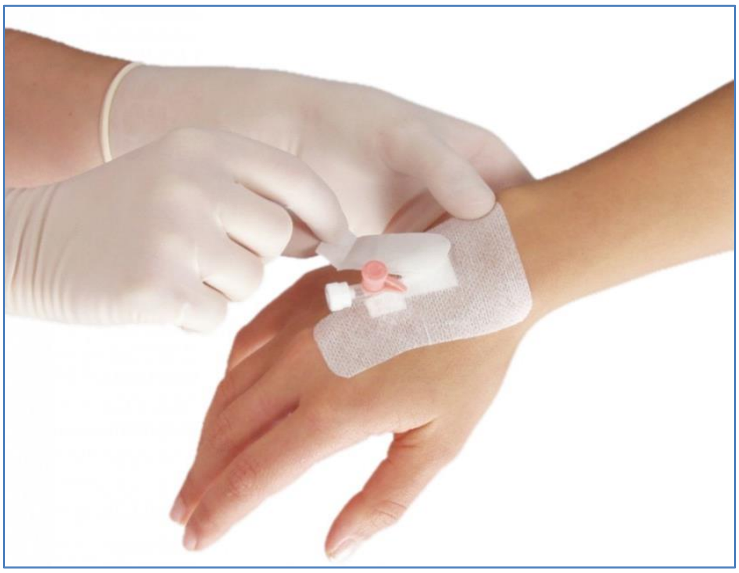

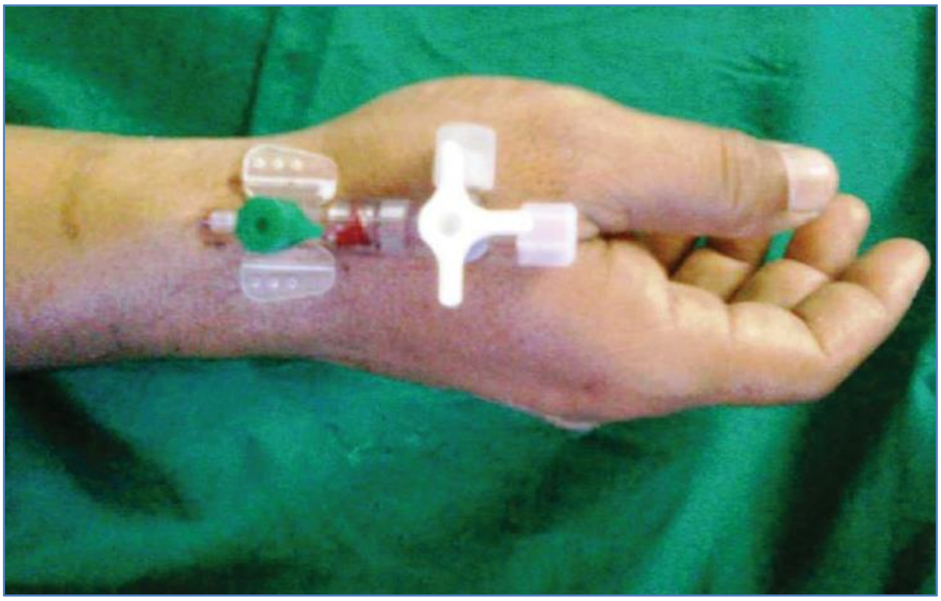

Faida

Hakutakuwa na faida ya moja kwa moja kwako kama mhusika, ila kujihusisha kwako kutatuwezesha kuendeleza ujuzi kuhusu sayansi ya malaria. Utafiti huu utatuwezesha kuelewa jinsi mwili unavyo tumia ivermectin. Mbeleni, huu utatuwezesha kupunguza malaria magharibi mwa Kenya.

Uwezekanao wa hatari

Damu tutakayochukua ni kiwango kidogo ambacho hakiwezi kusababisha taabu yoyote. Waweza kuwashwa na kuhisi kujikuna sehemu ya kudungwa sindano. Pia kutaweza kuwa na uchungu kidogo

wakatu tunapoingiza sindano. Waweza pia kuhisi uchungu kidogo tutakapochukua damu kidogo wakati utakapokuwa ukirudi hospitalini. Waweza pia kuwa na mkwaruzi mdogo kwenye eneo utakapodungwa sindano. Wakati mwingine sindano ya plastiki yaweza kupata viini vya ugonjwa lakini tutakuangalia kwa makini kuhakikisha hiyo haifanyiki. Lakini ikafanyika, tutautoa sindano huo na kuomba kubadilisha kwa mkono mwingine.

Ni nini tena tunataraji kutoka kwako?
Utafiti huu utakuhitaji kulazwa hospitalini kwa muda wa siku tatu halafu urudi tena hospitalini mara 8 katika wiki nne zitakazofuata. Ni muhimi kwako kama mshiriki kuzingatia masaa na tarehe utakazopewa ili kurudi hospitalini.

Gharama na fidia kwa kuwa mradi
Hutaulizwa kulipa chochote ili kujiunga na utafiti huu. Kila siku utakapolazwa hospitalini, tutakupa shilingi 500 na nyingine 300-500 ili jamaa wako waweze kukutembelea. Pia tutakuwa tunakurudishia pesa utakazotumia kuja hospitalini kwa kila siku utakaporudi hospitalini siku ya 4, 5, 7, 8, 10, 14, 21, 28. Kiwango cha fidia kitaamuliwa kufuatana na sharia na masharti ya KEMRI/CDC. Kwa sasa kiwango hiki ni shilingi 300-500 kwa kila utakapokuja hospitalini.

Haki zako za kushiriki,kukataa au kukubali
Unauhuru wa kuchagua kuwa mmoja wa washiriki wa mradi huu. Pia una uhuru wa kukataa. Ikiwa hutaki kuendelea na mradi huu,unaweza kusimamisha wakati wowote. Ikiwa utachagua kutoshiriki,haiwezi kuathiri huduma utakazopata za malaria. Utaendelea kutibiwa kutokana na malaria

Faragha na usiri
Taarifa kukuhusu utakuwa siri kiwango cha juu cha kwamba sheria itakubali. Taarifa tuliyokusanya kuhifadhiwa na kulindwa kwa masanduku yenye kufuli na kompyuta inayofunguliwa na watu wenye idhini pekee ilioko kwa afisi za KEMRI/CDC,Kisumu. Tutatumia taarifa utakayotupa kwa utafiti pekee. Jina lako halitakuwa kwa ripoti yoyote. Mwisho wa utafiti,tutatoa jina lako kwa karatasi za utafiti ili taarifa yanayokuhusu au aina ya damu yako kutojulikana kwa mtu mwingine

Mawasiliano ya habari kwa maswali na wasiwasi
Ikiwa una maswali yoyote kuhusu mradi huu,Piga simu kwa Dr Eric Ochomo akiwa KEMRI/CDC,Sanduku la Posta 1578-40100,Kisumu,au kwa 057 20 22902. Pia unaweza kuwasiliana na wafanyikazi wa utafiti huu katika hospitali..Ikiwa una maswali kuhusu haki zako kama mmoja wa washiriki katika utafiti huu,au kama unataka kuongea kuhusu utafiti na mtu ambaye hana huusiano wa moja kwa moja kwa utafiti,tafadhali wasiliana na na katibu wa KEMRI Scientific and Ethics Review Unit (SERU),Sanduku la Posta 54840-00200,Nairobi,Simu 020 2722541,0722205901 au 0733400003.barua pepe seru@kemri.org. Pia unaweza kuwasiliana na katibu au mkuu wa kamati ukihisi kuwa umeumizwa na mradi huu. Kama huna simu au hujui kusoma au kuandika,hii haiwezi kukuzuia kwa kushiriki katika mradi huu. Unaweza kuuliza habari ya mawasiliano kutoka kituo cha afya iliokaribu nawe,unaweza kuuliza habari ya mawasiliano kutoka kwa watu waliotajwa kuhusu wasiwasi wowote.

Ukihisi mgonjwa, mpigie simu Dr. Eric Ochomo wa KEMRI/CDC kwa nambari ya simu 057 20 22902 na uende kwenye kituo cha afya kilicho karibu nawe.

16.7.10. Consent Statement: Rich PK study (Kiswahili)

UTAFITI WA KUPIMA KIWANGO CHA DAWA IVERMECTIN (IVERMAL)

Kauli ya ridhaa ya uchunguzi wa kiasi cha dawa kilicho kwenye damu (PK)

Kichwa ya uchunguzi: Utafiti wa kupima kiwango cha ivermectin kilicho salama kwa kupunguza kuenea kwa ugonjwa wa malaria

Sahihi yako hapo chini inamaanisha kuwa umekubali kwa hiari kushiriki/kuwa mtoto wako kushiriki katika uchunguzi huu wa utafiti:

Nimeelezewa yaliyo hapo juu na ninakubali kushiriki katika uchunguzi wa kiasi cha dawa kilicho kwenye damu (PK). Ninaelewa niko na uhuru wa kuchaguwa kuwa katika uchunguzi huu na kusema “Hapana” haitakuwa na madhara kwangu. Ninaelewa ya kuwa uchunguzi huu wa PK inanihitaji niwe hospitalini siku 3 za kwanza. Ninaelewa kuwa damu kidogo itachukuliwa kutoka kwa mkono wangu mara 5 kwa siku katika hizo siku 3. Ninaelewa kuwa sindano ya plastiki itawekwa kwenye mkono wangu kwa siku tatu na hii itakuwa na usumbufu kidogo. Ninaelewa ya kuwa nitaulizwa kurejea kwa ufwatilio mara 8 zaidi siku ya 4, 5, 7, 8, 10, 14, 21, 28. Ninaelewa kuwa damu kidogo itachukuliwa katika kila ya marejeo haya. Nina na ninakubali kuwa baadhi ya damu yangu inaweza kutumwa katika maabara ya CDC Atlanta au LSTM Liverpool au maabara mengine yanayoshirikiana kupima kiwango cha dawa na mambo yanayo dhuru jinsi mwili wangu husafisha kiwango cha dawa. Ninatoa ruhusa kuonyeshana na wengine ukweli uliokusanyiwa kupitia kwa uchunguzi huu, bila jina langu na anwani na uchunguzi mwingine.	Ikiwa unakubali tia mviringo “Ndiyo”<br><br>NDIYO

	Jina	Sahihi	Tarehe ya leo
Mtu mzima anayepeana ridhaa kwake			
		Alama ya kidole cha	
Shahidi			
Mfanyikazi wa mradi anayetoa ridhaa kwa			

*Shahidi anaitajika ikiwa mshiriki hawezi kusoma.Lazima shahidi awe asiye na uhusiano wowote na utafiti.mshirika anaweza kutia sahihi au kusema kwa hali ya maneno ridhaa yake mbele ya shahidi atakaye tia sahihi

16.7.11. Informed Consent Participant Information Sheet: Direct feeding (Kiswahili)

UTAFITI WA KUPIMA KIWANGO CHA DAWA IVERMECTIN (IVERMAL)

Fomu ya maelezo ya mshiriki

Kichwa: Utafiti wa kupima kiwango cha ivermectin kilicho salama kwa kupunguza kuenea kwa ugonjwa wa malaria

Watafiti:

Dr Menno Smit, MD, MPH (KEMRI, LSTM)
Dr Eric Ochomo, PhD (KEMRI/CDC)
Dr Titus Kwambai, MD, MSc (MOH, KEMRI/CDC, LSTM) [TK]
Dr Simon Kariuki, PhD (KEMRI/CDC) [SK]
Dr Nabie Bayoh, PhD (KEMRI/CDC) [NB]
Dr Meghna Desai, PhD, MPH (KEMRI/CDC, CDC-Kenya) [MD]
Dr John Gimnig, PhD (CDC) [JG]
Dr Penelope Phillips-Howard, PhD (KEMRI, LSTM) [PPH]
Dr Aaron Samuels, MD, MHS (CDC) [AS]
Mr Ben Abong'o, MS (KEMRI/CDC)
Vituo vyao vya utafiti:

KEMRI- Kenya Medical Research Institute and CDC, Kisumu, Kenya
CDC- Centers for Disease Control and Prevention, Atlanta, Georgia, USA
MOH- Ministry of Health, Kenya
LSTM- Liverpool School of Tropical Medicine

Kusudi la utafiti

Taasisi ya Kenya Medical Research Institute (KEMRI), Center for Disease Control and Prevention(CDC), and the Liverpool School of Tropical Medical (LSTM) wanafanya utafiti. Pamoja na utafiti ulioelezwa kwenye fomu lile lingine, tungependa kujua kama mbu wanaofyonza damu mkononi mwako wanaathirika zaidi na dawa ya ivermectin kuliko wale ambao tutawalisha damu tutakayochukua kwako kwa kutumia sindano. Hii ni kwasababu kuna kipimo kikubwa cha dawa ya ivermectin kwenye ngozi na damu kuliko kwenye damu pekee.

Tutakayo fanya

Siku ya saba baada ya kuanza matibabu, tutakuuliza uingize mkono wako kwenye kizuizi cha mbu iliyo na mbu 50 ili kuwalisha kwa muda wa dakika 20. Wakati watakapokudunga utahili kukosa starehe kidogo tu lakini mbu hao wamelelewa kwenye maabara yetu na hawana maradhi yoyote kwa hivyo hawatakusambazia magonjwa yoyote. Mbu hufyonza kiasi kidogo sana cha damu na mbu hao wote hawatafyonza zaidi ya kijiko kimoja kidogo ya damu.

Faida
Hakutakuwa na faida ya moja kwa moja kwako kama mhusika, ila kujihusisha kwako kutatuwezesha kuendeleza ujuzi kuhusu sayansi ya malaria.

Uwezekanao wa hatari
Kunawezekano wa kujikuna mahali ambapo mbu watakuwa wamedunga wanapofyonza damu.

Faragha na usiri
Taarifa kukuhusu utakuwa siri kiwango cha juu cha kwamba sheria itakubali. Taarifa tuliyokusanya kuhifadhiwa na kulindwa kwa masanduku yenye kufuli na kompyuta inayofunguliwa na watu wenye idhini pekee ilioko kwa afisi za KEMRI/CDC,Kisumu. Tutatumia taarifa utakayotupa kwa utafiti pekee. Jina lako halitakuwa kwa ripoti yoyote. Mwisho wa utafiti,tutatoa jina lako kwa karatasi za utafiti ili taarifa yanayokuhusu au aina ya damu yako kutojulikana kwa mtu mwingine

Haki zako za kushiriki,kukataa au kukubali
Unauhuru wa kuchagua kuwa mmoja wa washiriki wa mradi huu. Pia una uhuru wa kukataa. Ikiwa hutaki kuendelea na mradi huu, unaweza kusimamisha wakati wowote. Ikiwa utachagua kutoshiriki,haiwezi kuathiri huduma utakazopata za malaria. Utaendelea kutibiwa kutokana na malaria.

Gharama na fidia kwa kuwa mradi
Hautaulizwa kulipa chochote ili kuwa kwa mradi huu. Nauli zote za shughuli uliopangwa kufanyika au ziara ya hospitali,zitatolewa kulingana na miongozo wa KEMRI/CDC kwa sasa upeo wa 300-500 kwa kila ziara.

Ridhaa ya muda mrefu kwa ifadhi ya sampuli ya damu kwa utafiti za baadaye
Tunawauliza watu watakaojiunga na utafiti kama watakubalia wachunguzi kutumia sampuli ya damu zao kwa utafiti wa baadaye. Utafiti huu wa baadaye unaweza saidia kupata njia mapya ya kuzuia malaria. Ikiwa utakubali tutahifadhi damu yako ikiwa na nambari za kipekee lakini si jina lako kwa eneo salama katika maabara za KEMRI/CDC. Tunaweza kushiriki matokeo ya uchunguzi wa malaria kwa wachunguzi wa mashirika mengine lakini hatutawapa majina yako, anwani au taarifa mengine ambayo inaweza kukujulisha. Baadaya muda wa utafiti kuisha tutatoa taarifa yoyote ambayo unaweza kuambatanisha sampuli na wewe. Ikiwa hutataka damu yako ihifadhiwe, unaweza bado kushiriki katika utafiti. Utachunguzwa ugonjwa wa malaria na kiwango kidogo ya damu.

Mawasiliano ya habari kwa maswali na wasiwasi
Ikiwa una maswali yoyote kuhusu mradi huu, Piga simu kwa Dr Eric Ochomo akiwa KEMRI/CDC, Sanduku la Posta 1578-40100, Kisumu, au kwa 057 20 22902. Pia unaweza kuwasiliana na wafanyikazi wa utafiti huu katika hospitali. Ikiwa una maswali kuhusu haki zako kama mmoja wa washiriki katika utafiti huu, au kama unataka kuongea kuhusu utafiti na mtu ambaye hana huusiano wa moja kwa moja kwa utafiti, tafadhali wasiliana na na katibu wa KEMRI Scientific and Ethics Review Unit (SERU),Sanduku la Posta 54840-00200, Nairobi, Simu 020 2722541, 0722205901 au 0733400003. Barua pepe seru@kemri.org. Pia unaweza kuwasiliana na katibu au mkuu wa kamati ukihisi kuwa umeumizwa na mradi huu. Kama huna simu au hujui kusoma au kuandika, hii haiwezi kukuzuia kwa kushiriki katika mradi huu. Unaweza kuuliza habari ya mawasiliano kutoka kituo cha afya iliokaribu nawe, unaweza kuuliza habari ya mawasiliano kutoka kwa watu waliotajwa kuhusu wasiwasi wowote.

Ukihisi mgonjwa, mpigie simu Dr. Eric Ochomo wa KEMRI/CDC kwa nambari ya simu 057 20 22902 na uende kwenye kituo cha afya kilicho karibu nawe, na enda kituo cha afya ilio karibu nawe
Asante sana kwa muda wote. Ikiwa utakubali kushiriki kwa mradi huu, tutakuuliza utie sahihi kwa fomu hii.

16.7.12. Consent Statement: Direct feeding (Kiswhili)

UTAFITI WA KUPIMA KIWANGO CHA DAWA IVERMECTIN (IVERMAL)

Kauli ya ridhaa

Kichwa ya uchunguzi: Utafiti wa kupima kiwango cha ivermectin kilicho salama kwa kupunguza kuenea kwa ugonjwa wa malaria

Sahihi yako hapo chini inamaanisha kuwa umekubali kwa hiari kushiriki/kuwa mtoto wako kushiriki katika uchunguzi huu wa utafiti:

Maelezo ya hapo juu nimeyaelewa na nimekubali kuchukua sehemu katika utafiti huu. Nimeelewa kuwa nina uhuru wa kuchagua kuwa kwa mradi huu na kwa kukataa haita kuwa na athari zozote kwangu. Ninakubali damu kupimwa malaria na kiwango cha chini. Pia ninaelewa kuwa sehemu ya damu yangu itapelekwa maabara za CDC Atlanta au LSTM Liverpool mahabara zinginezo zinazohusika kwa uchunguzi ambao hautafanyika Kenya. Ninaelewa kuwa sehemu muhimu ya kumbukumbu ya afya yangu inaweza kuangaliwa na wafanyikazi wa KEMRI/CDC. Ninatoa ruhusa kwa watu hawa kupata kumbukumbu zangu. Pia ninatoa ruhusa kushiriki ukweli uliokusanywa wakati wa utafiti bila jina na anwani na utafiti zingine.	Ikiwa unakubali tia mviringo "Ndiyo"<br><br>NDIYO

	Jina	Sahihi	Tarehe ya leo
Mtu mzima anayepeana ridhaa kwake mwenyewe		Sahihi	
		Alama ya kidole cha gumba	
Shahidi			
Mfanyikazi wa mradi anayetoa ridhaa kwa mshirika			

*Shahidi anaitajika ikiwa mshiriki hawezi kusoma. Lazima shahidi awe asiye na uhusiano wowote na utafiti. mshirika anaweza kutia sahihi au kusema kwa hali ya maneno ridhaa yake mbele ya shahidi atakaye tia sahihi.

16.7.13. Informed Consent Participant Information Sheet: Main trial (Dholuo)

NONRO MAR YUDO PIM MAR YATH IVERMECTIN (IVERMAL)

Oboke ma otingo weche jachiwre e nonro

Wi nonro: Nonro mar yudo pim mar yath ma iluongo ni Ivermectin manyalo geng'o landruok mar tuo mar malaria

Jo ta nonro:

Dr Menno Smit, MD, MPH (KEMRI, LSTM)
Dr Eric Ochomo, PhD (KEMRI/CDC)
Dr Titus Kwambai, MD, MSc (MOH, KEMRI/CDC, LSTM) [TK]
Dr Simon Kariuki, PhD (KEMRI/CDC) [SK]
Dr Nabie Bayoh, PhD (KEMRI/CDC) [NB]
Dr Meghna Desai, PhD, MPH (KEMRI/CDC, CDC-Kenya) [MD]
Dr John Gimnig, PhD (CDC) [JG]
Dr Penelope Phillips-Howard, PhD (KEMRI, LSTM) [PPH]
Dr Aaron Samuels, MD, MHS (CDC) [AS]
Mr Ben Abong'o, MS (KEMRI/CDC)
Kuonde ma gitiye:

KEMRI- Kenya Medical Research Institute and CDC, Kisumu, Kenya
CDC- Centers for Disease Control and Prevention, Atlanta, Georgia, USA
MOH- Ministry of Health, Kenya
LSTM- Liverpool School of Tropical Medicine

Gima miyo watimo nonro

Kar timo nonro mar Kemri ka oriwore gi CDC kod Liverpool School of Tropical Medicine timo nonro. Jo Kenya tiyo gi yath ma iluongo ni dihydroartemisinin-piperaquine (DP) thiedho tuo mar malaria. DP nego kute mamiyo ibedo matuo. DP ok neg suna. Yath ma iluongo ni ivermectin to nego suna makelo tuo mar malaria to ok oneg kute makelo malaria mamiyo ibedo matuo. Wachiwo ne ji yath mar ivermectin magen'go malaria mondo kik landre kuom ji ma moko. Ka wachiwo DP kod ivermectin, wan'geyo ni wanyalo duoko landruok mar malaria kuom ndalo manok. Wadwaro nono ka bende ka wachiwo yath mar ivermectin mangeny matin to bende nyalo gen'go landruok mar malaria kuom ndalo man'geny.

Kaka wabiro timo:

In gi malaria. Wabiro penji ka bende diher bedo e nonro ni. Ka iyie, wabiro ng'iyo ka bende inyalo bedo e nonro. Wabiro penji penjo ma moko, wabiro pimo rembi ka bende in gi remo ma oromo. Ka in mg'ama miyo, ma ok pek, wabiro pimo lach kata remo mari ng'iyo ka bende ipek, ka wayudo ni ipek kata rembi tin to ok inyal bedo e nonro wabiro ori kar thieth mani machiegni kodi. Kendo ka idhodho

bende ok ibibedo e nonro. Wabiro miyi DP kod yedhe ma moko kata DP kod ivermectin. Wabiro ngiyo ka imwonyo yath ma kuongo. Wabiro kwayo ni ibi duog kar thieth kuom limbe 10 ma wabiro chiwo tarik. Kuom limbe 10 go, jathieth biro penji penjo kendo kawo rembi ka wagolo e lweti. Kuom limbe 4 jathieth bende biro kawo remo e lith lweti. Remo ok bikwaw ma okalo 5.7 ml (ojiko matin). Kinde ma wakawo to wabiro kawo remo matin ne nus ojiko matin. Mano remo matin ndi ma dendi duoko piyo ma okibingeyo. Wabiro goyo adundo ni picha mar mondo wa non godo gocho mare kawuono kendo bang' ndalo ariyo bang'e kendo bang' dwe achiel ka oa kawuono. Mae iluongo ni ECG kendo moro ka moro biro kawo dakika apar kod abich.

Ber manitie

Ibiro thiedhi ka in gi tuo mar malaria maonge chudo mora mora. Yath mar ivermectin thiedho njofni, gwonyo, chwarni, kod onyuogo. Wabiro limi ka wang’iyo kaka idhi bang muonyo yadh no. Ibiro thiedhi ne touché ma okel gi yedhe ka dibed ni nitie, nono maonge chudo. Namba simu bende wabiro chiwo ma kadibed gi wach kata penjo to inyalo gocho saa asaya.

Rach manitie

Inyalo bedo gi alama matin kama wachwoyo ka igloo remo. Yedhe ma watiyo godo gi yedhe ma opwodhi kendo longo. Ivermectin nyalo bedo gi rach kuom joma ni gi tuo ma iluongo ni loa loa. Mak mana ni tuo mar loa loa to onge Kenya ka. Ka dibed ni isedhi e pinje ma ni gi tuo ni to kik ibedie nonro ni.

Kano weche ma Opondo

Weche ibiro kan ma Opondo kaka chik owacho. Ripode ma wabiro ndiko biro kan maber e ofi Kemri/CDC e kabade ma iloro kendo e kompyuta ma onge ngata ngata ma okowinjire neon mabiro neno. Weche mabiro wuok e nonro ibiro ti godo mana e nonro ni kendo nyinge u ok bi wuoke ripode moka moka ma ibiro gol. Bang nonro to nyinge duto ibiro gol maonge ngato mabiro ngeyo weche gi kata rembi.

Thuolo kod ratiro mari mar bedo e nonro kata wuok

In thuolo mar bedo e nonro ni. Kendo en ratiro mari tamori ka ok idwar. Bende kata ka isedonjo e nonro to idwaro wuok, in huolo. Ka idagi bende pod en ratiro mari yudo thieth mar malaria ma ibiro chiw.

Chudo kata mich e nonro

Ok bi kwayi ni ichul pesa mora mora mondo eka idonj e nonro ni. Ka itiyo gi pesa ni dhi kar thieth e ndalo ma wawinjore to ibiro duok ni pesa no kaka owinjore gi Kemri/CDC. Pesa kind 300-500 e ndalo ma idhi kar thieth.

Thuolo mar kano remo ma okaw kuom ndalo mang’eny ne wach nonro ma moko manyalo bedo:

Wakwayo ji ma odonjo e nonro ka bende wanyalo kano remo ma gi chiwo ne nonro ma moko e ndalo mabiro. Nonro ma moko gi gin mek konyo kaka wanyalo geng’o malaria. Ka iyie, to wabiro kano remo no gi namba ma opogore gi nyingi ma ok wabi tiyo gi nyingi. Kendo ibiro kane kama diny. Ripode ma wayudo kuom remo wanyalo ng’iso jo ma moko ma watudore go mak mana ni ok wabitiyo gi nyingi kata adres mari kata gima nyalo fulo ni en in. Ka nonro oserumo, to wabiro golo gik moko te manyalo nyiso ni en in mane nie nonro. Ka ikdwar ni okan rembi, pod inyalo bedo e nonro. Pod ibiro pimi ka in gi malaria kendo ka in gi remo ma oromo. Ka in kata nyathini oyud gi malaria to pod ibiro thiedhu.

Tudruok ka in gi penjo kata wach machandi

Ka in gi penjo mora mora e wi nonro ni to inyalo tudori gi Dr Eric Ochomo mane ofis Kemri/CDC P.O. Box 1578 40100 Kisumu, kata e namba simu 057-2022902. Bende inyalo tudori gi jogwa manie nonro mani kar thieth machiegni kodi. Ka in gi penjo kuom ratiro mari ka ngama nie nonro kata kadidwar loso gi ngama onge e nonro nito inyalo tudori gi jagoro mar ratiro mar Kemri e P.O. Box 54840-00200, Nairobi, simu 020 2722541, 0722205901 kata 0733400003. Email seru@kemri.org. Bende inyalo tudori gi jagoro kata jakom mar kamiti mar ratiro ka po ni ihinyori niwach donjo e nonro ni. Ka ionge simu, kata ka ikia ndiko gi somo ok bi moni bedo e nonro ni. Inyalo kwayo namba kar thieth mani machiegni kodi mondo otudi gi jok ma ochiw nyinge gi e oboke ni mondo ilos kodgi e weche machandi kuom nonro ni.

Ka ituo , gochne Dr. Eric Ochomo ma tiyo gi kar timo nonro ma KEMRI/CDC e namba mar simu 057 20 22902 to tem matek mondo **dhi kar thieth mani machiegni kodi**. Erokamano ahinya kuom thuolo ma ichiwo nwa. Ka iyie bedo e nonro to ibiro keto sei e oboke ni.

16.7.14. Consent Statement: Main trial (DhoLuo)

NONRO MAR YUDO PIM MAR YATH IVERMECTIN (IVERMAL)

Weche mane obokemar chiwo ayie

Wi nonro: Nonro mar yudo pim mar yath ma iluongo ni Ivermectin manyalo geng'o landruok mar tuo mar malaria ia

Keto lweti pinyka en ranyisi ni iyie bet enonoro/ ni nyathini obed jachiwre e nonro:

Weche duto manie oboke ni osenyisa ma ayie donjo e nonro ni. Ang'eyo ni an thuolo mar bedo kata wuok e nonro ma onge gima biro tima. Ayie ni remba opim ne tuo mar malaria kod ka bende an gi remo ma oromo. Ange'yo ni remba bende inyalo or e lab mar CDC mani Atlanta kata LSTM Liverpool kar nonro motudore ne nonro ma moko ma ok nyal tim e Kenya ka. Ange'yo ni ripode ma iloso inyalo nen gi jotim nonro ma Kemri/CDC. Amiyo gi thuolo mar ng'iyo weche ga mek nonro. Kendo achiwo thuolo mondo ripode obi ti godo ka osegol nyinge, ka achiel gi adres mara gi nonro moko.	Ka iyie to ndik ni iyie
	YIE

	Nying:	Sei:	Tarik makawuono
Ng'ama duong ma oyie bedo e nonro:		Sei	
		Ranyisi mar lith lwedo maduong	
Janeno*			
Jatim nonro ma chiwo oboke ni			

*Jachiwre kata janyuol nyalo keto seyi gi janeno,kata wach yie mare ka janeno ma bang'e biro keto seyi nitie.

NONRO MAR YUDO PIM MAR YATH IVERMECTIN (IVERMAL)

Yie mar kano remo ni nonro mar kinde mabiro

Wi nonro: Nonro mar yudo pim mar yath ma iluongo ni Ivermectin manyalo geng'o landruok mar tuo mar malaria

Keto lweti piny ka nyiso ni iyie chiwori /nyathini obed jachiwre e nonro.

<table>
<tr><td rowspan="2">Kaka ikano remo osepimna ma ayie ni okan remba kuom higni ma ok tin ne 15 ne nonro ma moko mabiro. Bende ang'eyo ni remba inyalo ter e lab manitie loka CDC Atlanta kata LSTM Liverpool kar nonro motudore timo nonro ma ok nyal tim e Kenya ka. Anyalo loko pacha mondo kik okan remba kata oor loka ne nonro ma moko mabiro. Mondo ma otimre, anyalo nyiso Dr. Meghna Desai ma biro nyiso jononro moko mani Kemri/CDC.</td><td colspan="2">Ka iyie to ket sei kar yie, to ka idagi ket sei kar ooyo</td></tr>
<tr><td>YIE</td><td>OOYO</td></tr>
</table>

	Nying:	Sei:	Tarik ma kawuono
Ng'ama duong ma oyie bedo e nonro:		Sei	
		Ranyisi mar lith lwedo maduong	
Janeno*			
Jatim nonro ma chiwo oboke ni			

*Jachiwre kata janyuol nyalo keto seyi gi janeno,kata wach yie mare ka janeno ma bang'e biro keto seyi nitie.

16.7.15. Informed Consent Participant Information Sheet: Rich PK study (Dholuo)

NONRO MAR YUDO PIM MAR YATH IVERMECTIN (IVERMAL)

Oboke ma otingo weche jachiwre e nonro

Nonro mar neno kaka yadh ni tiyo e dend dhano (Pharmacokinetic study)

Wi nonro: Nonro mar yudo pim mar yath ma iluongo ni Ivermectin manyalo geng'o landruok mar tuo mar malaria

Investigators

Dr Menno Smit, MD, MPH (KEMRI, LSTM)
Dr Eric Ochomo, PhD (KEMRI/CDC)
Dr Titus Kwambai, MD, MSc (MOH, KEMRI/CDC, LSTM) [TK]
Dr Simon Kariuki, PhD (KEMRI/CDC) [SK]
Dr Nabie Bayoh, PhD (KEMRI/CDC) [NB]
Dr Meghna Desai, PhD, MPH (KEMRI/CDC, CDC-Kenya) [MD]
Dr John Gimnig, PhD (CDC) [JG]
Dr Penelope Phillips-Howard, PhD (KEMRI, LSTM) [PPH]
Dr Aaron Samuels, MD, MHS (CDC) [AS]
Mr Ben Abong'o, MS (KEMRI/CDC)
Institutions

KEMRI- Kenya Medical Research Institute and CDC, Kisumu, Kenya
CDC- Centers for Disease Control and Prevention, Atlanta, Georgia, USA
MOH- Ministry of Health, Kenya
LSTM- Liverpool School of Tropical Medicine

Gima miyo watimo nonro

Kar timo nonro mar Kemri ka oriwore gi CDC kod Liverpool School of Tropical Medicine timo nonro. Ka omedore gi nonro mano maduong' ma oyang e oboke machielo mar chiwruok kuom nonro, watimo nonro machielo ma oriwore gi nonro mokwongo mar mondo wayud ng'eyo ndalo ma yath mar ivermectin tieko e remo. Mae idendo gi dho ngere ni pharmacokinetic (PK) study. Wabiro kwayo kanyakla mar ji 36 kuom ji 141 ma niye nonro maduong' mondo odonj e nonro ni.

Kaka wabiro timo:

Ka iyie donjo e nonro machielo ni, wabiro rwaki e ward mar osiptal kuom ndalo adek. Kuom ndalo adek gi, wabiro kawo remo matin maromo ojiko achiel mar chai dibich odiochieng'. Mae wabiro timo ka wachuoyi gi sindan matin mar plastic (ne picha). Sindan maro mar plastic ma wachuoyi biro dong' e badi kuom ndalo adek. Mae nyiso ni ok wabichuoyo badi sandan kendo kuom ndalo adek gi. Bang'

ndalo adek e osiptal wabiro yie ni mondo idog dala. Kata kamano, wabiro kwayo mondo iduog ndalo mathoth kuom juma achiel mokuongo (chieng mar 4, 5, 7, 8, 10) bang'e no kendo dichiel e juma kuom jumbe moko adek (chieng' mar 14, 21 gi 28). Mae en kanyakla mar limbe aboro bang' wuok e osiptal. Samoro amora ma iduogo, wabiro kawo remo matin (madiro ojiko achiel mar chai) e lweti, kendo di 4 bende wabiro kawo remo e lith lweti. Limbe ka limbe biro kawo kaka thuolo manyalo romo dakika 30.

Wabiro keto rembi no e 'tube' matin kendo wabiro timo nonro kuome e kambi marwa ma KEMRI. Bang mano wabiro oro remo moko e piny mar England / America kama remo gi ibiro pim mar ng'iyo pim mar yath mar Ivermectin ma nie rembi. Ka achiel gi mano, ibiro non gigo ma nie dend ji ma opogore ma miyo dend gi tiyo gi yath eyore ma opogore.

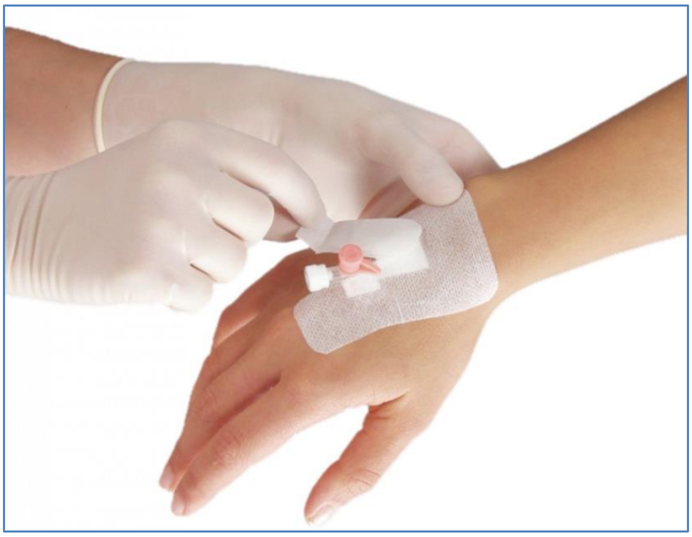

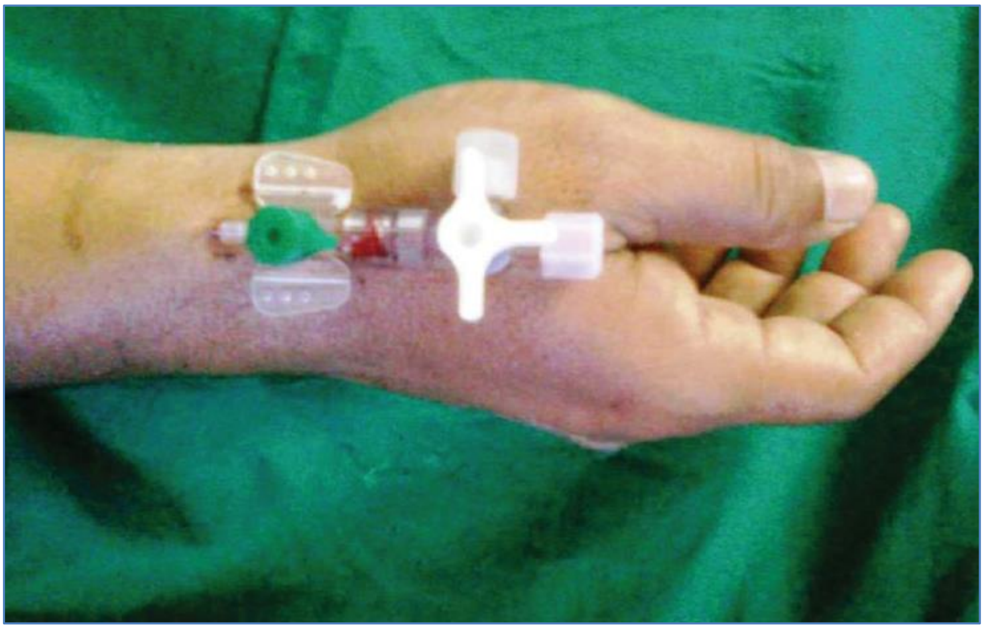

Ber manitie
Onge yuto ma in ibiro yudo ko a kuom nonro ni. Kata kamano, bedo ni e nonro ni biro konyo ng'eyo ma wan godo e 'science' mar tuo mar malaria.

Rach manitie

Remo duto ma wabiro kao kuomi tin ma ok nyal kelo rach moro amora, kata kamano, kama sandam mar mar plastic ochuoyo nyalo bedo malit sama wachuoyi kendo nyalo bedo gi ilo. Bende sama iduogo e osiptal inyalo winjo rem matin sama wagolo remo kendo kama wachuoyo nyalo gwarore matin. Samoro bende kama wachuoyo gi sandan mano mar plastic nyalo bedo gi adhola, kata kamano wabiro riti maber mar mono gima kamano timore. Ka obedo gi adhola, wabiro golo sandan no kendo wabiro thiedhe. Ka iyie to wabiro loko waket sandan e badi machielo.

Ang'o kendo ma wadwaro kuomi?

Nonro machielo ni biro dwaro mondo iduog e osiptal ding'eny. Duogo ni e osiptal e saa kendo odiochieng' ma ochiki ema biro miyo nonro ni otimre maber kendo wayud duoko ma owinjore.

Costs and compensation for being in the study

Ok bi kwayi mondo ichul gimoro amora kuom bedo e nonro ni. Wabiro miyi siling 500 kuom odiochieng' ka odiochieng' ma warwaki e ward. Wabiro miyi chiemo kuom ndalo ma in e osiptal. Wabiro dwoko ni pesa ma itiyo go mondo ichop e osiptal kuom ndalo 4, 5, 7, 8, 10, 14, 21, 28. Pesa ni biro luore gi chik mano mar KEMRI/CDC. Kuom saa ni en siling 300-500 odiochieng' ka odiochieng' ma ibiro e osiptal.

Kano weche ma Opondo

Weche ibiro kan ma Opondo kaka chik owacho. Ripode ma wabiro ndiko biro kan maber e ofi Kemri/CDC e kabade ma iloro kendo e kompyuta ma onge ngata ngata ma okowinjire neon mabiro neno. Weche mabiro wuok e nonro ibiro ti godo mana e nonro ni kendo nyinge u ok bi wuoke ripode moka moka ma ibiro gol. Bang nonro to nyinge duto ibiro gol maonge ngato mabiro ngeyo weche gi kata rembi.

Thuolo kod ratiro mari mar bedo e nonro kata wuok

In thuolo mar bedo e nonro ni. Kendo en ratiro mari tamori ka ok idwar. Bende kata ka isedonjo e nonro to idwaro wuok, in huolo. Ka idagi bende pod en ratiro mari yudo thieth mar malaria ma ibiro chiw.

Tudruok ka in gi penjo kata wach machandi

Ka in gi penjo mora mora e wi nonro ni to inyalo tudori gi Dr Eric Ochomo mane ofis Kemri/CDC P.O. Box 1578 40100 Kisumu, kata e namba simu 057-2022902. Bende inyalo tudori gi jogwa manie nonro mani kar thieth machiegni kodi. Ka in gi penjo kuom ratiro mari ka ngama nie nonro kata kadidwar loso gi ngama onge e nonro nito inyalo tudori gi jagoro mar ratiro mar Kemri e P.O. Box 54840-00200, Nairobi, simu 020 2722541, 0722205901 kata 0733400003. Email seru@kemri.org. Bende inyalo tudori gi jagoro kata jakom mar kamiti mar ratiro ka po ni ihinyori niwach donjo e nonro ni. Ka ionge simu, kata ka ikia ndiko gi somo ok bi moni bedo e nonro ni. Inyalo kwayo namba kar thieth mani machiegni kodi mondo otudi gi jok ma ochiw nyinge gi e oboke ni mondo ilos kodgi e weche machandi kuom nonro ni.

Ka ituo , gochne Dr. Eric Ochomo ma tiyo gi kar timo nonro ma KEMRI/CDC e namba mar simu 057 20 22902 to tem matek mondo dhi kar thieth mani machiegni kodi. Erokamano ahinya kuom thuolo ma ichiwo nwa. Ka iyie bedo e nonro to ibiro keto sei e oboke ni.

16.7.16. Consent Statement: Rich PK study (Dholuo)

NONRO MAR YUDO PIM MAR YATH IVERMECTIN (IVERMAL)

Chiwo AYIE mar bedo e nonro mar PK

Wi nonro: Nonro mar yudo pim mar yath ma iluongo ni Ivermectin manyalo geng'o landruok mar tuo mar malaria ia

Keto lweti pinyka en ranyisi ni iyie bet enonoro/ ni nyathini obed jachiwre e nonro:

Oselerna weche man e obokeni kendo ayie mondo adonji e nonro mar PK. Awinjo maler ni an thuolo mar yiero donjo e nonroni, kendo ni onge rach moro mabiro timorena kata ka adagi. Awinjo maler ni nonro mar PK dwaro ni mondo abedi e od thieth kuom ndalo adek mokwongo . Awinjo maler ni remo matin ibiro gol e bada nya dibich pile pile kuom ndalo adekgo. Awinjo maler ni ibiro soyo sindan mar plastic e bada kuom thuolo mar ndalo adekgo, kendo anyalo neno pek matin kaluwore kod mano. Bende awinjo ni ibiro dwara mondo aduogi e kar thieth e ndalo mar 4, 5, 7, 8, 10, 14, 21, 28. Awinjo maler ni oton mar remo matin ibiro gol kuoma e limbegi.<br><br>Awinjo maler kendo ayie ni moko kuom remo ma ogol kuoma inyalo ter e ute pimo tuoche man CDC Atlanta kata skul mar LSTM Liverpool kar nonro motudore, kata e ute pim mag jogo ma omakore gi nonroni mondo opim yath man e remo to gi kaka denda golo oko yath. Bende achiwo thuolo mondo weche ma ochoki e nonroni ogol ayanga, kata omi jo nonro mamoko, to mana ka osegolie nyinga gi weche momakore gi kaka inyal yuda.	Ka iyie to ndik ni iyie<br><br>YIE

	Nying:	Sei:	Tarik makawuono
Ng'ama duong ma oyie bedo e nonro:		Sei	
		Ranyisi mar lith	
Janeno*			
Jatim nonro ma chiwo oboke ni			

*Jachiwre kata janyuol nyalo keto seyi gi janeno,kata wach yie mare ka janeno ma bang'e biro keto seyi nitie.

16.7.17. Informed Consent Participant Information Sheet: Direct feeding (Dholuo)

NONRO MAR YUDO PIM MAR YATH IVERMECTIN (IVERMAL)

Oboke ma otingo weche jachiwre e nonro

Wi nonro: Nonro mar yudo pim mar yath ma iluongo ni Ivermectin manyalo geng'o landruok mar tuo mar malaria

Jo ta nonro:

Dr Menno Smit, MD, MPH (KEMRI, LSTM)
Dr Eric Ochomo, PhD (KEMRI/CDC)
Dr Titus Kwambai, MD, MSc (MOH, KEMRI/CDC, LSTM) [TK]
Dr Simon Kariuki, PhD (KEMRI/CDC) [SK]
Dr Nabie Bayoh, PhD (KEMRI/CDC) [NB]
Dr Meghna Desai, PhD, MPH (KEMRI/CDC, CDC-Kenya) [MD]
Dr John Gimnig, PhD (CDC) [JG]
Dr Penelope Phillips-Howard, PhD (KEMRI, LSTM) [PPH]
Dr Aaron Samuels, MD, MHS (CDC) [AS]
Mr Ben Abong'o, MS (KEMRI/CDC)
Kuonde ma gitiye:

KEMRI- Kenya Medical Research Institute and CDC, Kisumu, Kenya
CDC- Centers for Disease Control and Prevention, Atlanta, Georgia, USA
MOH- Ministry of Health, Kenya
LSTM- Liverpool School of Tropical Medicine

Gima miyo watimo nonro

Kar timo nonro mar Kemri ka oriwore gi CDC kod Liverpool School of Tropical Medicine timo nonro. Ka achiel gi nonro mane oseler e otas machielo mar chiwo ayie mari, wan gi dwar mar ng'eyo ka suna ma dhodho remo e badi yudo pim maduong' moloyo mar yath moloyo mago ma wamiyo remo ma wagolo kuomi ka watiyo gi sandan. Mae en nikech waparo ni pim mar yadh ni ng'eny e pien ka achiel gi rembi moloyo rembi kende.

Kaka wabiro timo:

Chieng' mar 7 bang' chako muonyo yath, wabiro kwayi mondo iyar badi ei rageng ma okane suna kama suna 50 biro dhodho remo kuomi kuom dakika ma ok ng'eny ne 20. Sama gikayi ibiro winjo ka chandi matin. Suna gi opidh e kar timo nonro marwa ma KEMRi omiyo gi onge gi kudni moro amora ma nyalo kelo tuo. Suna gi madho remo matin kabisa kendo kuom suna gi kanyakla ok bi madho remo mapondo kata ojiko matin mar chai.

Ber manitie
Onge yuto ma in ibiro yudo ko a kuom nonro ni. Kata kamano, bedo ni e nonro ni biro konyo ng'eyo ma wan godo e 'science' mar tuo mar malaria.

Rach manitie
Kama suna okayo nyalo ili kuom ndalo matin, kata kamano, ilo no biro rumo bang' ndalo matin.

Kano weche ma Opondo
Weche ibiro kan ma Opondo kaka chik owacho. Ripode ma wabiro ndiko biro kan maber e ofi Kemri/CDC e kabade ma iloro kendo e kompyuta ma onge ngata ngata ma okowinjire neon mabiro neno. Weche mabiro wuok e nonro ibiro ti godo mana e nonro ni kendo nyinge u ok bi wuoke ripode moka moka ma ibiro gol. Bang nonro to nyinge duto ibiro gol maonge ngato mabiro ngeyo weche gi kata rembi.

Thuolo kod ratiro mari mar bedo e nonro kata wuok
In thuolo mar bedo e nonro ni. Kendo en ratiro mari tamori ka ok idwar. Bende kata ka isedonjo e nonro to idwaro wuok, in huolo.Ka idagi bende pod en ratiro mari yudo thieth mar malaria ma ibiro chiw.

Chudo kata mich e nonro
Ok bi kwayi ni ichul pesa mora mora mondo eka idonj e nonro ni. Ka itiyo gi pesa ni dhi kar thieth e ndalo ma wawinjore to ibiro duok ni pesa no kaka owinjore gi Kemri/CDC. Pesa kind 300-500 e ndalo ma idhi kar thieth.

Thuolo mar kano remo ma okaw kuom ndalo mang'eny ne wach nonro ma moko manyalo bedo:
Wakwayo ji ma odonjo e nonro ka bende wanyalo kano remo ma gi chiwo ne nonro ma moko e ndalo mabiro. Nonro ma moko gi gin mek konyo kaka wanyalo geng'o malaria. Ka iyie, to wabiro kano remo no gi namba ma opogore gi nyingi ma ok wabi tiyo gi nyingi. Kendo ibiro kane kama diny. Ripode ma wayudo kuom remo wanyalo ng'iso jo ma moko ma watudore go mak mana ni ok wabitiyo gi nyingi kata adres mari kata gima nyalo fulo ni en in. Ka nonro oserumo, to wabiro golo gik moko te manyalo nyiso ni en in mane nie nonro. Ka ikdwar ni okan rembi, pod inyalo bedo e nonro. Pod ibiro pimi ka in gi malaria kendo ka in gi remo ma oromo. Ka in kata nyathini oyud gi malaria to pod ibiro thiedhu.

Tudruok ka in gi penjo kata wach machandi
Ka in gi penjo mora mora e wi nonro ni to inyalo tudori gi Dr Eric Ochomo mane ofis Kemri/CDC P.O. Box 1578 40100 Kisumu, kata e namba simu 057-2022902. Bende inyalo tudori gi jogwa manie nonro mani kar thieth machiegni kodi. Ka in gi penjo kuom ratiro mari ka ngama nie nonro kata kadidwar loso gi ngama onge e nonro nito inyalo tudori gi jagoro mar ratiro mar Kemri e P.O. Box 54840-00200, Nairobi, simu 020 2722541, 0722205901 kata 0733400003. Email seru@kemri.org. Bende inyalo tudori gi jagoro kata jakom mar kamiti mar ratiro ka po ni ihinyori niwach donjo e nonro ni. Ka ionge simu, kata ka ikia ndiko gi somo ok bi moni bedo e nonro ni. Inyalo kwayo namba kar thieth mani machiegni kodi mondo otudi gi jok ma ochiw nyinge gi e oboke ni mondo ilos kodgi e weche machandi kuom nonro ni.

Ka ituo, gochne Dr. Eric Ochomo ma tiyo gi kar timo nonro ma KEMRI/CDC e namba mar simu 057 20 22902 to tem matek mondo, dhi kar thieth mani machiegni kodi. Erokamano ahinya kuom thuolo ma ichiwo nwa. Ka iyie bedo e nonro to ibiro keto sei e oboke ni.

16.7.18. Consent Statement: Direct feeding (DhoLuo)

NONRO MAR YUDO PIM MAR YATH IVERMECTIN (IVERMAL)

Weche mane obokemar chiwo ayie

Wi nonro: Nonro mar yudo pim mar yath ma iluongo ni Ivermectin manyalo geng'o landruok mar tuo mar malaria ia

Keto lweti pinyka en ranyisi ni iyie bet enonoro/ ni nyathini obed jachiwre e nonro:

<table>
<tr><td rowspan="2">Weche duto manie oboke ni osenyisa ma ayie donjo e nonro ni.Ang’eyo ni an thuolo mar bedo kata wuok e nonro ma onge gima biro tima. Ayie ni remba opim ne tuo mar malaria kod ka bende an gi remo ma oromo. Ange’yo ni remba bende inyalo or e lab mar CDC mani Atlanta kata LSTM Liverpool kar nonro motudore ne nonro ma moko ma ok nyal tim e Kenya ka. Ange’yo ni ripode ma iloso inyalo nen gi jotim nonro ma Kemri/CDC. Amiyo gi thuolo mar ng’iyo weche ga mek nonro. Kendo achiwo thuolo mondo ripode obi ti godo ka osegol nyinge, ka achiel gi adres mara gi nonro moko.</td><td>Ka iyie to ndik ni iyie</td></tr>
<tr><td>YIE</td></tr>
</table>

<table>
<tr><td></td><td>Nying:</td><td>Sei:</td><td>Tarik makawuono</td></tr>
<tr><td rowspan="2">Ng’ama duong ma oyie bedo e nonro:</td><td></td><td>Sei</td><td></td></tr>
<tr><td></td><td>Ranyisi mar lith lwedo maduong</td><td></td></tr>
<tr><td>Janeno*</td><td></td><td></td><td></td></tr>
<tr><td>Jatim nonro ma chiwo oboke ni</td><td></td><td></td><td></td></tr>
</table>

*Jachiwre kata janyuol nyalo keto seyi gi janeno,kata wach yie mare ka janeno ma bang’e biro keto seyi nitie.

16.8. APPENDIX VIII. QUESTIONNAIRES

To be designed
