# Supplementary material for: Efficacy and Safety of High-Dose Ivermectin for Reducing Malaria Transmission (IVERMAL): Protocol for a Double-Blind, Randomized, Placebo-Controlled, Dose-Finding Trial in Western Kenya
Source: JMIR Res Protoc. 2016 Nov 17;5(4):e213. doi: 10.2196/resprot.6617 (PMC5133431; doi:10.2196/resprot.6617)
Supplement: Multimedia Appendix 3 [file resprot_v5i4e213_app3.pdf]

EFFICACY AND SAFETY OF HIGH-DOSE IVERMECTIN FOR REDUCING MALARIA TRANSMISSION: A DOSE FINDING STUDY

STATISTICAL ANALYSIS PLAN

Registration number Clinicaltrials.gov: NCT02511353

Chief Investigator: Prof. Feiko ter Kuile

Trial statisticians: Prof. Duolao Wang

SAP authors: Dr. Menno Smit, Dr. Eric Ochomo, Prof. Feiko ter Kuile, Prof. Duolao Wang

**SAP version history**		
Version Date	SAP Version #	Details of Changes
19 Feb, 2016	*1.0*	Original

**Signatures**		
	Signature	Date
Prof. Duolao Wang (Trial Statistician)		
Prof. Feiko ter Kuile (Chief Investigator)		
Dr. Timothy Collier (Statistician: DMEC)		

TABLE OF CONTENTS

PAGE

ABBREVIATIONS 5

1. INTRODUCTION 7

2. STUDY OBJECTIVES AND OUTCOMES 7
    2.1. Study objectives 7
        2.1.1. Primary objective 7
        2.1.2. Secondary objectives 7
    2.2. Outcomes 7
        2.2.1. Primary outcome 7
        2.2.2. Secondary outcomes 7
        2.2.3. Safety outcomes 9
        2.2.4. Morbidity outcomes 9
        2.2.5. Tolerability outcomes 10
    2.3. Case ascertainment and case definitions 10
        2.3.1. Malaria infection case definitions 10
        2.3.2. Entomological case definitions 11

3. STUDY DESIGN 11
    3.1. Design 11
    3.2. Trial sites 12
    3.3. Treatments 12
    3.4. Randomization 12
    3.5. Sample size 13

4. ANALYSIS POPULATIONS 14
    4.1. Study population data sets 14
    4.2. Analysis close date 14
    4.3. Data download 14

5. STATISTICAL ANALYSES 15
    5.1. Primary outcome analysis 15
        5.1.1. ITT analysis of the primary outcome - the primary analysis 15
        5.1.2. Per-protocol analysis of the primary outcome 15
        5.1.3. Covariate adjusted analysis of the primary outcome 15
        5.1.4. Subgroup analysis of the primary outcome 15
    5.2. Secondary outcome analysis 16
        5.2.1. Analysis of binary outcomes 16
        5.2.2. Analysis of time-to-event outcomes 16
        5.2.3. Analysis of continuous outcomes 16
        5.2.4. Analysis of count outcomes 16
        5.2.5. Analysis of secondary outcomes with repeated measurements 16
        5.2.6. Analysis of other secondary outcomes 17
    5.3. Safety analyses 17
    5.4. Exploratory analyses 17

6. GENERAL CONSIDERATIONS FOR DATA ANALYSES 17
6.1. Multi-site consideration 17
6.2. Covariates analyses 17
6.3. Subgroup analysis 18
6.4. Multiplicity 18
6.5. Missing data 18
6.6. Other data considerations 18
6.6.1. Data summaries 18
6.6.2. Graphical displays 18

7. REFERENCES 18

8. LIST OF SUMMARY TABLES AND FIGURES 19

ABBREVIATIONS

Abbreviation	Explanation
95% CI	95 percent Confidence Interval
ACT	Artemisinin-based combination therapy
AE	Adverse event
AIDS	Acquired Immunodeficiency Syndrome
AL	Artemether-Lumefantrine
AUC	Area Under the Curve
CDC	Centers for Disease Control and Prevention
CHW	Community Health Worker
CHEW	Community Health Extension Worker
Cmax	Maximum drug concentration
CRF	Case Record Form
CRO	Contract Research Organization
DHA	Dihydroartemisinin
DP	Dihydroartemisinin-piperaquine
DSMB	Data Safety Monitoring Board
DMEC	Data Monitoring and Ethics Committee
ERC	Ethics Research Committee
FDA	Food and Drug Administration
GCP	Good Clinical Practice
GMP	Good Manufacturer Practice
Hb	Haemoglobin
HIV	Human Immunodeficiency Virus
IRB	Institutional Review Board
ITNs	Insecticide Treated Nets
ITT	Intention to Treat
IVM	Ivermectin
KEMRI	Kenya Medical Research Institute
LLINS	Long-lasting Insecticide Treated Nets
LSTM	Liverpool School of Tropical Medicine
MDA	Mass drug administration
MIC	Minimum Inhibitory/Insecticidal Concentration
MoH	Ministry of Health
MPAC	Malaria Policy Advisory Committee
MRC	Medical Research Council, UK
NMCP	National Malaria Control Program
PCR	Polymerase Chain Reaction
PK	Pharmacokinetic
PQ	Primaquine
RCT	Randomized Controlled Trial
RDT	Rapid diagnostic test
REC	Research Ethics Committee
SAE	Serious adverse event
SOP	Standard Operating Procedure
T1/2	plasma half-life

Abbreviation	Explanation
QTc	QT corrected time interval between Q and T on electrocardiogram
QTcF	QTc using Fridericia’s correction ($QTcF = QT/RR^{0.33}$)
tCTU	Tropical Clinical Trials Unit
Tmax	time to maximum plasma concentration
TSC	Trial Steering Committee
WHO	World Health Organization

1. INTRODUCTION

The purpose of this Statistical Analysis Plan (SAP) is to define the outcome variables, statistical methods, and analysis strategies to address the study's objectives in the double blind, randomised, placebo controlled trial assessing the "Efficacy and safety of high-dose ivermectin for reducing malaria transmission: A dose finding study (IVERMAL study)".

2. STUDY OBJECTIVES AND OUTCOMES

2.1. Study objectives

2.1.1. Primary objective

To determine the safety and efficacy of ivermectin 0, 300, 600 mcg/kg/day for 3 days, when provided with a standard 3-day course of dihydroartemisinin-piperaquine (DP) for uncomplicated malaria, on 14-day mosquito survival after feeding experiment performed at 7 days after treatment (i.e. 5 days after the last dose of ivermectin).

2.1.2. Secondary objectives

1. To determine the effect of different doses of ivermectin on oocyst and sporozoite rates.
2. To determine the effect of different doses of ivermectin on other ectoparasites (e.g. head lice, scabies).
3. To determine the pharmacokinetic profile of the different ivermectin regimens
4. To determine if ivermectin interacts with the pharmacokinetics of piperaquine
5. To determine whether the addition of ivermectin to DP interacts with the ability of DP to clear asexual and sexual stage parasites
6. To determine the role of genetic variants of CYP3A4 activity in metabolizing ivermectin
7. To determine the effect of direct feeding versus membrane feeding on mosquito survival

2.2. Outcomes

2.2.1. Primary outcome

2.2.1.1. Mosquito death by day 14: Occurrence of mosquito death by day 14 after the feeding experiment performed at 7 days after start of treatment.

2.2.2. Secondary outcomes

2.2.2.1. Time to mosquito death from each feeding experiment (performed at 0, 2 days+4h, 7, 10, 14, 21, 28 days after start of treatment) up to 28 days post-feeding.

2.2.2.2. Mosquito death by day 10: Occurrence of mosquito death by day 10 after the feeding experiment performed at 7 days after start of treatment.

2.2.2.3. Oocyst prevalence: mosquitoes surviving on the day of oocyst PCR will be divided into 2 groups, and for each group a PCR will be run. Oocyst prevalence will

be defined by the number of batches per feed that were positive by PCR (0, 1, or 2 batches).

2.2.2.4. The risk of persistent parasiteamia by day 2 (yes/no).

2.2.2.5. The cumulative risk of recurrence (recrudescence or reinfection) of malaria infection, between day 4 and Day 28.

2.2.2.6. The cumulative risk of reinfection of malaria infection (differentiated from recrudescence by genotyping), between day 4 and Day 28.

2.2.2.7. The cumulative risk of recrudescence of malaria infection (differentiated from reinfections by genotyping), between day 4 and Day 28.

2.2.2.8. Malaria clinical and parasitological treatment response by day 28 (value: 1, 2, 3, or 4; WHO 2005):

1. Early treatment failure:
    - on day 1, 2 or 3 in the presence of parasitaemia: danger signs or severe malaria;
    - on day 2: parasitaemia >100% of day 0;
    - on day 3: parasitaemia ≥ 25% of day 0;
    - on day 3 in the presence of parasitaemia: axillary temperature ≥ 37.5 ºC.
2. Late clinical failure: presence of parasitaemia on any day between day 4 and day 28 in patients who did not previously meet any of the criteria of early treatment failure, in combination with either:
    - danger signs or severe malaria, or
    - axillary temperature ≥ 37.5 ºC (or history of fever)
3. Late parasitological failure: presence of parasitaemia on any day between day 4 and day 28 in patients who did not previously meet any of the above criteria (early treatment failure, or late clinical failure)
4. Adequate clinical and parasitological response: absence of parasitaemia on day 28, in patients who did not previously meet any of the criteria of early treatment failure, late clinical failure or late parasitological failure.

2.2.2.9. Plasma concentration profiles of piperaquine and ivermectin as described by standard pharmacokinetic metrics (e.g. $AUC_{0-\infty}$, $AUC_{0-tlast}$, C_{max}, $t_{½}$, t_{max}, etc).

2.2.3. Safety outcomes

2.2.3.1. Mydriasis: Change in Pupil diameter (mm) at Day 2+4h from:

2.2.3.1.1. *Day 0 (baseline)*

2.2.3.1.2. *Day 28 (alternative baseline)*

2.2.3.2. QT-interval: Change in QTcF (ms) at Day 2+4h from:

2.2.3.2.1. *Day 0 (baseline)*

2.2.3.2.2. *Day 28 (alternative baseline)*

2.2.3.3. QTcF-prolongation: Occurrence of QTcF-prolongation on Day 2+4h:

2.2.3.3.1. *≥ 450 ms*

2.2.3.3.2. *≥ 480 ms*

2.2.3.3.3. *≥ 500 ms*

2.2.3.3.4. *≥30 ms increase from Day 0 (baseline)*

2.2.3.3.5. *≥60 ms increase from Day 0 (baseline)*

2.2.3.3.6. *≥30 ms increase from Day 28 (alternative baseline)*

2.2.3.3.7. *≥60 ms increase from Day 28 (alternative baseline)*

2.2.3.4. Number of SAEs during follow-up period:

2.2.3.4.1. *Overall*

2.2.3.4.2. *by system organ class and preferred term*

2.2.4. Morbidity outcomes

2.2.4.1. Death, (yes/no): The death of a participant during the 28-day enrolment period, or thereafter as a result of a primary event during the 28-day period.

2.2.4.2. Haemoglobin and prevalence of anaemia during follow-up:

2.2.4.2.1. *Haemoglobin drop (g/dL): change between baseline and lowest point*

2.2.4.2.2. *Any anaemia: Hb<13.0 g/dL in men, Hb<12.0 g/dL in (non-pregnant) women (yes/no)*

2.2.4.2.3. *Moderate anaemia: Hb<9,0 g/dL (yes/no)*

2.2.4.2.4. *Severe anaemia: Hb<7,0 g/dL (yes/no)*

2.2.5. Tolerability outcomes

2.2.5.1. Number of non-serious AEs during follow-up period:

2.2.5.1.1. *Overall*

2.2.5.1.2. *by system organ class and preferred term*

2.2.5.2. Treatment:

2.2.5.2.1. *Treatment Compliance: all 3 daily doses of DP+IVM taken within 6 hours of scheduled time (yes/no)*

2.2.5.2.2. *Dose intolerance (DP+IVM) on Day-1 (%): Vomited DP+IVM on day-1 within 30 minutes of administration.*

2.2.5.2.3. *Dose intolerance (DP+IVM) on Day-2 (%): Vomited DP+IVM on day-2 within 30 minutes of administration.*

2.2.5.2.4. *Dose intolerance (DP+IVM) on Day-3 (%): Vomited DP+IVM on day-3 within 30 minutes of administration.*

2.2.5.2.5. *Course intolerance (DP+IVM) (%): dose intolerance (DP+IVM) on Day-1, or Day-2, or Day-3.*

2.3. Case ascertainment and case definitions

2.3.1. Malaria infection case definitions

For all the below definitions of malaria infection reference is made to any species (Plasmodium falciparum, vivax, malariae, ovale, etc), unless otherwise specified (specifically at [pre-screening). Presence of gametocytes only, in the absence of malaria treatment in the last 2 weeks, is considered as malaria infection.

1. (Pre-)Screening: malaria infection, (yes/no): Falciparum malaria infection (with or without other species) detected in the peripheral blood by either:
    a. Rapid microscopy (10% stain)
    b. RDT (HRP2/pLDH Pf-combo), in the absence of malaria treatment in the last 2 weeks
2. Study visits (before Day 14): malaria infection, (yes/no): malaria infection detected during scheduled study visits (Days 0, 2, 7, 10) in the peripheral blood by:
    a. Standard microscopy (3% stain)
    b. In the case of fever and/or history of fever: Rapid microscopy (10% stain)

3. Study visits (from Day 14): malaria infection, (yes/no): malaria infection detected during scheduled study visits (Days 14, 21, 28) in the peripheral blood by either:
    a. Standard microscopy (3% stain)
    b. In the case of fever and/or history of fever, or in the case of a positive routine RDT (pLDH only): Rapid microscopy (10% stain)
4. Unscheduled visits: malaria infection, (yes/no): malaria infection detected during unscheduled visits in the peripheral blood by either:
    a. When microscopy is available: Rapid microscopy (10% stain). From Day 14, RDT (pLDH only) is also performed, participants with negative results can return home pending microscopy results, however all participants are treated based on their microscopy results.
    b. When microscopy is unavailable: a positive RDT (pLDH only)
5. Clinical malaria, (yes/no), both:
    a. Documented fever (>=37.5 °C), or recent history of fever in the last 24 or 48 hours, or other symptoms of acute illness that resulted in a participant seeking care or alerting the study team
    b. Malaria infection
6. Asexual parasite density by microscopy
    a. parasite density using natural log transformed densities, expressed per mm^3, using either white cell count from complete blood counts, the combined volume of the number of High Powered Fields (HPF's) counted based on the microscope ocular's Field Number (FN), or assuming a white blood cell count of $8,000/mm^3$.

2.3.2. Entomological case definitions

1. Insectary condition (tertiles): during mosquito follow-up, the number of events that temperature (27°C ±2°C) or humidity (80% ±10%) was out of range.
2. Mosquito crowding (tertiles): per feed the number of mosquitoes fully fed

3. STUDY DESIGN

3.1. Design

Double-blind placebo-controlled, 3-arm parallel, superiority trial to determine the effect of different doses of ivermectin on mosquito survival. The unit of randomization will be the patient, and the unit of analysis will be the mosquito. The primary endpoint will be the occurrence of mosquito death at 14 days after a feed on blood from a patient who has started ivermectin 7 days earlier (i.e. about 5 days after the last dose of ivermectin with a 3-day regimen administering ivermectin at 0, 24, and 48 hours [days 0, 1 and 2]). Because mosquito feeding involves approximately 100 mosquitoes per feed, the study will use a cluster randomized design with the patient as the cluster unit. The study will have a nested rich pharmacokinetic component in the first 12 patients per study arm, and a sparse sampling population pharmacokinetic component in the remaining patients.

3.2. Trial sites

The study will be conducted in the Provincial Hospital in Kisumu, western Kenya recently renamed to the Jaramogi Oginga Odinga Teaching and Referral Hospital (JOOTRH). Dependent on the recruitment rate, participants will be recruited from multiple sites. However, all participants will be brought to the main trial site for screening, randomization, treatment and follow-up. Therefore, no analysis by site will be done.

3.3. Treatments

Participants will be randomized to one of 3 arms:

1. Dihydroartemisinin-piperaquine (DP) alone, DP plus ivermectin 0 mcg/kg/day for 3 days (“placebo” arm).
2. DP plus ivermectin 300 mcg/kg/day plus 300 mcg/kg/day ivermectin-placebo for 3 days (“300 mcg/kg” arm).
3. DP plus ivermectin 600 mcg/kg/day (“600 mcg/kg” arm).

Patients will receive their weight-based doses of DP and ivermectin/placebo as directly observed therapy in the out-patient clinics or in-hospital if they were admitted for the pharmacokinetic sub-studies.

3.4. Randomization

Four block randomization sequences will be computer-generated by an independent statistician, one for participants in each of the four strata: (1) Female, BMI<23 kg/m², (2) Female, BMI≥23 kg/m², (3) Male, BMI<21 kg/m², and (4) Male, BMI≥21 kg/m². The length of each block will be fixed (n=3) to ensure that should the insectary conditions vary (e.g. temperature & humidity) that there will not be large imbalances between the study arms that could affect mosquito survival (primary outcome). The allocation ratio for the three study arms is 1:1:1. SAS Proc Plan will be used for randomisation code generation.

The study is double-blinded to all participants and study staff. Allocation concealment will be achieved by use of sealed opaque envelopes. All study participants in all 3 arms will receive standard dose DP. Additionally, for the ivermectin component, the placebo group will receive placebo-ivermectin tablets only, the 600 mcg/kg/day received active ivermectin only, and the 300 mcg/kg dose group will receive a combination of active ivermectin and ivermectin placebo tablets, such that each arm will receive the same number of tablets in each weight strata.

Participants will be recruited from multiple sites, however all will be brought to the main trial site for screening, randomization, treatment and follow-up. The randomization sequences will be sent to the study pharmacist in Kenya, who will then prepare the opaque envelopes, numbered sequentially, with the allocated group code and details for the number inside. For each newly enrolled participant, an envelope from the correct sequence (according to stratum) will be opened sequentially to identify the study arm (X, Y, or Z) that they were allocated.

3.5. Sample size

Main Trial

The study is powered for the primary outcome and is based on the following parameters: it requires a total of 141 participants (47 participants in the 0, 300 and 600 mcg/kg/day groups each) and is powered at 80% to detect a relative increase of 30% (RR 1.300) in the 14-day post-feeding mortality rate from 24% in the control group (0 mcg/kg ivermectin) to 31.2% in the 300 mcg/kg/day group, and a 25% (RR1.246) increase from 31.2% with 300 mcg/kg/day to 38.9% in 600 mcg/kg/day recipients, measured from blood taken 7 days after the start of intake of ivermectin and using clusters of 100 anopheline mosquitoes allowing for 10% non-feeders (significance level: 0.05). The same sample size would give 90% power to detect a 35% [RR 1.348] increase from 24% (0 mcg/kg/day) to 32.4% (300 mcg/kg/day), and 27.7% increase [RR 1.285] from 32.4% (300 mcg/kg/day) to 41.3% (600 mcg/kg/day). The calculations assume an intracluster correlation coefficient (ICC) of 0.0622 and allow for 6.5% loss-to follow-up of participants by day 7 (i.e. 44 patients per arm contribute to the primary analysis). The 10% non-feeding rate is based on current data from the same laboratories at KEMRI. The 24% mortality rate estimate by day 14 post-feeding in the control arm is average of observation at KEMRI (18.3%) and in a recent study in Burkina Faso, which showed a 21.2% mortality by day 10, which when extrapolated with 4 additional days predicted a mortality of 29.7% by day 14. The ICC value of 0.0622 is based on the recent study in Burkina Faso that used a coefficient of variation of 0.5 in their sample size calculation and an estimated 20% mortality among mosquitoes fed on volunteers treated with 0 or 200 mcg/kg ivermectin (Bousema, personal communications). Assumptions used for the sample size calculation (e.g. control group mortality, non-feeding rate, ICC, loss-to-follow-up, etc) should be checked during the interim analysis.

Nested direct feeding pilot study

The goal of this sub-study is to determine whether direct feeding results in greater exposure to ivermectin and therefore greater killing effect than membrane feeding. An initial sample of 27 participants (9 per arm) of which 24 (8 per arm) contribute to the analysis was required after which data monitoring and ethic committee was to review the information and approve further recruitment for the remaining patients in the study. Continued recruitment was approved by the DMEC on 13-Nov-2015. The initial sample size of 27 assumed that the study remained blinded and that therefore one third of the 24 patients fall in the placebo arm, and that about 8 are from the 300 and 600 mcg/kg/day arms each. The average killing effect in the 300 and 600 arms is taken as the mean of 25.03 and 32.54 = 28.79%. To show a relative difference of 66.7% (i.e. RR = 1.67) a sample of 8 per arm is required to detect a difference between 28.8% with membrane feeding and 48.1% with direct feeding (1.67 x 28.8%) with 80% power, alpha of 0.05 and ICC of 0.0622 as above. To accommodate for loss to follow-up, refusals or unsuccessful assays, 9 will be recruited per arm.

The current approval rate for direct feeding is approximately 50% (based on recruitment among the first 37 patients). If we assume approximately 17 per arm agree to direct feeding overall, and that 15 of them contribute to the analysis, then the sub-study would have 80% power to detect a 50% increase in mortality from 28.79% to 43.07% (RR=1.496) (ICC=0.0622, two-sided alpha 0.05).

4. ANALYSIS POPULATIONS

4.1. Study population data sets

Three study populations will be considered in the analysis as follows:

Intention-to-treat population

The intention-to-treat population (the full analysis population) is defined as all the mosquitoes who have been fed the blood of patients who have received at least one dose of study medication (dosed subjects) and will be included in the intention-to-treat analysis regardless of whether the participant they received blood from completed the last study visit.

Per-protocol population

The per-protocol population is defined as all the mosquitoes who have been fed the blood of patients who took all 3 daily doses approximately (+/- 6 hours) within the 48 hour timeline, and who completed the primary endpoint visit.

Safety population

All patients who were randomized and received the first dose of study intervention and were followed up at least once; i.e. provided information on potential adverse events.

Pharmacokinetic population

All participants will contribute to the pharmacokinetic population as per the dose received. PK analyses by arm will only include the participants that received 3 doses from the same study arm (as per randomization or per error) within ±6 hours of the scheduled dosing time.

4.2. Analysis close date

The analysis close date is 8-weeks (±3 days) after the last participant has been enrolled. This allows for 4-weeks of participant follow-up (±3 days, as participants have 3 days flexibility in attending the final visit) and 4-weeks of mosquito follow-up from the last visit.

Data cleaning

The data will then be checked to ensure that there are no erroneous entries and that all missing data is properly coded. Any changes will be made on the paper Teleforms and updated in the database accordingly.

4.3. Data download

For each time point, once all data have been inputted and checked, the database will be locked and a data download request made. The data will be downloaded into ACCESS, SAS, SPSS and STATA formats for statistical analyses.

5. STATISTICAL ANALYSES

5.1. Primary outcome analysis

5.1.1. ITT analysis of the primary outcome - the primary analysis

Primary outcome will be summarised by number (%) of events and analysed using a Generalized Estimating Equations (GEE) model with binomial distribution and log link function that includes treatment as a single predictor taking the cluster design into account, which will generate risk ratio (RRs) together with their 95% confidence interval of having a primary endpoint between two active dose groups and the placebo group. Exchangeable covariance structure will be used. The primary endpoint analysis will be based on the ITT population. The main conclusions in the clinical report will be based on the ITT analysis of the primary outcome.

5.1.2. Per-protocol analysis of the primary outcome

A supportive analysis of the primary outcome will also be performed on the per-protocol population. Statistical methods will be the same as used in the Section 5.1.1.

5.1.3. Covariate adjusted analysis of the primary outcome

Covariate adjusted analysis will also be performed on the primary endpoint using the GEE model. The GEE model will include the treatment as the study variable and the following variableas as covariates:

(1) Participant sex [Male or Female].
(2) BMI (< or ≥ median).
(3) Mosquito age at time of feeding [< or ≥ median].
(4) Insectary conditions [tertiles].
(5) Mosquito crowding [tertiles].

From the above model, the adjusted RR and its 95% CIs comparing the **300** arm to the **placebo** arm, the **600** arm to the **placebo** arm, and the **600** arm to the **300** arm will be derived.

The above GEE model with binomial distribution and log link function may not converge when all covariates are introduced into the model simultaneously. If this occurs, the adjusted GEE model will be established by removing a covariate one by one starting from the last covariate in the above list until the model conveges.

5.1.4. Subgroup analysis of the primary outcome

Subgroup analyses of the primary endpoint will also be performed using the GEE model. The following covariates will be used for subgroup analyses:

(1) Participant sex [Male or Female].
(2) BMI (< or ≥ median).

For subgroup analyses, the GEE model will include the treatment as the only study variable and will be performed separately by a subgroup variable as listed above. In addition, assessment of

the homogeneity of treatment effect by a subgroup variable will be conducted by a GEE model with treatment, subgroup variable, and interaction between treatment and subgroup variable as fixed effects, and cluster as cluster effect. *P*-value from interaction term will be presented as evidence of treatment heterogeneity.

5.2. Secondary outcome analysis

All secondary outcomes will be analysed as a superiority design and two-sided 95% CIs for the treatment differences in these outcomes between two treatment groups will be calculated and presented. Secondary outcome analyses will be performed on both the ITT and PP populations.

5.2.1. Analysis of binary outcomes

Binary secondary outcomes will be analysed in a similar way as the primary endpoint.

5.2.2. Analysis of time-to-event outcomes

Time to mosquito death from each feeding experiment (performed at 0, 2 days+4h, 7, 10, 14, 21, and 28 after the start of treatment) will be analysed as time-to-event outcomes (e.g. time to death from feeding to the end of day 28) and will be summarised by number (%) of mosquitoes with event, mosquito-days, and incidence rate by treatment arm.

Kaplan-Meier plots will be drawn to describe the process of death by treatment arms. Cox regression model with shared frailty, to allow for the correlation between mosquito observations from the same feed, will be used to derive hazard ratios, their two-sided 95% confidence intervals, and p-values for comparing the trial arms.

5.2.3. Analysis of continuous outcomes

The continuous outcomes such as participant's hemoglobin will be summarised using number of subjects (n), mean, geometric mean, standard deviation (SD), median, minimum, and maximum by treatment group, and will be analysed by a GEE model with Gauss distribution and identity link function to derive the mean differences and 95% CI. Exchangeable covariance structure will be used.

5.2.4. Analysis of count outcomes

The count outcome such as oocyst prevalence will be summarised using number of events and incidence rate by treatment group, and will be analysed by a GEE model with treatment as fixed effect and with Poisson distribution and log link function. Incidence rate ratio (IRR) comparing two treatments with their two-sided 95% confidence intervals will be derived from the GEE model.

5.2.5. Analysis of secondary outcomes with repeated measurements

The participant's hemoglobin is measured on days 0, 2, 7, 10, 14, 21 and 28. Changes in hemoglobin over 28 days from baseline will be summarised and analysed using a generalised

estimating equation (GEE) model with treatment, day and interaction between treatment and day as fixed effects, baseline measurement of hemoglobin as covariate, and subject as cluster effect. Ratio in geometric mean with their two-sided 95% confidence intervals in fungal count between two arms by day will be derived from the GEE model.

5.2.6. Analysis of other secondary outcomes

Other statistical methods may be used if deemed necessary.

5.3. Safety analyses

Safety, morbidity and tolerability variables will be analysed as secondary outcomes as described above.

Adverse reactions will be reported and tabulated for each treatment arm, overall and per body system on per protocol basis. Treatment emergent adverse events are defined as adverse events that had an onset day on or after the day of the first dose of study medication. Adverse events that have missing onset dates will be considered to be treatment emergent. No formal statistical testing will be undertaken.

5.4. Exploratory analyses

Additional exploratory analyses will be performed to try to understand the potentially large number of unknown confounders/effect modifiers in this study.

6. GENERAL CONSIDERATIONS FOR DATA ANALYSES

SAS® (version 9.4) will be used to perform all data analyses and generate the majority of data displays. STATA or SPSS or S-Plus or R may also be used for some data analyses.

6.1. Multi-site consideration

Participants will be recruited from multiple sites, however all will be brought to the main trial site for screening, randomization, treatment and follow-up. Therefore, no analysis by site will be done.

6.2. Covariates analyses

Covariate analyses will be performed on the primary outcome and the key secondary outcomes, in particular mosquito mortality of the ITT population. Covariates will include participant sex, BMI, mosquito age at time of feeding, insectary conditions and mosquito crowding as defined in Section 5.1.3

6.3. Subgroup analysis

Subgroup analyses will be performed for the primary outcome on the ITT population. The variables for subgroup analysis will include participant sex and BMI as defined in Section 5.1.4.

6.4. Multiplicity

Multiplicity adjustment will not apply to the primary and secondary outcome analyses.

6.5. Missing data

Every effort will be made to minimise the amount of missing data in the trial. Whenever possible, information on the reason for missing data will be obtained. Missing values for covariates at baseline will be imputed using simple imputation method for the covariate adjusted analysis but not for the subgroup analysis. Multiple imputations will be used if the proportion of missing is larger than 5%.

6.6. Other data considerations

6.6.1. Data summaries

Continuous variables will be summarised according to number of subjects with non-missing data (n), mean, standard deviation (SD), median, minimum, and maximum.

Categorical variables will be summarised according to the absolute frequency and percentage of subjects (%) in each category level. The denominator for the percentages is the number of subjects in the treatment arm with data available, unless noted otherwise. Event rates per 100 participant years will also be reported for time-to-event clinical outcomes and adverse events of special interest.

6.6.2. Graphical displays

Mean values for some continuous outcomes by treatment and day will be plotted. Kaplan-Meier plots will be produced for displaying the time-to-event data by treatment and day.

7. REFERENCES

8. LIST OF SUMMARY TABLES AND FIGURES

**Section 1**	**Patient disposition**
Table 1.1	Patient disposition by treatment
Table 1.2	Patient disposition by sex & BMI group and treatment
**Section 2**	**Baseline information**
Table 2.1	Demographic characteristics of patients (ITT)
Table 2.2	Medical history / Prior medication (ITT)
Table 2.3	Physical examination (incl pupillometry and ECG) (ITT)
Table 2.4	Laboratory results (ITT)
**Section 3**	**Medication**
Table 3.1	Drug treatment (ITT)
Table 3.2	Concomitant medication (ITT)
**Section 4**	**Follow-up information** (ITT)
Table 4.1	Follow-up visits (ITT)
Table 4.2	Physical examination (incl pupillometry and ECG) (ITT)
Table 4.3	Laboratory results (ITT)
**Section 6**	**Adverse events (**Safety Population=SP)
Table 6.1.1	Summary of adverse event (SP)
Table 6.1.2	Summary of adverse event by system organ class and preferred term (SP)
Table 6.1.3	Summary of serious adverse event by system organ class and preferred term (SP)
Table 6.1.4	Summary of adverse event by severity, system organ class and preferred term (SP)
Table 6.1.5	Summary of adverse event by causality, system organ class and preferred term(SP)
**Section 7**	**Efficacy**
Table 7.1	Primary outcome analysis (ITT)
Table 7.1a	Primary outcome analysis (PP)
Table 7.2	Secondary efficacy outcomes (ITT)
Table 7.3	Safety outcomes (SP)
Table 7.4	Malaria infection endpoint definitions (ITT)
Table 7.5	Morbidity endpoint definitions (ITT)
Table 7.6	Tolerability endpoint definitions (ITT)
**Section 8**	**Covariate adjusted analysis**
Table 8.1	Covariate adjusted analysis of primary outcome (ITT)
Table 8.1a	Covariate adjusted analysis of primary outcome (PP)
**Section 9**	**Subgroup analysis**
Table 9.1	Subgroup analysis of primary outcome (ITT)
Table 9.1a	Subgroup analysis of primary outcome (PP)
**Figures**	
1.	Kaplan-Meier plot for mosquito mortality at Day 28 from Day 0 feed by arm
2.	Kaplan-Meier plot for mosquito mortality at Day 28 from Day 2+4h feed by arm
3.	Kaplan-Meier plot for mosquito mortality at Day 28 from Day 7 feed by arm
4.	Kaplan-Meier plot for mosquito mortality at Day 28 from Day 10 feed by arm
5.	Kaplan-Meier plot for mosquito mortality at Day 28 from Day 14 feed by arm
6.	Kaplan-Meier plot for mosquito mortality at Day 28 from Day 21 feed by arm

7.	Kaplan-Meier plot for mosquito mortality at Day 28 from Day 28 feed by arm
8.	Kaplan-Meier plot for mosquito mortality at Day 28 from 0 mcg arm by visit
9.	Kaplan-Meier plot for mosquito mortality at Day 28 from 300 mcg arm by visit
10.	Kaplan-Meier plot for mosquito mortality at Day 28 from 600 mcg arm by visit
11.	Kaplan-Meier plot for mosquito mortality at Day 28 from Day 7 feed by arm and feeding method
