# Supplementary material for: Efficacy and Safety of High-Dose Ivermectin for Reducing Malaria Transmission (IVERMAL): Protocol for a Double-Blind, Randomized, Placebo-Controlled, Dose-Finding Trial in Western Kenya
Source: JMIR Res Protoc. 2016 Nov 17;5(4):e213. doi: 10.2196/resprot.6617 (PMC5133431; doi:10.2196/resprot.6617)
Supplement: Multimedia Appendix 4 [file resprot_v5i4e213_app4.pdf]

KENYA MEDICAL RESEARCH INSTITUTE
CENTRE FOR GLOBAL HEALTH RESEARCH
23 FEB 2016
P.O. Box 1578, KISUMU

KEMRI

KENYA MEDICAL RESEARCH INSTITUTE

P.O. Box 54840-00200 NAIROBI - Kenya
Tel: (254) (020) 2722541, 254 (020) 2713349, 0722-205901, 0733-400003 Fax (254) (020) 2720030
Email: director@kemri.org info@kemri.org Website: www.kemri.org

KEMRI/RES/7/3/1 **February 16, 2016**

TO: **DR. MENNO SMIT & DR. ERIC OCHOMO,**
PRINCIPAL INVESTIGATORS

Through **DR. STEPHEN MUNGA,**
THE DIRECTOR, CGHR,
KISUMU

FORWARDED
24/2/2016
DIRECTOR
CENTRE FOR GLOBAL HEALTH RESEARCH

Dear Sirs,

RE: SSC PROTOCOL NO. 2775 (*RESUBMISSION OF EXPEDITED REQUEST FOR AMENDMENT 2)*: EFFICACY AND SAFETY OF HIGH–DOSE IVERMECTIN FOR REDUCING MALARIA TRANSMISSION: A DOSE FINDING STUDY-(*VERSION 4.1 DATED 14TH JANUARY, 2016*)

Reference is made to your letter dated 28th January, 2016 requesting for addition of protocol version that was approved. The KEMRI/Scientific and Ethics Review Unit (SERU) acknowledges receipt on 10th February, 2016.

This is to inform you that the Committee determines that the issues raised by the Expedited Review Team on 14th December, 2015 are adequately addressed. You are therefore **authorized** to implement the following Amendments accordingly:

1. Addition of four study sites: The facilities lie near the main trial site and would allow the study to recruit sufficient patients to finish the study within the study period.

Please note that you are responsible for submitting any further changes to the approved version of the study protocol to SERU for review and the changes should not be initiated until written approval from the SERU is received.

Yours faithfully,

DR. EVANS AMUKOYE,
ACTING HEAD,
KEMRI /SCIENTIFIC AND ETHICS REVIEW UNIT

DEPARTMENT OF HEALTH & HUMAN SERVICES

Public Health Service
Centers for Disease Control
and Prevention (CDC)

Memorandum

Date February 10, 2016

From Jason Abel
IRB Administrator, Human Research Protection Office

Subject CDC Approval of Continued Reliance on a Non-CDC IRB for CDC Protocol #6720: “Efficacy and safety of high-dose ivermectin for reducing malaria transmission: A dose finding study”

To AARON SAMUELS
CGH/DPDM

CDC's Human Research Protection Office has reviewed and approved the request to allow continued reliance on a non-CDC IRB for CDC protocol #6720 “Efficacy and safety of high-dose ivermectin for reducing malaria transmission: A dose finding study” in accordance with 45 CFR 46.114. The protocol has been reviewed and approved by Western IRB for the maximum allowable period of one year, and the IRB’s **approval will expire on 02/08/2017.**

Please submit CDC form 0.1251, Request for Continuing Review of IRB-Approved Protocol, along with certification of current IRB review and approval at the relied-upon institution, approximately six weeks prior to the protocol's expiration date. If you do not yet have certification of continuation approval to include with your submission, please state on the 0.1251 that certification of continuation approval will be forwarded as soon as it is received.

Any problems of a serious nature should be brought to the immediate attention of the Human Research Protection Office.

If you have any questions, please contact your National Center Human Subjects Contact or the CDC Human Research Protection Office at (404) 639-7570 or via e-mail or e-mail: huma@cdc.gov.

cc:
CGH Human Subjects (CDC)

Menno Smit
PO Box 1578
Kisumu 40100
Kenya

LSTM
LIVERPOOL SCHOOL OF TROPICAL MEDICINE

Pembroke Place,
Liverpool, L3 5QA, UK
Tel: +44(0)151 705 3100
Fax: +44(0)151 705 3370

www.lstmed.ac.uk

Wednesday, 03 February 2016

Dear Dr Smit,

Research Protocol (14-002) Efficacy and safety of high-dose Ivermectin in reducing malaria transmission

Thank you for your letter of 28th January 2016 providing the committee with details of the amendment to Ivermal Protocol v4.1 2016-01-14.

This amendment has now been reviewed, noted and accepted on the behalf of the committee. Please continue to adhere to the conditions of approval and to update us of any further changes to the study that may arise.

Yours sincerely,

Angela Obasi

Dr Angela Obasi
Chair
LSTM Research Ethics Committee

Researching and educating to save lives
A Company Limited by Guarantee. Registered Number 83405, England and Wales. Registered Charity Number 222655.

Athena SWAN Bronze Award

INVESTORS IN PEOPLE

MINISTRY OF HEALTH

Telegrams: "MEDICAL", Kisumu
Telephone: 057-2020801/2020803/2020321
Fax: 057-2024337
E-mail: ercjootrh@gmail.com
When replying please quote

Ref: ERC.1B/VOL.I/237

JARAMOGI OGINGA ODINGA TEACHING & REFERRAL HOSPITAL
P.O. BOX 849
KISUMU

Date: 3rd February, 2016

Dr. Menno Smit
KEMRI/CDC

RE: FORMAL APPROVAL FOR AMMENDMENT FOR CLINICAL TRIAL ENTITLED: "EFFICACY AND SAFETY OF HIGH-DOSE INVERMECTIN FOR REDUCING MALARIA TRANSMISSION: A DOSE FINDING STUDY"

The JOOTRH ERC (ACCREDITATION NO. 01713) has reviewed your request to amend some changes in the above research and found it satisfactory. You are therefore permitted continue with your research. Note that this amendment is granted.

The below has been amended:

Protocol Version	Details of change	Date
4.1	Clarification of the sampling strategy of additional sites	14th January, 2016

Please note that you will be required to share the findings of the study in both hard and soft copies upon completion.

The JOOTRH ERC takes this opportunity to thank you for choosing the institution and wishes you the best in your endeavours.

Yours sincerely,

MAKUNDA NANCY
FOR: SECRETARY - ERC,
JOOTRH – KISUMU.

JOOTRH ETHICS & REVIEW COMMITTEE
P. O. Box 849 - 40100
KISUMU
